# Ionenes as Potential Phase Change Materials with Self-Healing Behavior

**DOI:** 10.3390/polym15224460

**Published:** 2023-11-19

**Authors:** Carolina Arriaza-Echanes, María V. Velázquez-Tundidor, Alejandro Angel-López, Ángel Norambuena, Francisco E. Palay, Claudio A. Terraza, Alain Tundidor-Camba, Pablo A. Ortiz, Deysma Coll

**Affiliations:** 1 Vicerrectoría de Investigación, Universidad Mayor, Camino la Pirámide 5750, Santiago 8580745, Chileangel.norambuena@mayor.cl (Á.N.); francisco.palay@mayor.cl (F.E.P.); 2 Research Laboratory for Organic Polymers (RLOP), Department of Organic Chemistry, Pontificia Universidad Católica de Chile, Santiago 7820436, Chile; 3 Instituto de Investigaciones y Control del Ejército de Chile (IDIC), Santiago 8370899, Chile; 4 UC Energy Research Center, Pontificia Universidad Católica de Chile, Santiago 7820436, Chile; 5 Escuela de Ingeniería en Medio Ambiente y Sustentabilidad, Facultad de Ciencias, Ingeniería y Tecnología, Universidad Mayor, Camino La Pirámide 5750, Santiago 8580745, Chile; 6 Centro de Nanotecnología Aplicada, Facultad de Ciencias, Ingeniería y Tecnología, Universidad Mayor, Camino La Pirámide 5750, Santiago 8580745, Chile; 7 Núcleo de Química y Bioquímica, Facultad de Ciencias, Ingeniería y Tecnología, Universidad Mayor, Camino La Pirámide 5750, Santiago 8580745, Chile

**Keywords:** ionene, solid–solid phase change material, polyl(ionic liquids), self-healing

## Abstract

Ionenes are poly(ionic liquids) (PILs) comprising a polymer backbone with ionic groups along the structure. Ionenes as solid–solid phase change materials are a recent research field, and some studies have demonstrated their potential in thermal dissipation into electronic devices. Eight ionenes obtained through Menshutkin reactions were synthesized and characterized. The analysis of the thermal tests allowed understanding of how the thermal properties of the polymers depend on the aliphatic nature of the dihalogenated monomer and the carbon chain length. The TGA studies concluded that the ionenes were thermally stable with T_10%_ above 420 °C. The DSC tests showed that the prepared ionenes presented solid–solid transitions, and no melting temperature was appreciated, which rules out the possibility of solid–liquid transitions. All ionenes were soluble in common polar aprotic solvents. The hydrophilicity of the synthesized ionenes was studied by the contact angle method, and their total surface energy was calculated. Self-healing behavior was preliminarily explored using a selected sample. Our studies show that the prepared ionenes exhibit properties that make them potential candidates for applications as solid–solid phase change materials.

## 1. Introduction

Ionic liquids (ILs) behave as liquid organic salts below 100 °C, and the ionic interactions between cations and anions give them many interesting characteristics, such as thermal stability and low volatility [1,2,3]. These characteristics lead to the fact that ILs cover a wide field of applications because with the appropriate combination of cations and anions it is possible to modulate their final properties [4,5]. For the case of ILs, both inorganic and organic ions can be used for their synthesis [6,7] Thus, ILs have been studied and employed as chemical solvents [8,9], electrolytes [10,11,12] and even catalysts [13,14,15]. Additionally, they have been used in gas capture and separation, making them desirable for removing greenhouse gases, volatile organic compounds (VOCs) and heavy metal ions [16,17,18,19,20,21,22,23,24].

Many anion/cation combinations have been reported by different authors, showing that ILs have a wide range of phase transfer temperatures from −96 to 359 °C, making them desirable as phase change materials (PCMs) [25,26]. Ionic liquids used as PCMs show a solid–liquid transition, which limits their potential for some applications despite improving stability and thermal conductivity compared with conventional inorganic PCMs [25,27]. However, PCMs with solid–solid transitions (SS-PCM) have been prepared from poly(ionic liquids) (PILs), which are considered polyelectrolytes and have properties of polymers and ILs [28,29,30].

Ionenes are a type of PIL comprising a polymeric backbone with ionic groups along the structure. These groups allow the ionene to exchange ions with the environment in response to an external stimulus, such as a change in temperature or an electrical impulse [31,32]. Ionic concentration changes in a material can alter its optical, electrical and mechanical properties, which together with their greater flexibility and processability over traditional inorganic PCMs used in electronic devices [31], makes them a potential candidate for use in phase change memory devices.

Although research into ionenes as PCMs is very recent, some studies have demonstrated their potential for thermal dissipation in electronic devices. Cui et al. conducted structure–property relationship research of new PILs obtained from imidazolium-based norbornene derivatives in which they proved that a decrease in the size of the counterion results in an intense aggregation of the ions and an increase in the value of the glass transition temperature (T_g_) [33]. In fact, other structure modifications allow the modelling of PILs’ macroscopic properties, such as varying the distance between the charges, the type and size of the counterion, and the structure of the polymer main chain [33,34].

The charge density of the PIL chain depends on the size of the spacer group. A longer or shorter length can affect thermal properties, such as degradation, melting or crystallization temperature. This is because many charges will increase the ionic interactions between the cation and the anion of the ionene, but if they are very close, a repulsion may occur between charges of the main chain concerning the counterion used [35,36]. The type and size of the PIL counterion affects its ability to interact with the cation, facilitating or impeding the ordering of the chains, resulting in amorphous or crystalline structures and directly affecting its thermal properties [37].

The present work shows the synthesis of eight ionic polymers through the Menshutkin reaction. This polymerization involves the reaction between a primary dihalide and a tertiary amine. This particular class of SN2 reaction consists of the alkylation of a formally sp2 or sp3 hybridized nitrogen atom transforming a tertiary amine into a quaternary amine and obtaining ionic products from neutral reactants [38]. Polymers [PMDA-API-*p*Xy] [NTf_2_], [ODPA-API-*p*Xy] [NTf_2_], [BPDA-API-*p*Xy] [NTf_2_], [PMDA-API-C_3_] [NTf_2_], [ODPA-API-C_3_] [NTf_2_] and [BPDA-API-C_3_] [NTf_2_] were characterized spectroscopically, and their thermal properties were studied as possible candidates as PCMs. Considering the intermediate T_g_ values presented by [BPDA-API-C_3_] [NTf_2_], it was selected as a point of comparison to two other polymers ([BPDA-API-C_6_] [NTf_2_] and [BPDA-API-C_12_] [NTf_2_]) with different spacer chain lengths (with 6 and 12 carbon atoms, respectively). The analysis of the thermal tests allowed a study of the effect of the aliphatic nature of the dihalogenated monomer and the length of the carbon chain on the thermal properties of the obtained PILs. In addition, the hydrophilic/hydrophobic behavior of the samples was studied and the self-healing properties of [BPDA-API-C_12_] [NTf_2_] were established.

## 2. Experimental Part

### 2.1. Materials and Equipment

Reagents and solvents were purchased from Sigma Aldrich-Merck, except lithium bis(trifluoromethane)sulfonimide (LiNTf_2_), which was obtained from AK Scientific. All chemicals were used as received. Melting points were determined with SMP3 equipment-Stuart Scientific (ALT, San Diego, CA, USA). FT-IR-ATR (ZnSe) spectra were recorded on a Spectrum Two (Perkin Elmer, Massachusetts, U.S.) spectrophotometer over the range of 4000–600 cm^−1^. ^1^H, ^13^C, ^19^F and dept 135° NMR spectra were recorded on a Bruker Advance III-400 Hz espectrometer (Bruker Corporation, Karlsruhe, Germany) using DMSO-*d_6_* and D_2_O as solvent. Thermal stability of the membranes was evaluated using a TGA-50 SHIMADZU thermogravimetric analyzer (Columbia, USA). Analysis was performed using a temperature range of 30–800 °C, under nitrogen atmosphere and a heating ramp of 20 °C/min. The glass transition temperature of the membranes was determined using a PerkinElmer DSC 4000 differential scanning calorimeter from the second scan (Perkin Elmer, Massachusetts, U.S.). The measurements were from −50 °C to 300 °C at a speed of 10 °C/min, with a nitrogen atmosphere with a flow of 20 mL/min. A contact angle test was carried out at 20 °C using Krüss DSA 255 equipment, and the images were analyzed with the Krüss Advance program (Hamburg, Germany). The tests were carried out using water as a polar solvent and diidomethane as a non-polar solvent. Twenty-four readings of each polymer were carried out with both solvents, and the averages for each ionene were recorded.

### 2.2. Film Preparation

Each ionene (100 mg) was dissolved in 1 mL of acetone, filtered through a 3.1 μm fiberglass Synta^®^ syringe filter, and poured over a glass dish on a leveled surface. The glass dish with the ionene solution was covered with a funnel to avoid contamination by dust and left at room temperature for 24 h. Then, the film was peeled off and put into an oven at 40 °C for 24 h.

### 2.3. Self-Healing Test

Each ionene (1 g) was placed on a Teflon plate and heated up to 100 °C above Tg to make a rectangular piece (1.5 × 2.0 cm, thickness of 2.4–3.8 mm). Only ionenes [ODPA-API-pXy] [NTf_2_], [ODPA-API-C_3_] [NTf_2_], [BPDA-API-C_3_] [NTf_2_], [BPDA-API-C_6_] [NTf_2_] and [BPDA-API-C_12_] [NTf_2_] were thermomolded. Then, self-healing capacity was evaluated by cutting each ionene and heating them at 70 °C and 100 °C above T_g_ for 2 and 10 min.

### 2.4. Monomer Synthesis

**Scheme 1:**
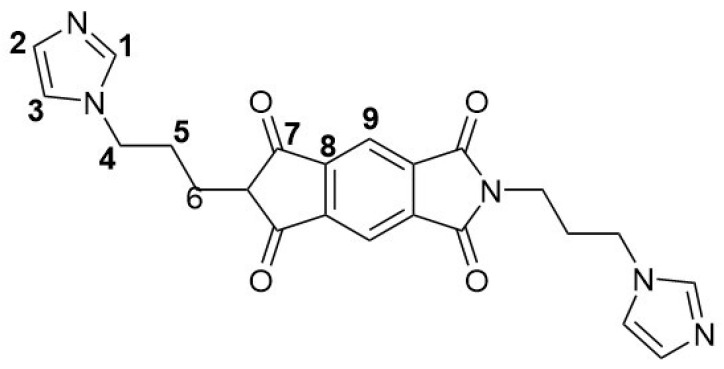
PMDA-API.

**Scheme 2:**
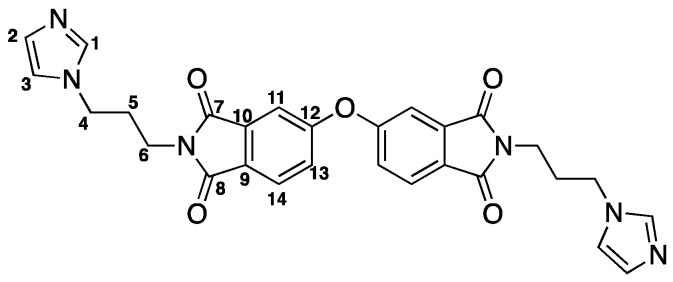
ODPA-API.

**Scheme 3:**
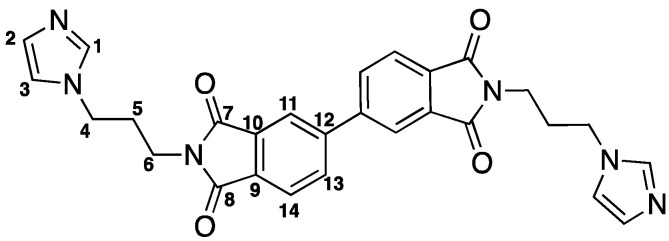
BPDA-API.

General procedure for the obtention of diimidazolium monomers (PMDA-API, ODPA-API and BPDA-API): In a 100 mL round-bottom flask was added 70 mmol of dianhydride (1*H*,3*H*-benzo[1,2-*c*:4,5-*c*’]difuran-1,3,5,7-tetraone (PMDA), 5,5′-oxybis(isobenzofuran-1,3-dione) (ODPA) or [5,5′-biisobenzofuran]-1,1′,3,3′-tetraone) (BPDA), 147 mmol of 1-(3-aminopropyl)imidazole (API, 5% excess) and 45 mL of DMF. The reaction mixture was heated at 130 °C for 24 h. After this time, the reaction was cooled down to room temperature, and a solid appeared, which was removed by filtration, washed with plenty of water and placed in a vacuum oven at 85 °C for 24 h. The experimental setup used is shown in Figure S1. The monomers were characterized (Scheme 1, Scheme 2 and Scheme 3) and recrystallized (PMDA-API [H_2_O], ODPA-API [ethanol/H_2_O] and BPDA-API [DMF]).

White solid. Yield: 95%. Mp: 215 °C. ATR-IR (ʋ, cm^−1^): 3125, 3104 (C-H, arom.); 2989, 2971, 2941, 2903 (C-H, aliph.); 1772, 1700 (C=O); 1511, 1462, 1445 (C=C); 1390 (C-N); 726 (imide ring bending). ^1^H NMR (DMSO-*d_6_*, δ, ppm): 8.18 (s, 2H, **9**); 7.65 (s, 2H, **1**); 7.20 (s, 2H, **3**); 6.88 (s, 2H, **2**); 4.05 (t, *J* = 6.9 Hz, 4H, **4**); 3.61 (t, *J* = 6.7 Hz, 4H, **6**); 2.15–2.05 (m, 4H, **5**). ^13^C NMR (DMSO-*d_6_*, δ, ppm): 166.43 (**7**); 137.35 (**1**); 137.02 (**8**); 128.38 (**2**); 119.33 (**3**); 117.14 (**9**); 43.71 (**4**); 35.52 (**6**); 29.39 (**5**). The spectra are shown in Figure S2.

Yellow solid. Yield: 87%. Mp: 167–169 °C. ATR-IR (ʋ, cm^−1^): 3148, 3119, 3096, 3064, 3022 (C-H, arom.); 2971, 2945, 2939, 2917 (C-H, aliph.); 1768, 1703 (C=O); 1064 (C=N); 1508, 1473, 1441 (C=C); 1389 (C-N); 1272 (C-O); 743 (imide ring bending); 663 (tri-subst. arom.). ^1^H NMR (DMSO-*d_6_*, δ, ppm): 7.95 (d, *J* = 7.9 Hz, 2H, **14**); 7.64 (s, 2H, **1**); 7.56–7.50 (m, 2H, **13**); 7.51 (s, 2H, **11**); 7.20 (s, 2H, **3**); 6.88 (s, 2H, **2**); 4.03 (t, *J* = 7.1 Hz, 4H, **4**); 3.56 (t, *J* = 6.7 Hz, 4H, **6**); 2.08–1.98 (m, 4H, **5**). ^13^C NMR (DMSO-*d_6_*, δ, ppm): 167.25, 167.06 (**7, 8**); 160.56 (**12**); 137.28 (**1**); 134.63 (**10**); 128.35 (**2**); 127.34 (**9**); 125.57 (**1**); 124.42 (**11**); 119.24 (**3**); 113.37 (1**3**); 43.68 (**4**); 35.10 (**6**); 29.63 (**5**). The spectra are shown in Figure S3.

White solid. Yield: 96%. Mp: 223 °C. ATR-IR (ʋ, cm^−1^): 3112, 3082, 3068, 3025 (C-H, arom.); 2982, 2954, 2943, 2869 (C-H, aliph.); 1765, 1700 (C=O); 1627, 1619 (C=N); 1509, 1491, 1438, 1426 (C=C); 1388 (C-N); 745 (imide ring bending). ^1^H NMR (DMSO-*d_6_*, δ, ppm): 8.28–8.24 (m, 4H, **11,13**); 7.96 (d, *J* = 8.3 Hz, 2H, **14**); 7.64 (s, 2H, **1**); 7.20 (s, 2H, **3**); 6.88 (s, 2H, **2**); 4.04 (t, *J* = 6.9 Hz, 4H, **4**); 3.58 (t, *J* = 6.8 Hz, 4H, **6**); 2.13–2.02 (m, 4H, **5**). ^13^C NMR (DMSO-*d_6_*, δ, ppm): 167.61 (**7, 8**); 144.13 (**12**); 137.32 (**1**); 133.29 (**13**); 132.90 (**10**); 131.56 (**9**); 128.40 (**14**); 123.75 (**2**); 121.81 (**11**); 119.33 (**3**); 43.71 (**4**); 35.11 (**6**); 29.69 (**5**). The spectra are shown in Figure S4.

### 2.5. Ionene Synthesis

**Scheme 4:**
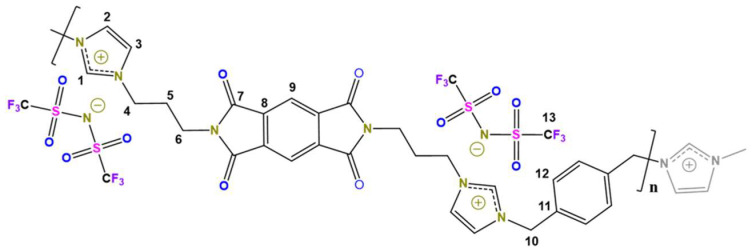
[PMDA-API-pXy] [NTf_2_].

**Scheme 5:**
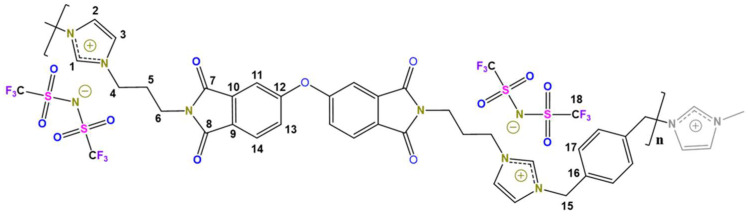
[ODPA-API-pXy] [NTf_2_].

**Scheme 6:**
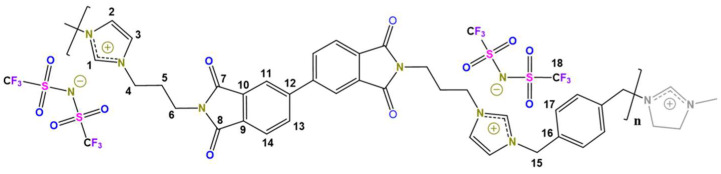
[BPDA-API-pXy] [NTf_2_].

**Scheme 7:**
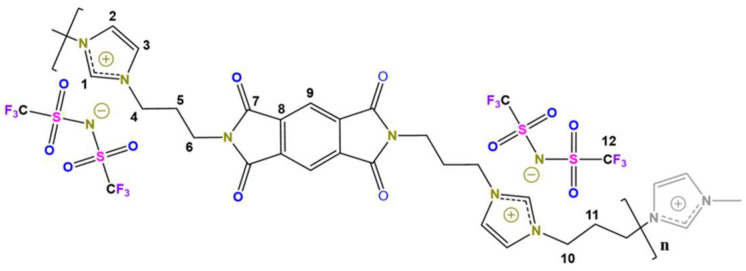
[PMDA-API-C_3_] [NTf_2_].

**Scheme 8:**
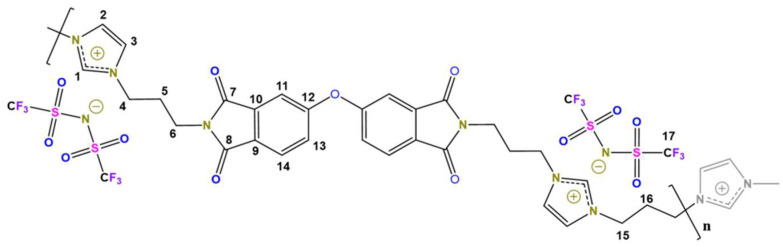
[ODPA-API-C_3_] [NTf_2_].

**Scheme 9:**
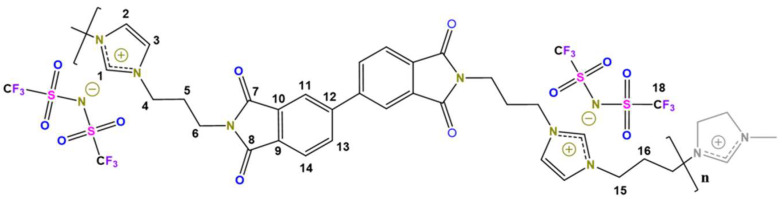
[BPDA-API-C_3_] [NTf_2_].

**Scheme 10:**
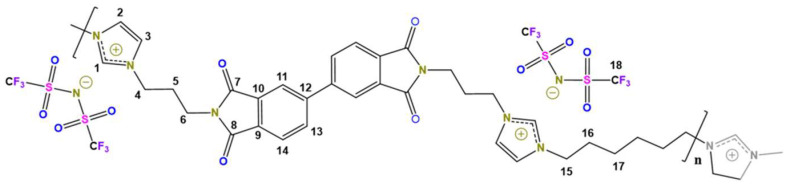
[BPDA-API-C_6_] [NTf_2_].

**Scheme 11:**
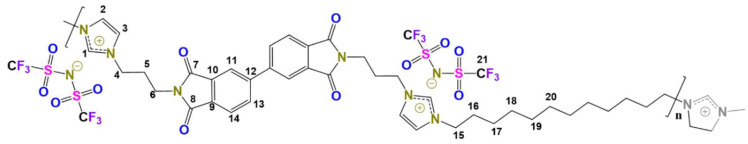
[BPDA-API-C_12_] [NTf_2_].

Into a 60 mL pressure vessel was added 4.0 mmol of monomer, 4.0 mmol of the respective dibromide compound (1,4-bis(bromomethyl)benzene, 1,3-dibromopropane, 1,6-dibromohexane or 1,12-dibromododecane) and 20 mL of DMF. The tube was closed, and the reaction was left for 24 h at 100 °C with magnetic stirring. After this time, 8.4 mmol of lithium bis(trifluoromethanesulfonyl)imide (5% excess) was added while the reaction remained hot, and then the mixture was left for another 24 h at 100 °C under stirring. The reaction mixture was cooled to room temperature, and dropped into 200 mL of distilled water, causing precipitation of the ionene. Subsequently, the solid was isolated by filtration and dried at 60 °C for 24 h in a vacuum oven. Ionenes were reprecipitated by dissolving them in acetonitrile and adding water dropwise. The experimental setup used is shown in Figure S1B. The Ionenes were characterized (Scheme 4, Scheme 5, Scheme 6, Scheme 7, Scheme 8, Scheme 9, Scheme 10 and Scheme 11).

Yield: 93%. ATR-IR (ʋ, cm^−1^): 3147, 3114, 3087 (C-H, arom.); 2948 (C-H, aliph.); 1773, 1712 (C=O); 1561, 1455, 1449 (C=C); 1345 (C-N); 1326 (SO_2_, asym.); 1225 (CF_3_, asym.); 1175 (C-N); 1131 (SO_2_; sym.); 1050 (S-N-S); 727 (imide ring bending). ^1^H NMR (DMSO-*d_6_*, δ, ppm): 9.44 (s, 2H, **1**); 8.18 (s, 2H, **9**); 7.85 (s, 4H, **2, 3**); 7.51 (s, 4H, **12**); 5.49 (s, 4H, **10**); 4.31 (t, *J* = 7.5 Hz, 4H, **4**); 3.68 (t, *J* = 6.6 Hz, 4H, **6**); 2.26–2.29 (m, 4H, **5**). ^13^C NMR (DMSO-*d_6_*, δ, ppm): 166.46 (**7**); 137.09 (**8**); 136.44 (**1**); 135.35 (**11**); 129.02 (**12**); 122.89 (**3**); 122.54 (**2**); 117.19 (**9 y 13**); 51.47 (**10**), 46.78 (**4**), 34.98 (**6**), 28.44 (**5**). ^19^F NMR (DMSO-*d_6_*, δ, ppm): −76.90. The spectra are shown in Figure S5.

Yield: 87%. ATR-IR (ʋ, cm^−1^): 3153, 3116, 3094 (C-H, arom.); 2955, 2920, 2848 (C-H, aliph.); 1772, 1708 (C=O); 1564, 1458, 1449, 1438 (C=C); 1344 (C-N); 1325 (SO_2_, asym); 1225 (CF_3_, asym); 1177 (C-N); 1131 (SO_2_; sym); 1051 (S-N-S); 726 (imide ring bending). ^1^H NMR (DMSO-*d_6_*, δ, ppm): 8.39 (s, 2H, **1**); 7.07 (d, *J =* 8.8 Hz, 2H, **14**); 6.90–6.8 (m, 4H, **2, 3**); 6.64–6.62 (m, 4H, **11, 13**); 6.57 (s, 4H, **17**); 3.34 (t, *J* = 7.1 Hz, 4H, **4**); 2.71 (t, *J* = 5.8 Hz, 4H, **6**); 1.28–1.23 (m, 4H, **5**). ^13^C NMR (DMSO-*d_6_*, δ, ppm): 167.34 (**7**); 167.13 (**8**); 160.65 (**12**); 136.47 (**1**); 135.33 (**16**); 134.69 (**10**); 128.95 (**17**); 127.39 (**9**); 125.73 (**14**); 124.47 (**11**); 122.95 (**3**); 122.54 (**2**); 121.12, 117.92 (**18**); 113.53 (**13**); 51.58 (**15**); 46.79 (**4**), 34.54 (**6**); 28.61 (**5**). ^19^F NMR (DMSO-*d_6_*, δ, ppm): −76.84. The spectra are shown in Figure S6.

Yield: 81%. ATR-IR (ʋ, cm^−1^): 3150, 3111, 3089 (C-H, arom.); 2933 (C-H, aliph.); 1772, 1704 (C=O); 1606 (C=N); 1559, 1475, 1441 (C=C); 1345 (C-N); 1325 (SO_2_, aym); 1231 (CF_3_, asym); 1178 (C-N); 1128 (SO_2_; sym); 1051 (S-N-S); 732 (imide ring bending). ^1^H NMR (DMSO-*d_6_*, δ, ppm): 9.28 (s, 2H, **1**); 8.28–8.23 (m, 4H, **11, 13**); 8.00 (d, *J* =7.1 Hz, 2H, **14**); 7.81–7.77 (m, 2H, **2, 3**); 7.48 (s, 4H, **17**); 4.27 (t, *J* = 7.1 Hz, 4H, **4**); 3.66 (t, *J* = 5.8 Hz, 4H, **6**); 2.23–2.18 (m, 4H, **5**). ^13^C NMR (DMSO-*d_6_*, δ, ppm): 167.70, 167.66 (**7, 8**); 144.25 (**12**); 136.49 (**1**); 135.34 (**16**); 134.43 (**13**); 132.96 (**10**); 131.63 (**9**); 128.97 (**17**); 123.90 (**14**); 122.98 (**3**); 122.556 (**2**); 121.87 (**11**); 121.13, 117.93 (**18**); 51.62 (**15**); 46.83 (**4**), 34.55 (**6**), 28.66 (**5**). ^19^F NMR (DMSO-*d_6_*, δ, ppm): −76.84 (**18**). The spectra are shown in Figure S7.

Yield: 89%. ATR-IR (KBr, ʋ, cm^−1^): 3150, 3116, 3096 (C-H, arom.); 2956, 2917, 2849 (C-H, aliph.); 1771, 1704 (C=O); 1667, 1607 (C=N); 1563, 1472, 1441 (C=C); 1345 (C-N); 1329 (SO_2_, asym); 1229 (CF_3_, asym); 1175 (C-N); 1131 (SO_2_; sym); 1052 (S-N-S); 747 (imide ring bending). ^1^H NMR (DMSO-*d_6_*, δ, ppm): 9.16 (s, 2H, **1**); 8.24 (s, 2H, **9**); 7.83–7.79 (m, 4H, **2, 3**); 0.28–4.26 (m, 8H, **4, 10**); 3.81–3.68 (m, 4H, **6**); 2.45–2.35 (m, 2H, **11**); 2.25–2.18 (m, 4H, **5**). ^13^C NMR (DMSO-*d_6_*, δ, ppm): 166.43 (**7**); 137.09 (**8**); 136.43 (**1**); 122.67, 116.28 (**12**); 122.62 (**2**); 122.49 (**3**); 117.21 (**9**); 46.71 (**4**); 45.98 (**10**); 34.94 (**6**); 29.47 (**11**); 28.46 (**5**). ^19^F NMR (DMSO-*d_6_*, δ, ppm): −76.84. The spectra are shown in Figure S8.

Yield: 92%. ATR-IR (KBr, ʋ, cm^−1^): 3148, 3114, 3091 (C-H, arom.); 2945, 2877 (C-H, aliph.); 1770, 1701 (C=O); 1660, 1607 (C=N); 1563, 1439 (C=C); 1347 (C-N); 1326 (SO_2_, asym); 1228 (CF_3_, asym); 1178 (C-N); 1132 (SO_2_; sym); 1051 (S-N-S); 739 (imide ring bending).^1^H NMR (DMSO-*d_6_*, δ, ppm): 9.15 (s, 2H, **1**); 7.96 (d, *J* = 7.1 Hz, 2H, **14**); 7.82–7.78 (m, 4H, **2, 3**); 7.53–7.51 (m, 4H, **11, 13**); 4.25 (t, *J* = 7.0 Hz, 8H, **4, 15**); 3.62 (t, *J* = 6.5 Hz, 4H, **6**); 2.45–2.38 (m, 2H, **16**); 2.21–2.14 (m, 4H, **5**). ^13^C NMR (DMSO-*d_6_*, δ, ppm): 167.36, 167.14 (**7, 8**); 136.47 (**1**); 134.68 (**10**); 127.398 (**9**); 125.70 (**14**); 124.42 (**11**); 122.64, 122.47 (**2, 3**); 121.10, 117.90 (**17**); 113.54 (**13**); 46.71 (**4**); 45.98 (**15**); 34.51 (**6**); 29.48 (**16**); 28.61 (**5**). ^19^F NMR (DMSO-*d_6_*, δ, ppm): −76.84. The spectra are shown in Figure S9.

Yield: 85%. ATR-IR (KBr, ʋ, cm^−1^): 3153, 3117, 3098 (C-H, arom.); 2950 (C-H, aliph.); 1771, 1704 (C=O); 1623 (C=N); 1564, 1442 (C=C); 1348 (C-N); 1325 (SO_2_, asym); 1224 (CF_3_, asym); 1176 (C-N); 1130 (SO_2_; sym); 1049 (S-N-S); 737 (imide ring bending). ^1^H NMR (DMSO-*d_6_*, δ, ppm): 9.17 (s, 2H, **1**); 8.30–8.27 (m, 4H, **11, 13**); 8.01 (d, *J* = 7.7 Hz, 2H, **14**); 7.85n (s, 2H), 7.80 (s, 2H) (**2**, **3**); 8.28–4.26 (m, 8H, **4, 15**); 3.70–3.67 (m, 4H, **6**); 2.44–2.41 (m, 2H, **16**); 2.24–2.20 (m, 4H, **5**). ^13^C NMR (DMSO-*d_6_*, δ, ppm): 167.66, 167.61 (**7, 8**); 144.19 (**12**); 136.45 (**1**); 133.37 (**13**); 132.91 (**10**); 131.59 (**9**); 123.81 (**14**); 122.63, 122.46 (**2, 3**); 121.83 (**11**); 121.07, 117.87 (**18**); 46.70 (**4**); 45.96 (**15**); 34.50 (**6**); 29.42 (**16**); 28.63 (**5**). ^19^F NMR (DMSO-*d_6_*, δ, ppm): −76.90. The spectra are shown in Figure S10.

Yield: 95%. ATR-IR (KBr, ʋ, cm^−1^): 3151, 3116, 3094 (C-H, arom.); 2955, 2917, 2871, 2849 (C-H, aliph.); 1770, 1705 (C=O); 1623 (C=N); 1564, 1440 (C=C); 1347(C-N); 1329 (SO_2_, asym); 1228 (CF_3_, asym); 1176 (C-N); 1130 (SO_2_; sym); 1053 (S-N-S); 737 (imide ring bending). ^1^H NMR (DMSO-*d_6_*, δ, ppm): 9.15 (s, 2H, **1**); 8.30–8.27 (m, 4H, **11, 13**); 8.01 (d, J = 7.6 Hz, 4H, **14**); 7.81–7.79 (m, 4H, **2, 3**); 4.26 (t, *J* = 7.4 Hz, 4H, **4**); 4.17 (t, *J* = 7.4 Hz, 4H, **15**); 3.69–3.71 (m, 4H, **6**); 2.20–2.16 (m, 4H, **5**); 1.83–1.79 (m, 4H, **16**); 1.31 (s, 4H, **17**). ^13^C NMR (DMSO-*d_6_*, δ, ppm): 167.61, 167.58 (**7, 8**); 144.18 (**12**); 136.12 (**1**); 133.36 (**13**); 132.91 (**10**); 131.59 (**9**); 123.80 (**14**); 122.47, 122.43 (**2, 3**); 121.82 (**11**); 121.07, 117.87 (**18**); 48.81 (**15**); 46.62 (**4**); 34.49 (**6**); 29.09 (**16**); 28.63 (**5**); 24.96 (**17**). ^19^F NMR (DMSO-*d_6_*, δ, ppm): −76.87. The spectra are shown in Figure S11.

Yield: 94%. ATR-IR (KBr, ʋ, cm^−1^): 3150, 3115, 3095 (C-H, arom.); 2926, 2853 (C-H, aliph.); 1771, 1708 (C=O); 1620 (C=N); 1563, 1441 (C=C); 1347 (C-N); 1331 (SO_2_, asym); 1228 (CF_3_, asym); 1182 (C-N); 1132 (SO_2_; sym); 1053 (S-N-S); 737 (imide ring bending). ^1^H NMR (DMSO-*d_6_*, δ, ppm): 9.16 (s, 2H, **1**); 8.30–8.27 (m, 4H, **11, 13**); 8.00 (d, *J* = 7.6 Hz, 2H, **14**); 7.80–7-79 (m, 4H, **2, 3**); 4.26 (t, *J* = 7.1 Hz, 4H, **4**); 4.15 (t, *J* = 6.6 Hz, 4H, **15**); 3.66 (t, *J* = 6.2 Hz, 4H, **6**); 2.20–2.17 (m, 4H, **5**); 1.81–1.76 (m, 4H, **16**); 1.23 (s, 16H, **17, 18, 19, 20**). ^13^C NMR (DMSO-*d_6_*, δ, ppm): 167.60, 167.58 (**7, 8**); 144.18 (**12**); 136.11 (**1**); 133.37 (**13**); 132.91 (**10**); 131.58 (**9**); 123.81 (**14**); 122.43 (**2, 3**); 121.83 (**11**); 121.08, 117.88 (**21**); 48.91 (**15**); 46.63 (**4**); 34.51 (**6**); 29.33 (**16**); 28.97, 28.87, 28.58, 25.54 (**17, 18, 19, 20**); 28.42 (**5**). ^19^F NMR (DMSO-*d_6_*, δ, ppm): −76.89. The spectra are shown in Figure S12.

## 3. Results and Discussion

### 3.1. Synthesis and Structural Characterization of Monomers and Ionenes

**Scheme 12:**
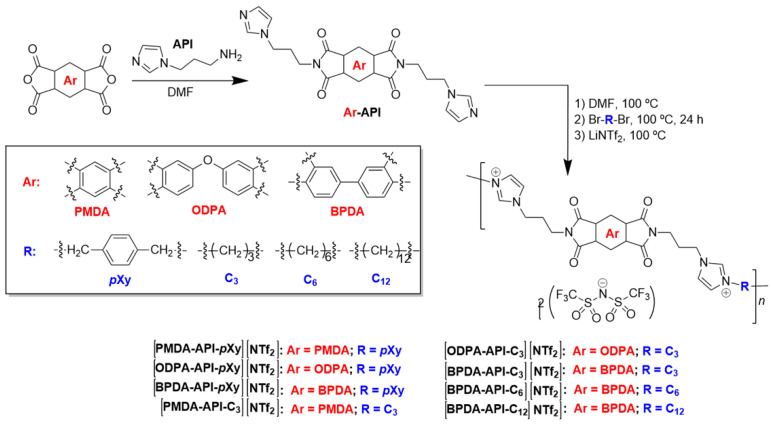
Synthetic route to obtain ionenes.

The monomers and PILs were obtained according to Scheme 12. Monomers were obtained with yields of between 87 and 96%, and were meticulously purified by recrystallization and subsequently characterized.

**Figure 1:**
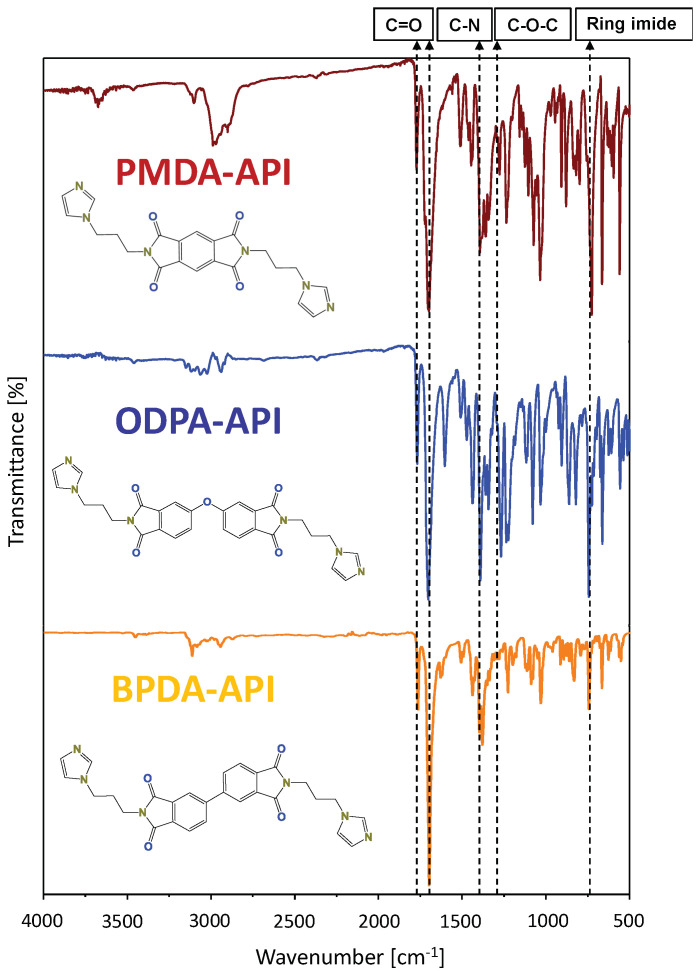
ATR-IR of PMDA-API (brown line), ODPA-API (blue line) and BPDA-API (orange line).

The FT-IR spectra recorded for the monomers were very similar (Figure 1). In these, it is possible to see three typical signals of the imide function that correspond to C=O stretching around 1780–1710 cm^−1^, C-N stretching around 1360 cm^−1^ and imide ring bending (around 740 cm^−1^). For ODPA-API, the C-O-C stretching band of the ether bond at ca. 1270 cm-1 stands out.

**Figure 2:**
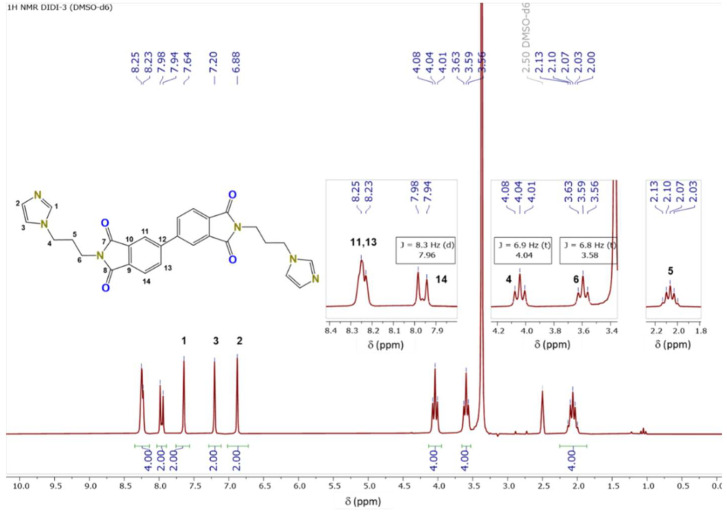
Full ^1^H NMR (DMSO-*d_6_*) and some expansions for BPDA-API.

The ^1^H NMR spectrum of BPDA-API is shown as an example in Figure 2. The signals corresponding to the aliphatic zone provided by IPA appear at high field, with multiplicity and integration in accordance with the propyl chain. At low field, the aromatic signals can be verified at 8.3–7.9 ppm (phenyl rings) and 7.7–6.7 ppm (imidazole rings) integrating for the 12 protons present in the structure.

From these monomers and the respective dibrominated derivatives, the ionenes were synthesized by modifying the methodology described by Bara et al. [39]. For this, a pressure vessel with a self-developed safety system was used (Figure S1B). Not all monomers were totally soluble in DMF at room temperature, so it was necessary to heat up to 100 °C. When the mixture was homogeneous, the dihalogenated monomer was added.

Different methods to carry out ion exchange are reported in the literature. Bara et al. describe in situ ion exchange during polymerization, for which they simultaneously add monomers and LiNTf_2_ to the reaction medium. However, for synthesizing ionic liquids of similar structure, obtaining the cation and its subsequent isolation is reported [39].

For the exchange, the authors prepared an aqueous solution of the brominated compound that was poured onto an aqueous solution of LiNTf_2_. Both methods were tested in this work. In the first case, the polymers obtained did not form self-standing films, probably due to the low molecular weight of the polymers. The second method allowed the authors to obtain materials capable of creating self-standing films, but in low yields (70%). This was attributed to the loss of material during ion exchange, considering that the polymers with bromine as a counterion were soluble in water. For this reason, a third route was tested in which the brominated ionene was not isolated. In this procedure, at the end of the polymerization, LiNTf_2_ was added to carry out the exchange in the reaction medium. The yields ranged from 81 to 95%, and the ionenes formed good self-standing films.

To purify the ionenes, acetonitrile was used as a solvent and water was used as a precipitating agent. This procedure allowed the authors to obtain an efficiency purification of around 90% and eliminate the smaller chains formed during the polymerization.

**Figure 3:**
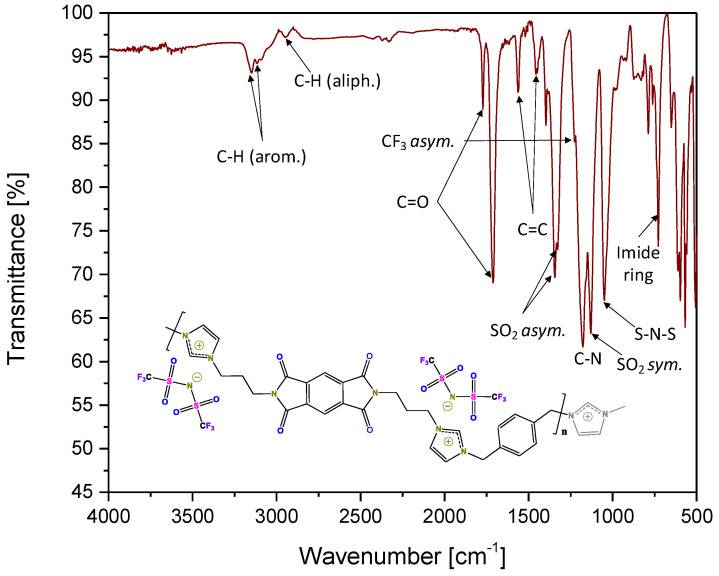
ATR-IR of ionene [PMDA-API-pXy] [NTf_2_].

All ionenes were characterized by ATR-IR and NMR analysis, and their respective spectra are available in the Supplementary Materials (Figures S5–S12). In each IR spectrum, it was possible to observe the presence of the characteristic bands of the proposed repetitive unit, as shown in Figure 3 for [PMDA-API-pXy] [NTf_2_]. These bands correspond to the signals of C=O stretching of the imide carbonyl group between 1773 and 1710 cm^−1^, C-N stretching (≈1350 cm^−1^) and imide ring bending (≈730 cm^−1^). Another important signal in these compounds is the asymmetric stretching of the SO_2_ moiety (ca. 1330 cm^−1^), the asymmetrical ones of CF_3_ (around 1230 cm^−1^), the symmetrical ones of SO_2_ (around 1130 cm^−1^) and the stretching corresponding to the S-N-S system (≈1050 cm^−1^). The latter signals came from the characteristic bonds of the NTf_2_ counterion.

In the ^1^H NMR spectrum of ionenes, aromatic protons were observed at between 9.5 ppm and 6.5 ppm that correspond to hydrogens that are part of the aromatic aryl imide and imidazolium rings. For the latter aromatic system, the consistent pattern of a singlet for H1 at low field (N-CH-N) and a multiplet for the overlapping H2 and H3 signals (N-CH=CH-N) was present in all spectra with small differences in their chemical displacement. This is because the hydrogens of the imidazolium ring present a similar magnetic environment in each synthesized ionene. Contrary to this, for aromatic systems based on benzene rings, signals between 8.4 ppm and 6.5 ppm of varying multiplicity and chemical displacements were observed in the proton spectra.

Aliphatic protons were readily observed in high field between 5.5 ppm and 1.1 ppm according to the carbon chain length of the dibrominated derivative used in the synthesis. In this sense, a signal pattern composed of H4, H6 and H5 of the imidazolium moiety is present in all spectra, with a triplet for the first two and a multiplet for H5. Due to their cationic nature, the imidazolium ring is highly electronegative, which causes a shift of the signals of the methylene hydrogens to low field.

In the ^13^C NMR spectra, all the expected carbons signals were observed. In the cases where the carbonyl groups of imide moiety are magnetically equivalent ([PMDA-API-*p*Xy] [NTf_2_] and [PMDA-API-C_3_] [NTf_2_]), these were observed by a signal at ca. 167 ppm, while chemical non-equivalence ([ODPA-API-*p*Xy] [NTf_2_], [BPDA-API-*p*Xy] [NTf_2_], [ODPA-API-C_3_] [NTf_2_] and [BPDA-API-C_3_-C_6_] [NTf_2_]) manifested itself in two very close signals near 167 ppm and 168 ppm. The imidazolium carbon atoms were observed as one signal at ca. 136.5 ppm for the most unprotected position (N-CH-N) and two signals around 122.5 ppm for C2 and C3.

The number and chemical shift observed for the aliphatic signals agreed with the invariant nature of the propyl chain of the imidazolium moiety and the variation in chain length contributed by the dibromide compound employed in the polymerization. Thus, the first three were observed with very little variation in their chemical shift: 46.7 ppm (C4), 34.6 ppm (C6) and 28.6 ppm (C5). On the other hand, the methylene located between the benzene ring and the imidazolium ring of [PMDA-API-*p*Xy] [NTf_2_], [ODPA-API-*p*Xy] [NTf_2_] and [BPDA-API-*p*Xy] [NTf_2_] was observed at about 51.5 ppm. Replacing p-xylenyl moiety by a propyl chain ([PMDA-API-C_3_] [NTf_2_], [ODPA-API-C_3_] [NTf_2_] and [BPDA-API-C_3_] [NTf_2_]) generated the signals observed at 29.5 ppm and 45.9 ppm, where the latter corresponds to the methylene neighboring the imidazolium ring. A similar effect was observed in [BPDA-API-C_6_] [NTf_2_] and [BPDA-API-C_12_] [NTf_2_], where the alluded methylene carbon (C15) is observed strongly displaced at low field, at 48.81 ppm and 48.91 ppm, respectively. In all spectra, the fluorinated carbon promoted by the NTf_2_ counterion was observed as a doublet with signals near 121 ppm and 117 ppm.

All assignments were carried out using NMR-dept-135° spectra (Supplementary Materials) and MestReNova 14.0 software as supporting elements.

Additionally, ^19^F NMR analysis was performed on each ionene, and all of them showed a signal at ca. −76.9 ppm, reinforcing the existence of the NTf_2_ counterion in the synthesized ionenes.

**Figure 4:**
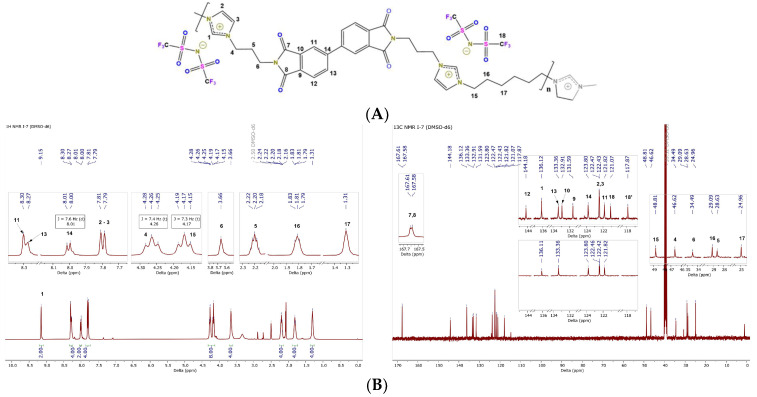
Full spectra and expansions for [BPDA-API-C_6_] [NTf_2_]in DMSO-*d_6_*. (**A**) ^1^H NMR and (**B**) ^13^C NMR.

As an example of the characterization developed by NMR techniques, Figure 4 shows the ^1^H and ^13^C NMR spectra for [BPDA-API-C_6_] [NTf_2_].

### 3.2. Solubility

Table 1 shows the results of the ionene solubility test performed at room temperature. All polymers were soluble in polar aprotic solvents (DMSO, DMF, acetonitrile and acetone), while they were insoluble in water and low-molecular-weight alcohols. In this sense, and despite their ionic component present in the oinenes, the high aromatic content and the presence of the NTf_2_ counterion with elements of hydrophobic nature (CF_3_) prevent an effective solvation of the chains. The structural differences in the main chain of the PILs did not affect the solubility behavior. Similar results were obtained when intermediate polarity solvents (ethyl acetate, chloroform and diethyleter) were used.

### 3.3. Thermal Properties

**Figure 5:**
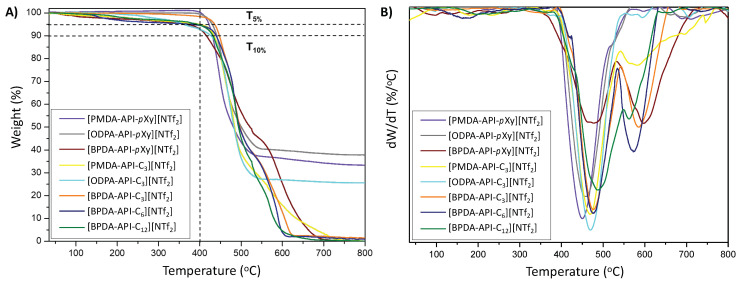
(**A**) Thermograms for ionenes. (**B**) DTGA curves for ionenes.

TGA and DSC analyses were developed to determine the thermal behavior of ionene under an inert atmosphere (N_2_). TGA curves are shown in Figure 5, and a summary of the most important thermal parameters (T_onset_, T_5%_, T_10%_, T_d_ and R) is shown in Table 2.

The TGA curves showed how the ionenes start a degradative process above 342 °C, losing 5% and 10% of their mass between 360 and 430 °C and between 427 and 484 °C, respectively. These results are consistent with the molecular architecture of the ionenes, which are composed mainly of aromatic groups such as phenyls, aryl-imide and imidazonium rings, as well as ionic groups (NFT_2_ counterion and the imidazolium fragment) [40,41]. Thus, it is possible that the ionic groups along the main chain provide additional thermal resistance derived from their strong ionic interactions. However, no decrease in degradation temperature was observed with increasing aliphatic fragment length, a fact commonly observed in several nonionic and ionic polymers [42,43].

The maximum degradation rate value (T_d1_) recovered for the ionenes, which was found to be above 450 °C, confirmed the high thermal resistance of the samples. Moreover, the ionenes prepared from [5,5′-biisobenzofuran]-1,1′,3,3′-tetraone (BPDA), a compound that allowed incorporation of a biphenyl segment into the main chains, showed a second degradation process between 560 and 597 °C (T_d2_). Since the biphenyl unit is the only structural difference, it suggests that during the degradation process, the lack of conjugations between both rings prevents the degradation process from becoming a single step. Lei et al. [44] carried out a study on the thermal degradation of woody lignin and highlighted the role of the biphenyl group in this process. The authors concluded that homolytic cleavage of the biphenyl bond is the most difficult process occurring at higher temperatures.

Since the synthesized ionenes are proposed as SS-PCM in electronic devices where the temperature does not exceed 100 °C, the thermal results obtained indicate that the samples will maintain their structural integrity without problems associated with the degradation process, which would cause a decrease in their performance.

Regarding the residues at 800 °C (R), the ionenes with the biphenyl unit in the main chain (derivatives of BPDA-API) were wholly degraded, but this did not happen in the rest of the samples. This result could be explained by the p-staking interactions that would originate in the ionenes derived from 1*H*,3*H*-benzo [1,2-*c*:4,5-*c*’]difuran-1,3,5,7-tetraone (PMDA), whose highly flat structure would favor the formation of such interchain interactions preventing the total degradation of the sample ([PMDA-API-*p*Xy] [NTf_2_] and [PMDA-API-C_3_] [NTf_2_]). However, regarding the result involving [ODPA-API-*p*Xy] [NTf_2_] and [ODPA-API-C_3_] [NTf_2_] (ionenes derived from 5,5′-oxybis(isobenzofuran-1,3-dione), an anomalous situation is verified, since the presence of the ether function should lead to a higher degradation against [BPDA-API-*p*Xy] [NTf_2_], for example. Further studies should be conducted to clarify in detail the causes of this fact.

Thermal analysis of the samples also involved the development of DSC analysis, in which T_g_ values were retrieved from the heating and cooling curves. The results are summarized in Table 3.

The highest T_g_ values correspond to [PMDA-API-*p*Xy] [NTf_2_], [ODPA-API-*p*Xy] [NTf_2_] and [BPDA-API-*p*Xy] [NTf_2_], probably due to their high aromatic content in the repetitive unit since they are derivatives of 1,4-bis(bromomethyl)benzene, while the other ionenes evidence aliphatic chains of 3, 6 and 12 carbon atoms in that portion. The aromatic fragment causes a more significant restriction to the segmental movement into the polymer chains. The lowest T_g_ value was shown by the ionene obtained from 1*H*,3*H*-benzo[1,2-*c*:4,5-*c*’]difuran-1,3,5,7-tetraone ([PMDA-API-*p*Xy] [NTf_2_]), a result consistent with the smaller aromatic section of the chain compared to the samples having the diaryl ether ([ODPA-API-*p*Xy] [NTf_2_]) and biphenyl ([BPDA-API-*p*Xy] [NTf_2_]) moieties in their structure. Between [ODPA-API-*p*Xy] [NTf_2_] and [BPDA-API-*p*Xy] [NTf_2_], the first showed a lower Tg because the oxyether group can only alternatively conjugate to a single ring at a time, increasing the degrees of freedom of the unconjugated ring. [BPDA-API-*p*Xy] [NTf_2_] showed the highest value due to the higher stiffness of this aromatic section, which is only able to exhibit bending motions because the phenyl rings of the biphenyl system are not in the same plane.

The ionenes derived from the 1,3-dibromopropane monomer ([PMDA-API-C_3_] [NTf_2_], [ODPA-API-C_3_] [NTf_2_] and [BPDA-API-C_3_] [NTf_2_]) showed the lowest T_g_ values, thanks to the flexibility that the aliphatic chain (C3) gives them. For this series, the same pattern was observed with respect to the dianhydride precursor mentioned above. Table 3 also shows that at the same heating and cooling rate, the T_g_ values obtained present a greater difference in the case of [ODPA-API-*p*Xy] [NTf_2_], [BPDA-API-*p*Xy] and [BPDA-API-C_3_] [NTf_2_], while for [PMDA-API-*p*Xy] [NTf_2_], the values recorded are highly coincident. This implies that the [ODPA-API-*p*Xy] [NTf_2_], [BPDA-API-*p*Xy] and [BPDA-API-C_3_] [NTf_2_] ionene chains, under the conditions of the analysis, take longer to reach equilibrium after a heating process. Ionenes with T_g_ lower than 40 °C (heating curve) showed no T_g_ values during the cooling processes. This phenomenon is believed to be due to the cooling rate leaving the ionenes in a metastable, non-hardened condition. It could be observed that ionenes with this property could self-heal against deformations caused by a cutting or penetration force. This is due to the reduced ordering of the polymer chains and the presence of ionic bonds.

Knowing that the highest Tg value found in the ionenes corresponded to those containing the biphenyl moiety, the synthesis of [BPDA-API-C_6_] [NTf_2_] and [BPDA-API-C_12_] [NTf_2_] was carried out, thus testing the effect of increasing the aliphatic chain length. As expected, T_g_ decreased as the aliphatic chain became longer (Table 3, Figure S13). This difference was much more significant when the propyl (C_3_) chain of [BPDA-API-C_3_] [NTf_2_] was replaced by a hexyl (C_6_) chain in [BPDA-API-C_6_] [NTf_2_] (∆ = 26.3 °C) than when the C_6_ chain was replaced by a C_12_ chain ([BPDA-API-C_6_] [NTf_2_] vs. [BPDA-API-C_12_] [NTf_2_]) (∆ = 4.1 °C). Despite the difference in the number of carbon atoms from one to the other, the thermal behavior is the same. With these results, it is possible to infer that for this type of polymer, the contribution of the aliphatic fragment on T_g_ will be less significant from chains with six or more carbon atoms.

### 3.4. Self-Healing Behavior

**Figure 6:**
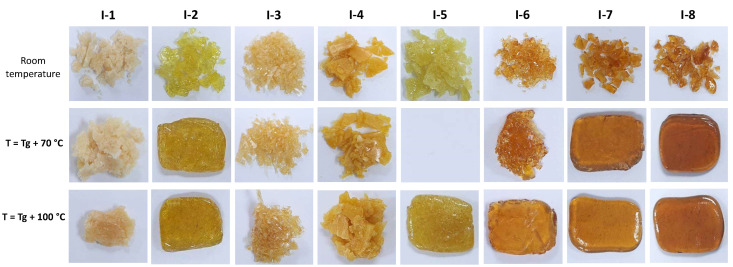
Appearance of ionenes at room temperature and 70 °C and 100 °C above T_g_. **I-1:** [PMDA-API-*p*Xy] [NTf_2_]; **I-2:** [ODPA-API-*p*Xy] [NTf_2_]; **I-3**: [BPDA-API-*p*Xy] [NTf_2_]; **I-4:** [PMDA-API-C_3_] [NTf_2_]; **I-5:** [ODPA-API-C_3_] [NTf_2_]; **I-6:** [BPDA-API-C_3_] [NTf_2_]; **I-7:** [BPDA-API-C_6_] [NTf_2_]; **I-8:** [BPDA-API-C_12_] [NTf_2_].

The self-healing behavior of ionenes was preliminarily studied. Firstly, the ionenes were heated up to 70 °C and 100 °C above T_g_ in order to make rectangular pieces (Figure 6). Ionenes [PMDA-API-*p*Xy] [NTf_2_], [BPDA-API-*p*Xy] [NTf_2_] and [PMDA-API-C_3_] [NTf_2_] were not able to be thermomolded at those temperatures, and ionene [BPDA-API-C_3_] [NTf_2_] was only thermomolded at 100 °C above its Tg (155 °C).

**Figure 7:**
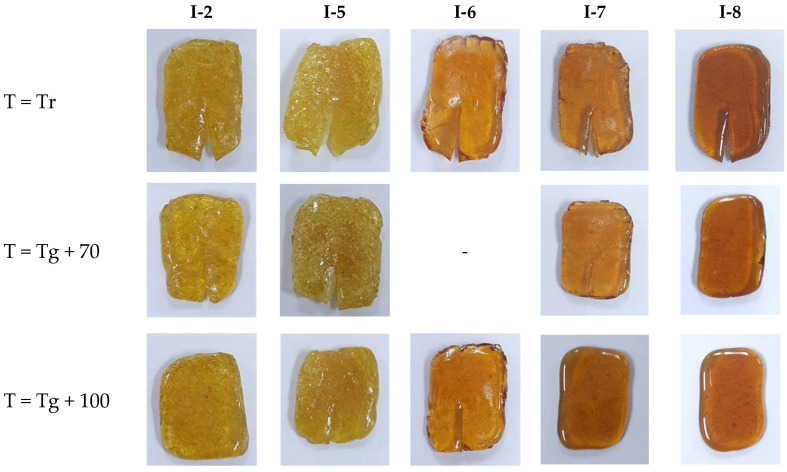
Ionene self-healing test. **I-2:** [ODPA-API-*p*Xy] [NTf_2_]; **I-5:** [ODPA-API-C_3_] [NTf_2_]; **I-6:** [BPDA-API-C_3_] [NTf_2_]; **I-7:** [BPDA-API-C_6_] [NTf_2_]; **I-8:** [BPDA-API-C_12_] [NTf_2_].

Rectangular pieces of ionenes [ODPA-API-*p*Xy] [NTf_2_], [ODPA-API-C_3_] [NTf_2_], [BPDA-API-C_3_] [NTf_2_], [BPDA-API-C_6_] [NTf_2_] and [BPDA-API-C_12_] [NTf_2_] were cut at room temperature and placed on a Teflon plate at T = T_g_ + 70 °C and T = T_g_ + 100 °C, except for I-6, which was only evaluated at T = T_g_ + 100 °C. After 2 min, ionene I-8 was entirely self-healed at T = T_g_ + 70 °C. Ionenes [ODPA-API-*p*Xy] [NTf_2_] and [BPDA-API-C_6_] [NTf_2_] were self-healed after 10 min at T = Tg + 100 °C, while I-5 and I-6 were almost self-healed but the cut marks were still visible (Figure 7).

As can be seen, no deformation, draining or color change was observed at the working temperature. These results could be attributed to the ionic intra- and intermolecular forces and charge transfer complexes of the imide groups [33,45,46]. In the case of the ionenes tested, a greater repair efficiency was observed for [ODPA-API-*p*Xy] [NTf_2_], [ODPA-API-C_3_] [NTf_2_], [BPDA-API-C_6_] [NTf_2_] and [BPDA-API-C_12_] [NTf_2_]. These four materials have the most flexible molecular structures. The flexibility of [ODPA-API-*p*Xy] [NTf_2_] and [ODPA-API-C_3_] [NTf_2_] is provided by the oxyether group and, in the case of [BPDA-API-C_6_] [NTf_2_] and [BPDA-API-C_12_] [NTf_2_], the aliphatic chains of six and twelve carbon atoms, respectively. In both cases, the softness of the chains favors the viscoelasticity of the materials and the mobility of the ions within the material, allowing the reconstitution of the bonds and the self-healing observed [47,48].

### 3.5. Hydrophilicity

One of the most important properties of the materials used in electronic devices is their ability to absorb water. It is known how this substance causes short circuits and oxidation, or promotes sulfation of devices. For this reason, it was decided to determine the hydrophilicity of the synthesized polymers using the contact angle technique from films prepared from each of them.

According to the contact angle values of the ionene films (Table 4), [BPDA-API-*p*Xy] [NTf_2_] exhibited the highest contact angle with water (97.3°) and the lowest with diiodomethane (40.1°), being more hydrophobic than the rest of the ionenes. The presence of CF_3_ groups in the counterion should increase the hydrophobicity despite the fact of ionic groups. Therefore, higher contact angle values were expected. Based on these results, most of the CF_3_ groups might have been found oriented towards the interior of the polymeric membrane, leaving only traces on the surface. It is possible to support this conclusion with the results obtained from Cardiano et al. [49], who reported the synthesis and hydrophobic properties of three aliphatic ionic polymers. The structural difference of their polymers lay in the fluorine content and the length of the hydrocarbon chain in the anion. According to them, the polymer with NTf_2_ as counterion had a contact angle of 76.4°. The XPS test results obtained by these researchers confirmed that the hydrophobic CF_3_ groups were oriented towards the interior of the membrane.

Table 4 also shows the total surface energy values, which were estimated by calculations performed by the Owens–Wendt–Rabel–Kaelble method using water and diiodomethane as solvent [46].

Ionenes with high aromatic content ([PMDA-API-*p*Xy] [NTf_2_], [ODPA-API-*p*Xy] [NTf_2_] and [BPDA-API-*p*Xy] [NTf_2_]) showed higher hydrophobicity than their counterparts. In these samples, the higher stiffness and flatness of the chain favor a more efficient packing, which hinders the penetration of water molecules, and therefore the wettability is reduced. However, as the chain or aliphatic section increases, the water repulsion increases, as observed in the progression of the values obtained for ionenes [BPDA-API-C_3_] [NTf_2_], [BPDA-API-C_6_] [NTf_2_] and [BPDA-API-C_12_] [NTf_2_]. Both results are corroborated by total surface energy calculations, where it is observed how the contribution of the polar and dispersive sections is consistent with both the values of the contact angle of the water droplets and the structure of the analyzed ionenes. Photographs of the contact angle measurements are available in Figure S14.

## 4. Conclusions

In the present work, eight ionic polymers were synthesized by Menshutkin reactions and a new and efficient methodology to carry out ion exchange was explored. The structure of ionenes was corroborated by ATR-IR, ^1^H NMR, ^13^C NMR and ^19^F NMR spectroscopy, and the solubility test performed at room temperature evidenced high solubility in aprotic polar solvents. All ionenes demonstrated excellent thermal resistance with T_10%_ values above 420 °C and T_g_ values on the heating curve ranging from 26 °C to 83 °C. The self-healing behavior of the ionenes was explored with promising results. Ionene containing biphenyl and C_12_ moieties in its repeating unit ([BPDA-API-C_12_] [NTf_2_]) was selected to demonstrate how the self-healing process occurs a short time after appropriate thermal treatment. Contact angle tests were conducted on the films prepared with the synthesized polymers. Only one ionene ([BPDA-API-*p*Xy] [NTf_2_]) presented a contact angle value greater than 90°, a criterion that makes it conceptually hydrophobic. However, the rest of the ionenes presented contact angles ranging from 78.2° to 85.8°.

The results obtained allow the inference that the synthesized ionenes possess properties that make them potential candidates for studies as phase change materials in building heating and cooling systems, and for electronic applications, among others. Additionally, the fact that they undergo a solid–solid phase change makes them especially attractive for various applications where the main challenge is to avoid leakage of the material by runoff when changing to the liquid state.

## Figures and Tables

**Table 1:** Solubility behavior of PILs in different solvents at room temperature.

	I-1	I-2	I-3	I-4	I-5	I-6	I-7	I-8
Water	−	−	−	−	−	−	−	−
DMSO	+	+	+	+	+	+	+	+
DMF	+	+	+	+	+	+	+	+
Acetonitrile	+	+	+	+	+	+	+	+
Ethanol	−	−	−	−	−	−	−	−
Methanol	−	−	−	−	−	−	−	−
Acetone	+	+	+	+	+	+	+	+
Ethyl acetate	−	−	−	−	−	−	−	−
Chloroform	−	−	−	−	−	−	−	−
Ethyl ether	−	−	−	−	−	−	−	−

+: Soluble, −: Insoluble even at the boiling point of the solvent. **I-1**: [PMDA-API-*p*Xy] [NTf_2_]; **I-2**: [ODPA-API-*p*Xy] [NTf_2_]; **I-3**: [BPDA-API-*p*Xy] [NTf_2_]; **I-4**: [PMDA-API-C_3_] [NTf_2_]; **I-5**: [ODPA-API-C_3_] [NTf_2_]; **I-6**: [BPDA-API-C_3_] [NTf_2_]; **I-7**: [BPDA-API-C_6_] [NTf_2_]; **I-8**: [BPDA-API-C_12_] [NTf_2_].

**Table 2:** Results of the TGA analysis (under nitrogen atmosphere) of the ionenes synthesized.

	T_onset_ (°C) ^a^	T_5%_ (°C) ^b^	T_10%_ (°C) ^c^	T_d1_ (°C) ^c^	T_d2_ (°C) ^d^	R (%) ^e^
[PMDA-API-*p*Xy] [NTf_2_]	420	421	430	450	-	33
[ODPA-API-*p*Xy] [NTf_2_]	433	430	439	448	-	37
[BPDA-API-*p*Xy] [NTf_2_]	429	392	440	476	597	0
[PMDA-API-C_3_] [NTf_2_]	432	400	434	464	-	0
[ODPA-API-C_3_] [NTf_2_]	441	361	427	461	-	26
[BPDA-API-C_3_] [NTf_2_]	433	436	447	472	584	1
[BPDA-API-C_6_] [NTf_2_]	432	400	434	480	577	0
[BPDA-API-C_12_] [NTf_2_]	421	394	431	484	560	0

^a^ Temperature at which the polymer degradation begins. ^b^ Temperature at which 5% and 10% weight is lost, respectively. ^c^ Temperature at which material is degraded in one and/or two stages, respectively. ^d^ Percentage of residual material. ^e^ Char yield.

**Table 3:** DSC assays (under nitrogen atmosphere) for ionenes.

	Heating Curve	Cooling Curve	
	T_g_ (°C)	T_g_ (°C)	∆T_g_ (°C)
[PMDA-API-*p*Xy] [NTf_2_]	51.7	51.3	0.4
[ODPA-API-*p*Xy] [NTf_2_]	70.6	64.0	6.6
[BPDA-API-*p*Xy] [NTf_2_]	83.3	78.8	4.5
[PMDA-API-C_3_] [NTf_2_]	26.0	-	-
[ODPA-API-C_3_] [NTf_2_]	38.4	-	-
[BPDA-API-C_3_] [NTf_2_]	54.5	52.0	2.5
[BPDA-API-C_6_] [NTf_2_]	28.2	-	-
[BPDA-API-C_12_] [NTf_2_]	24.1	-	-

Tg: Glass transition temperature. Heating/cooling rate = 10 °C/min.

**Table 4:** Contact angle values, total surface energy, and polar and non-polar components for ionenes.

	Average Contact Angle		γ_S_ ^a^ (mN/m)	γ_S_D ^b^ (mN/m)	γ_S_P ^c^ (mN/m)
	Water	Diiodomethane			
[PMDA-API-*p*Xy] [NTf_2_]	80.1	60.8	30.9	22.2	8.6
[ODPA-API-*p*Xy] [NTf_2_]	85.8	76.3	23.4	14.3	9.1
[BPDA-API-*p*Xy] [NTf_2_]	97.3	40.1	39.9	39.7	0.3
[PMDA-API-C_3_] [NTf_2_]	79.1	51.5	34.9	27.7	7.2
[ODPA-API-C_3_] [NTf_2_]	80.9	58.5	31.5	23.8	7.7
[BPDA-API-C_3_] [NTf_2_]	78.2	47.4	36.9	29.9	6.9
[BPDA-API-C_6_] [NTf_2_]	82.4	49.5	34.9	29.8	5.2
[BPDA-API-C_12_] [NTf_2_]	85.5	49.0	34.7	30.9	3.8

^a^ Total surface energy; ^b^ dispersive or non-polar component (γ_S_D); ^c^ polar component (γ_S_P).

## Data Availability

Data are contained within the article.

## Supplementary Materials

The following supporting information can be downloaded at: https://www.mdpi.com/article/10.3390/polym15224460/s1, Figure S1: A. Experimental setup for the monomer synthesis. B. Experimental setup for the ionene synthesis; Figure S2: IR and ^1^H, ^13^C and Dept 135° NMR spectra of PMDA-API; Figure S3: IR and ^1^H, ^13^C and Dept 135° NMR spectra of ODPA-API; Figure S4: IR and ^1^H, ^13^C and Dept 135° NMR spectra of BPDA-API; Figure S5: IR and ^1^H, ^13^C, Dept 135° and ^19^F NMR spectra of ionene [PMDA-API-pXy] [NTf_2_]; Figure S6: IR and ^1^H, ^13^C, Dept 135° and ^19^F NMR spectra of ionene [ODPA-API-pXy] [NTf_2_]; Figure S7: IR and ^1^H, ^13^C, Dept 135° and ^19^F NMR spectra of ionene [BPDA-API-pXy] [NTf_2_]; Figure S8: IR and ^1^H, ^13^C, Dept 135° and ^19^F NMR spectra of ionene [PMDA-API-C_3_] [NTf_2_]; Figure S9: IR and ^1^H, ^13^C, Dept 135° and ^19^F NMR spectra of ionene [ODPA-API-C_3_] [NTf_2_]; Figure S10: IR and ^1^H, ^13^C, Dept 135° and ^19^F NMR spectra of ionene [BPDA-API-C_3_] [NTf_2_]; Figure S11: IR and ^1^H, ^13^C, Dept 135° and ^19^F NMR spectra of ionene [BPDA-API-C_6_] [NTf_2_]; Figure S12: IR and ^1^H, ^13^C, Dept 135° and ^19^F NMR spectra of ionene [BPDA-API-C_12_] [NTf_2_]; Figure S13: Heating curves of the DSC assays of the synthesized ionenes; Figure S14: Some contact angle measurements for the synthesized ionenes.
