# Supplementary material for: Ionenes as Potential Phase Change Materials with Self-Healing Behavior
Source: Polymers (Basel). 2023 Nov 19;15(22):4460. doi: 10.3390/polym15224460 (PMC10674965; doi:10.3390/polym15224460)
Supplement: Supplementary file 1 [file polymers-15-04460-s001.zip › polymers-2708296-supplementary.pdf]

Supplementary Materials

Ionenes as Potential Phase Change Materials with Self-Healing Behavior

Carolina Arriaza-Echanes [1] , María V. Velázquez-Tundidor [2], Alejandro Angel-López [1], Ángel Norambuena [1,3], Francisco E. Palay [1] , Claudio A. Terraza [2,4] , Alain Tundidor-Camba [2,4] , Pablo A. Ortiz [1,5,6,*] and
Deysma Coll [1,6,7,*]

[1] Vicerrectoría de Investigación, Universidad Mayor, Camino la Pirámide 5750, Santiago 8580745, Chile; angel.norambuena@mayor.cl (Á.N.); francisco.palay@mayor.cl (F.E.P.)
[2] Research Laboratory for Organic Polymers (RLOP), Department of Organic Chemistry, Pontificia Universidad Católica de Chile, Santiago 7820436, Chile
[3] Instituto de Investigaciones y Control del Ejército de Chile (IDIC), Santiago 8370899, Chile
[4] UC Energy Research Center, Pontificia Universidad Católica de Chile, Santiago 7820436, Chile
[5] Escuela de Ingeniería en Medio Ambiente y Sustentabilidad, Facultad de Ciencias, Ingeniería y Tecnología, Universidad Mayor, Camino La Pirámide 5750, Santiago 8580745, Chile
[6] Centro de Nanotecnología Aplicada, Facultad de Ciencias, Ingeniería y Tecnología, Universidad Mayor, Camino La Pirámide 5750, Santiago 8580745, Chile
[7] Núcleo de Química y Bioquímica, Facultad de Ciencias, Ingeniería y Tecnología, Universidad Mayor, Camino La Pirámide 5750, Santiago 8580745, Chile
* Correspondence: pablo.ortiz@umayor.cl (P.A.O.); deysma.coll@umayor.cl (D.C.)

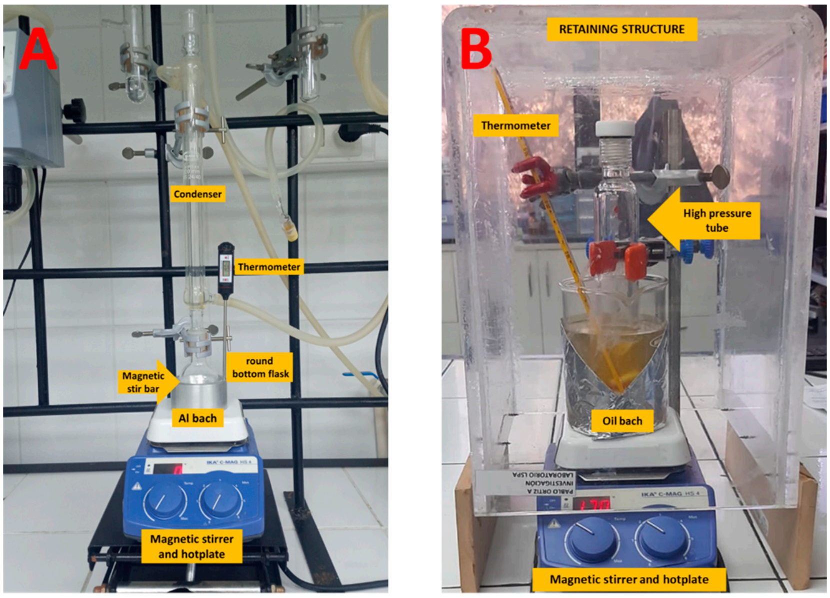

Figure S1. A. Experimental setup for the monomers synthesis. **B.** Experimental setup for the ionenes synthesis.

Spectroscopic data

Below are the infrared and nuclear magnetic resonance spectra of both the synthesized monomers and ionenes.

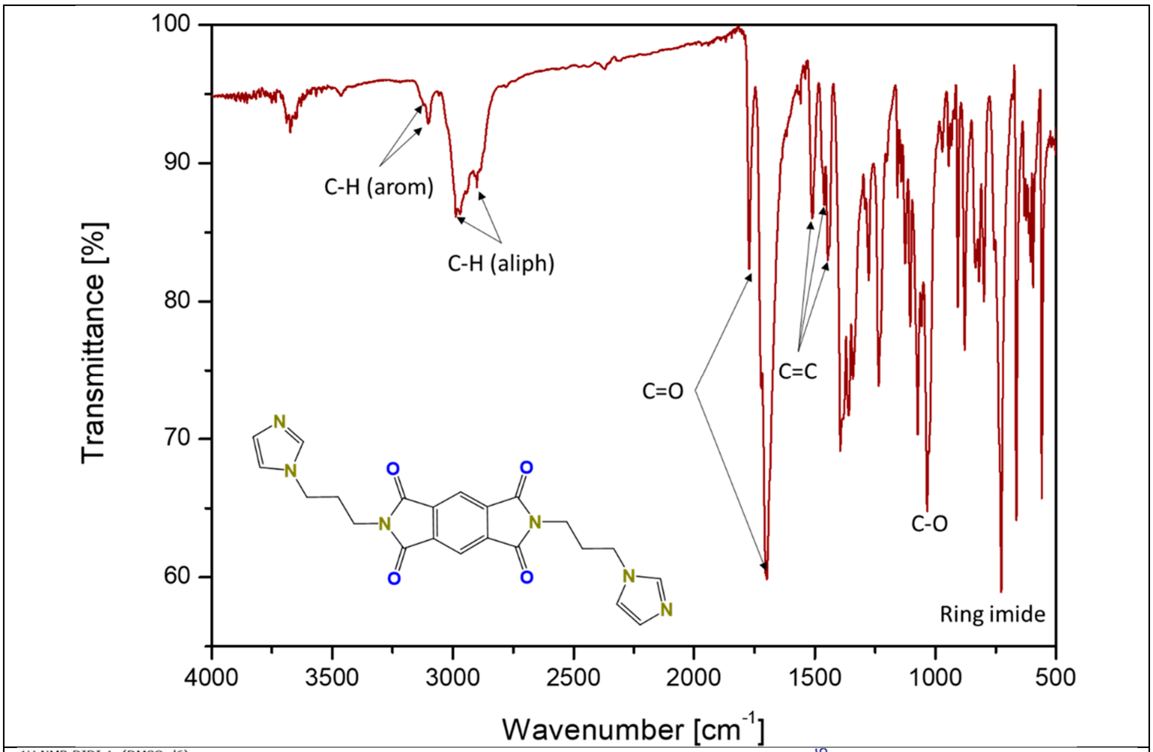
Transmittance [%]
Wavenumber [cm^{-1}]
100
90
80
70
60
4000
3500
3000
2500
2000
1500
1000
500
C-H (arom)
C-H (aliph)
C=O
C=C
C-O
Ring imide
N
O

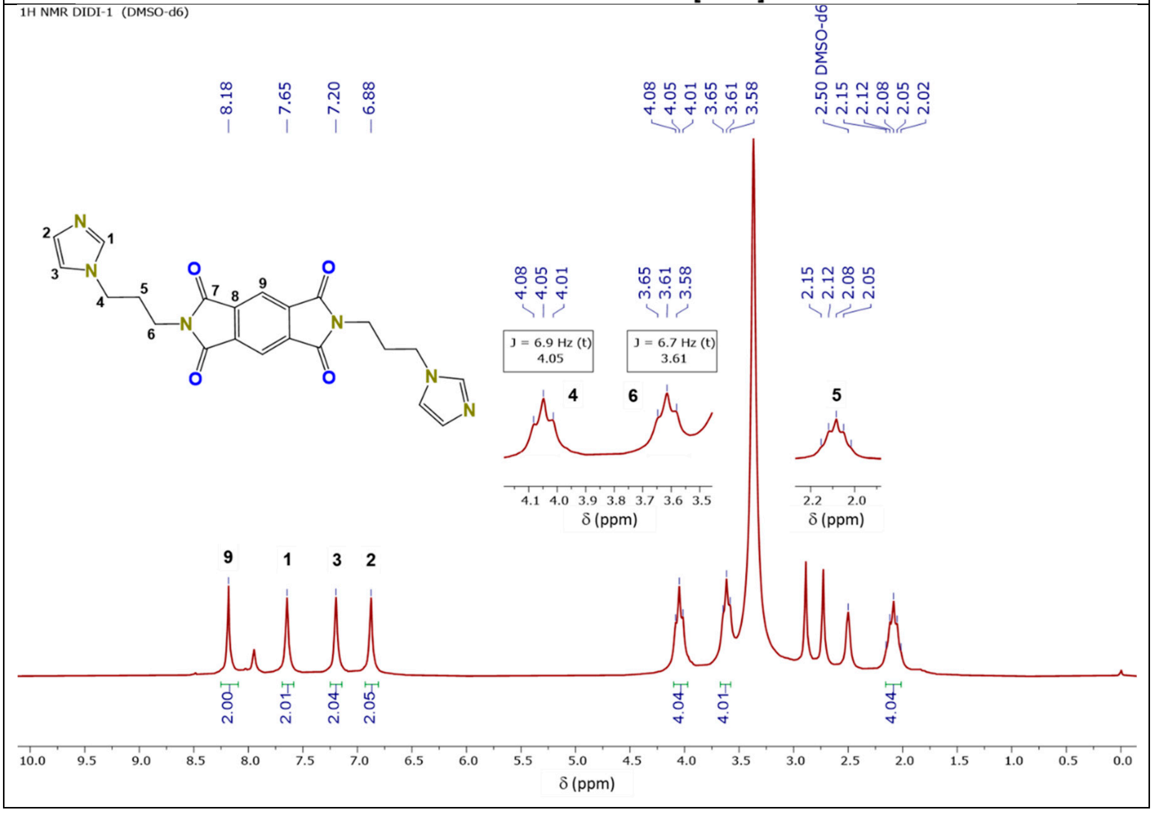
1H NMR DIDI-1 (DMSO-d6)
8.18
7.65
7.20
6.88
4.08
4.05
4.01
3.65
3.61
3.58
2.50 DMSO-d6
2.15
2.12
2.08
2.05
2.02
J = 6.9 Hz (t)
4.05
J = 6.7 Hz (t)
3.61
9
1
3
2
4
6
5
2.00
2.01
2.04
2.05
4.04
4.01
4.04
δ (ppm)

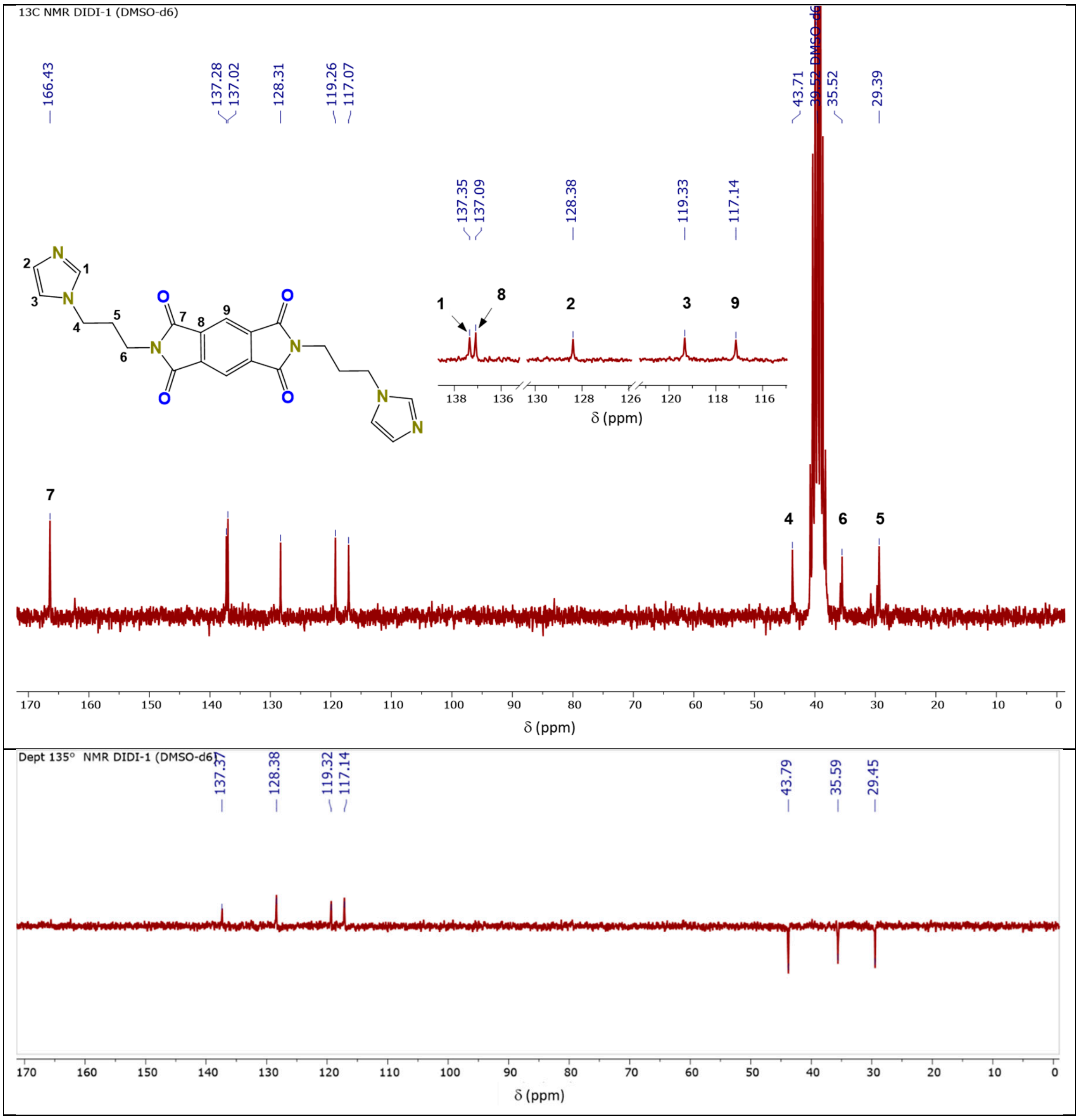

Figure S2. IR and ^{1}H, ^{13}C and Dept 135°NMR spectra of PMDA-API.

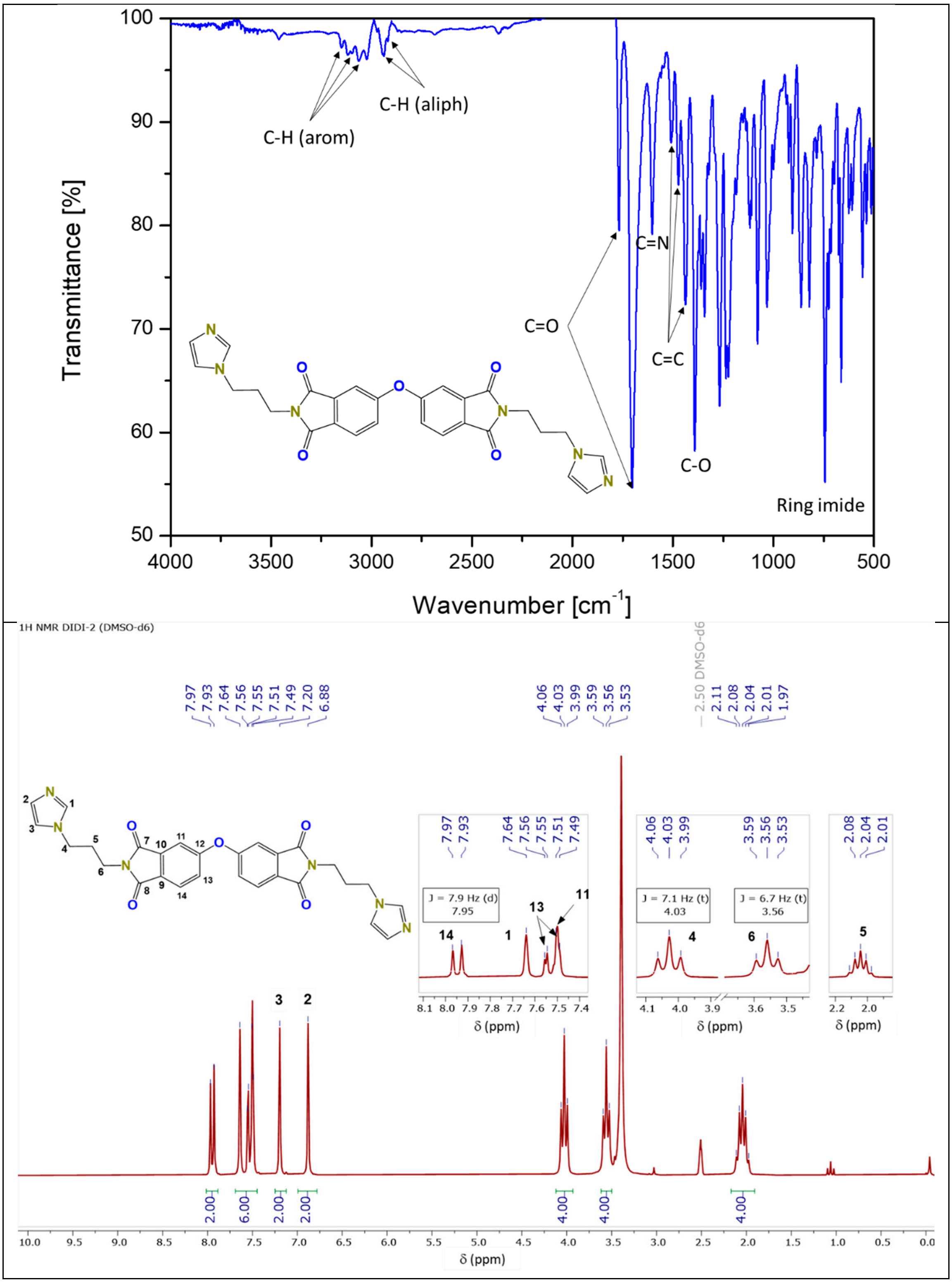
Transmittance [%]
Wavenumber [cm-1]
C-H (arom)
C-H (aliph)
C=O
C=N
C=C
C-O
Ring imide
1H NMR DIDI-2 (DMSO-d6)
2.50 DMSO-d6
J = 7.9 Hz (d) 7.95
J = 7.1 Hz (t) 4.03
J = 6.7 Hz (t) 3.56
δ (ppm)

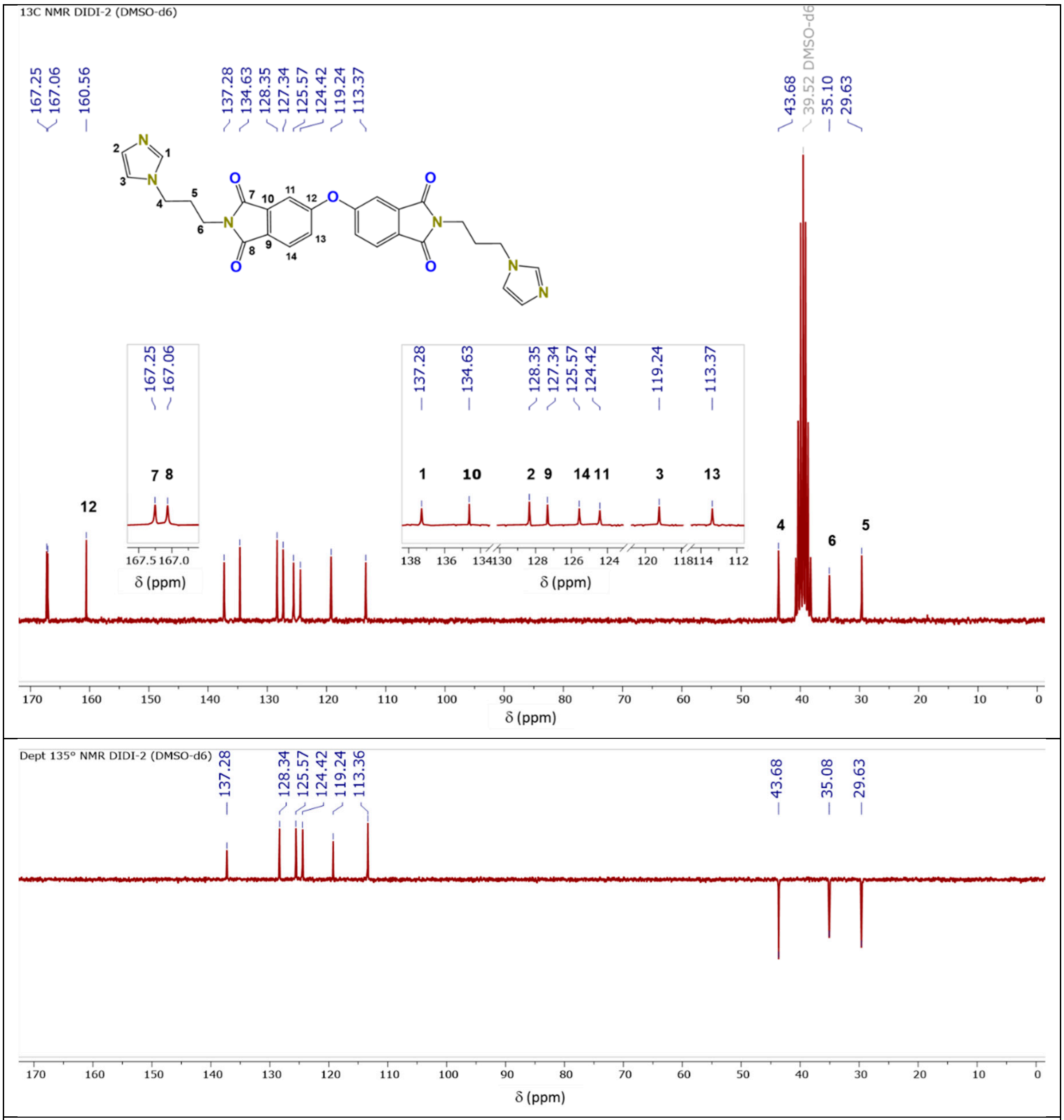

Figure S3. IR and ^{1}H, ^{13}C and Dept 135° NMR spectra of ODPA-API .

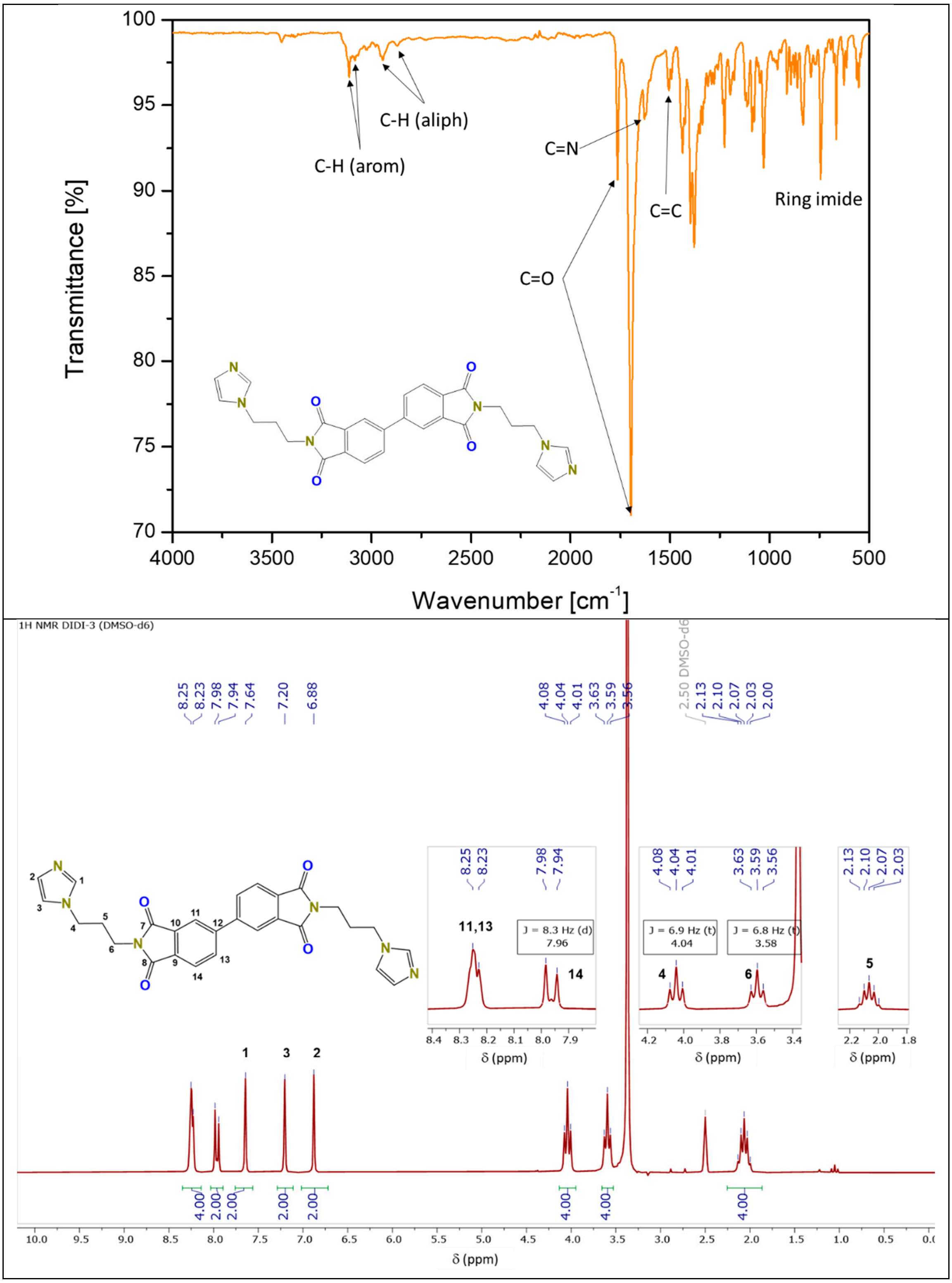

Transmittance [%]
Wavenumber [cm-1]
C-H (arom)
C-H (aliph)
C=N
C=O
C=C
Ring imide
1H NMR DIDI-3 (DMSO-d6)
8.25 8.23 7.98 7.94 7.64 7.20 6.88
4.08 4.04 4.01 3.63 3.59 3.56
2.50 DMSO-d6
2.13 2.10 2.07 2.03 2.00
11,13
14
J = 8.3 Hz (d) 7.96
J = 6.9 Hz (t) 4.04
J = 6.8 Hz (t) 3.58
4
6
5
1
3
2
4.00 2.00 2.00 2.00 2.00 4.00 4.00 4.00
δ (ppm)

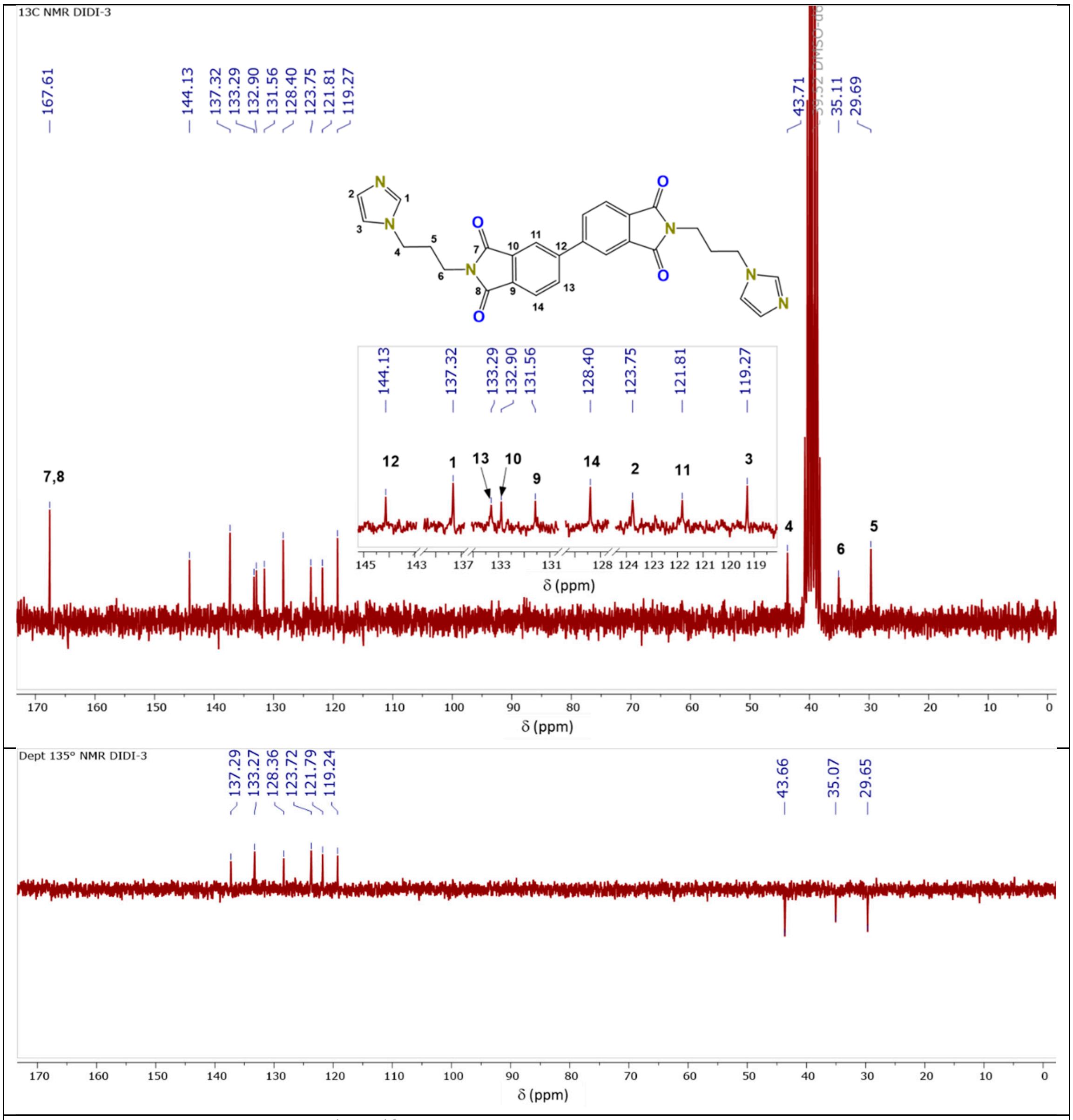

Figure S4. IR and ^{1}H, ^{13}C and Dept 135° NMR spectra of BPDA-API .

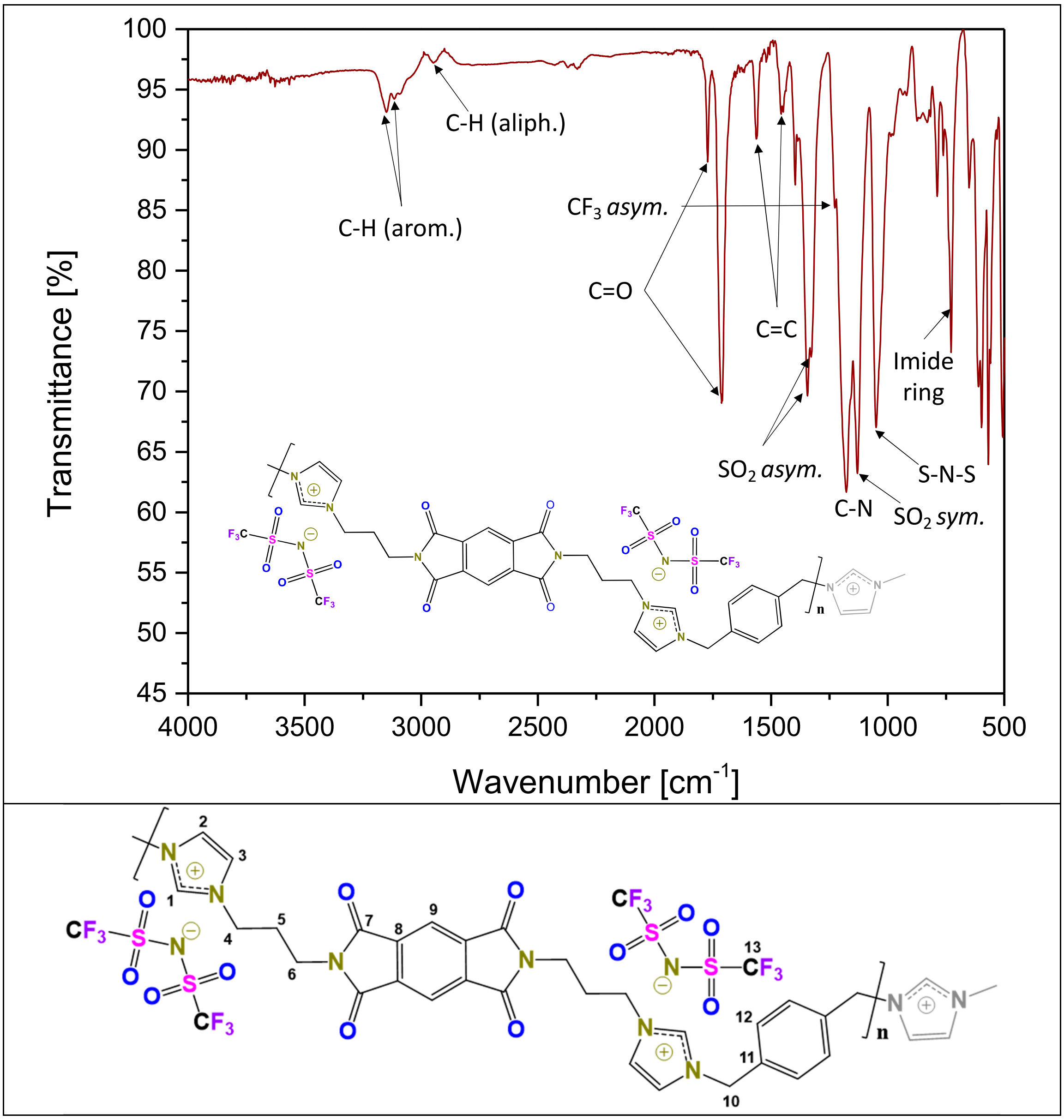

Transmittance [%]
Wavenumber [cm-1]
C-H (arom.)
C-H (aliph.)
CF3 asym.
C=O
C=C
Imide ring
SO2 asym.
C-N
S-N-S
SO2 sym.
n

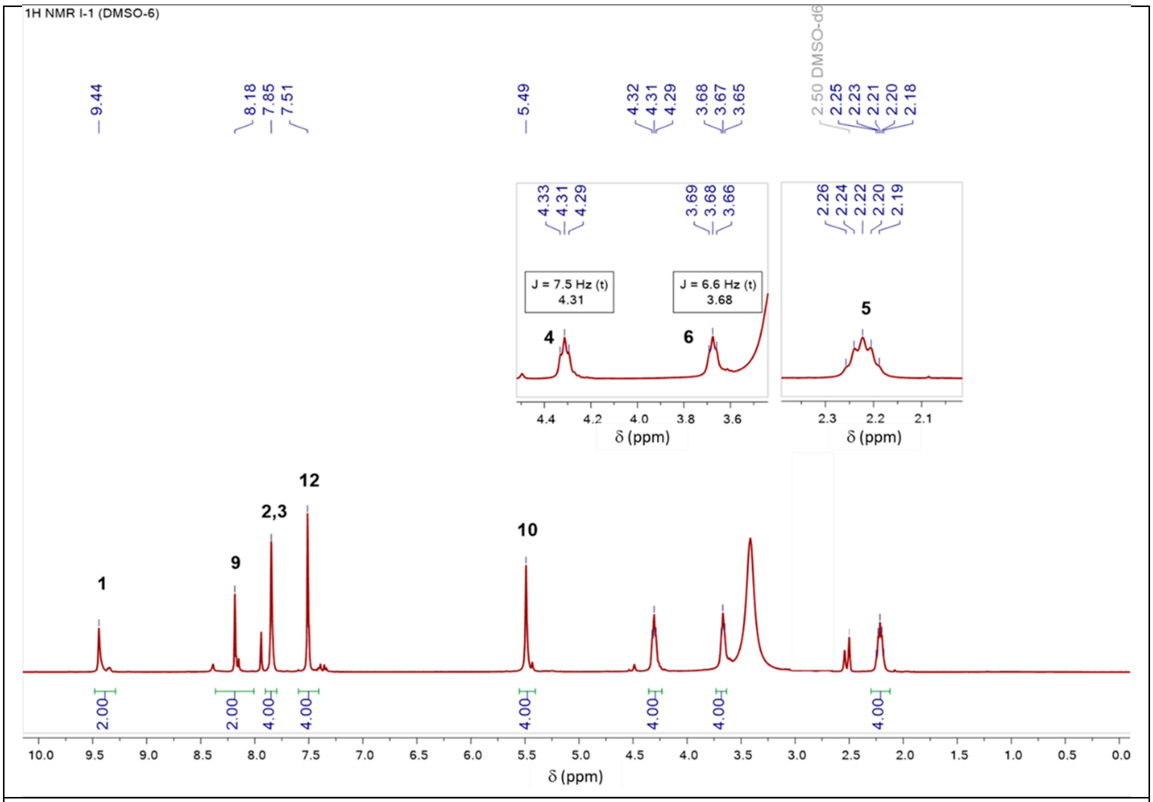
1H NMR I-1 (DMSO-6)
J = 7.5 Hz (t)
4.31
J = 6.6 Hz (t)
3.68
δ (ppm)

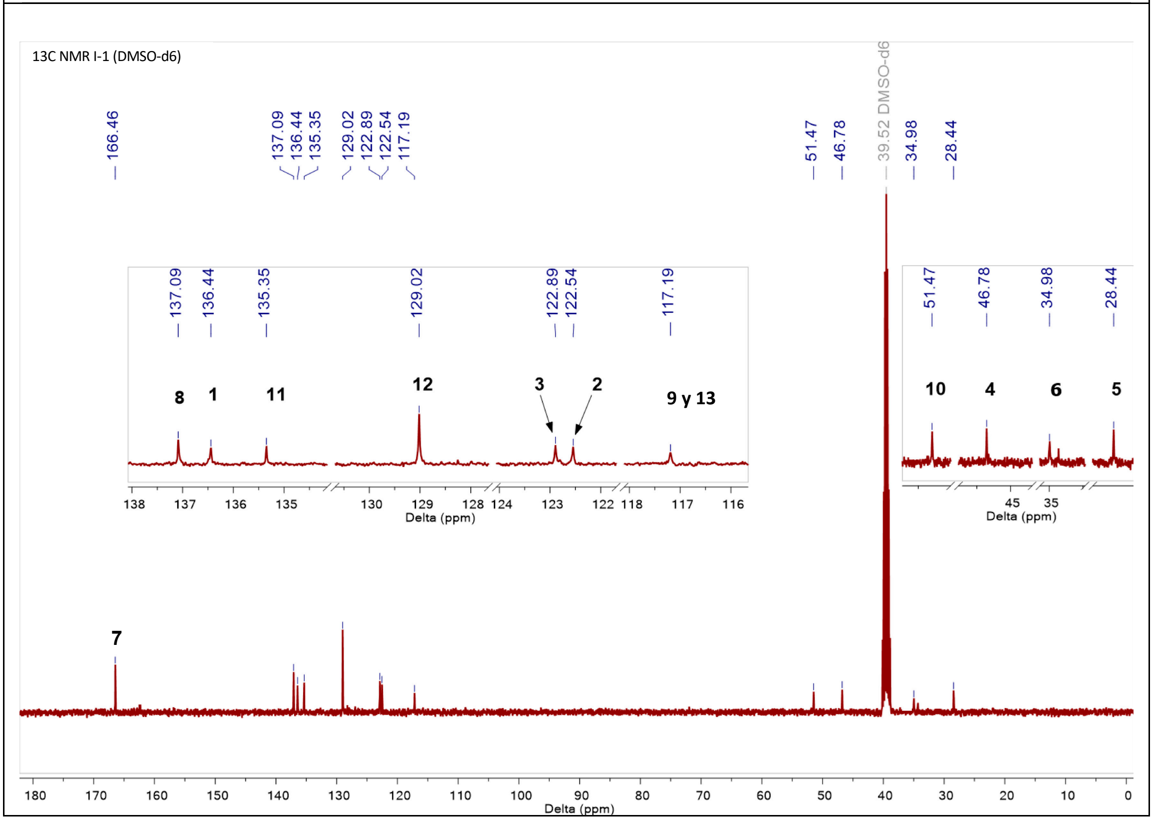
13C NMR I-1 (DMSO-d6)
9 y 13
Delta (ppm)

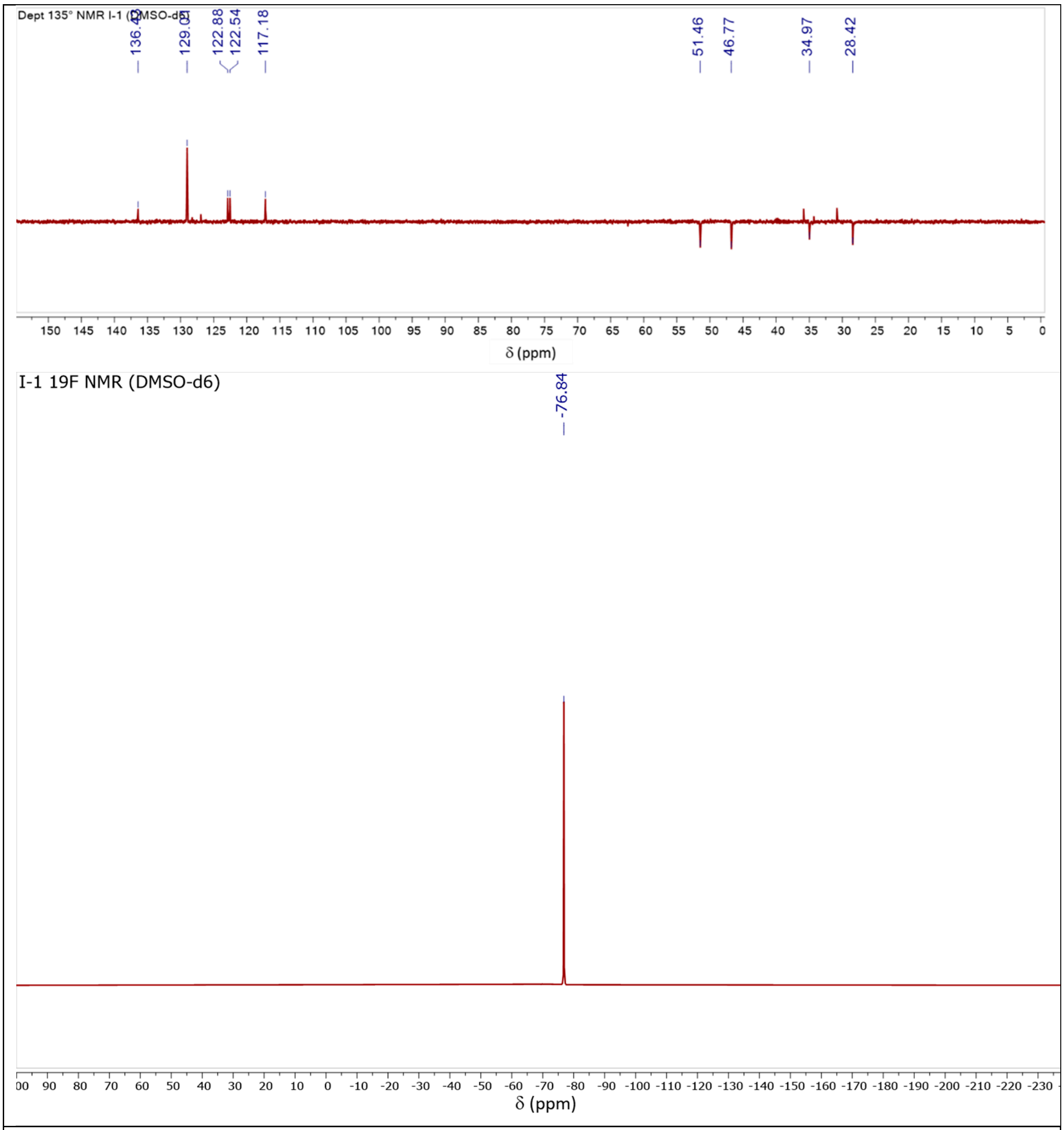

Figure S5. IR and ^{1}H, ^{13}C, Dept 135° and ^{19}F NMR spectra of ionene [PMDA-API-pXy][NTf_2].

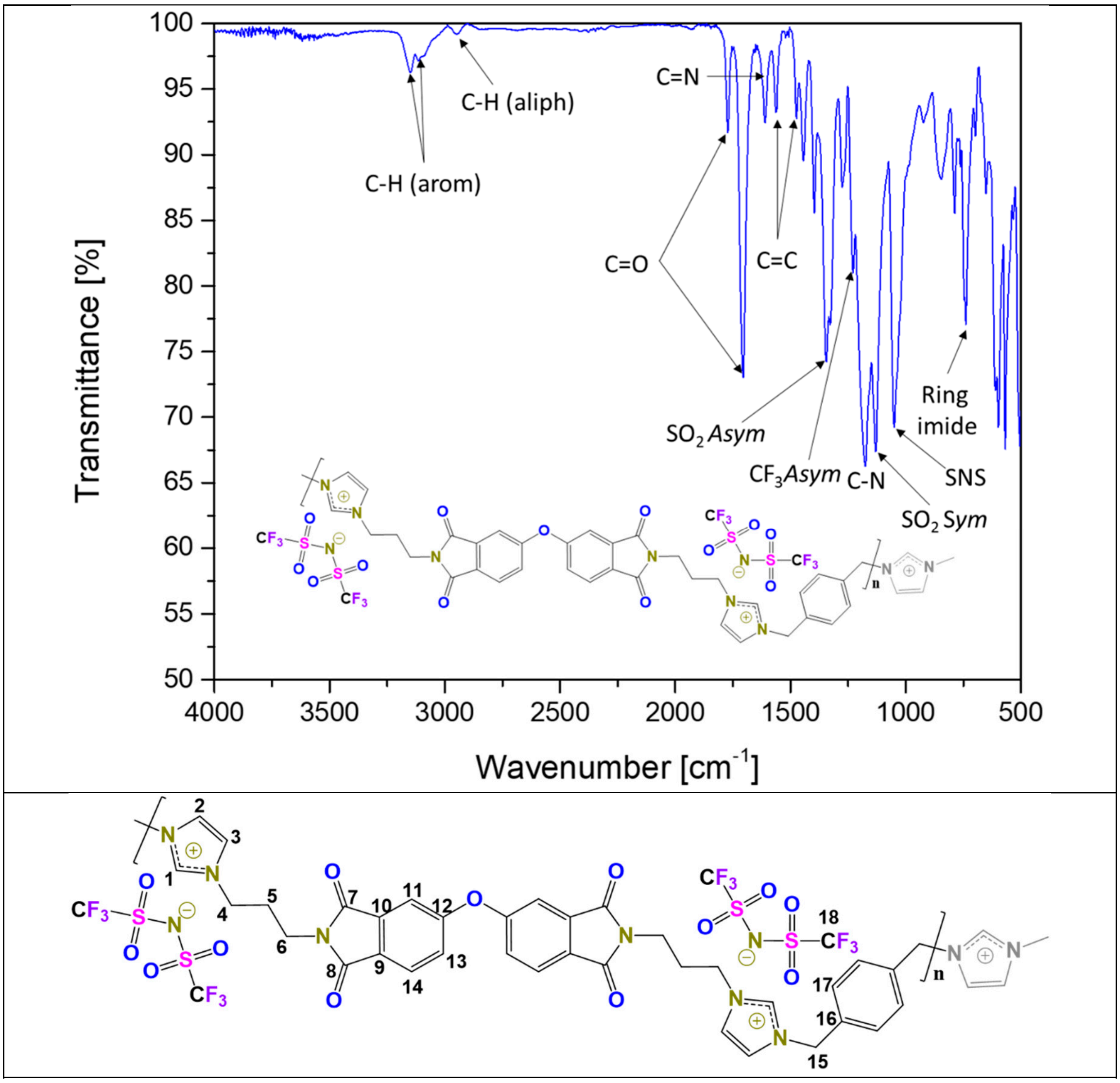

Transmittance [%]
Wavenumber [cm-1]
100
95
90
85
80
75
70
65
60
55
50
4000
3500
3000
2500
2000
1500
1000
500
C-H (arom)
C-H (aliph)
C=N
C=O
C=C
SO2 Asym
CF3Asym
C-N
SO2 Sym
SNS
Ring imide

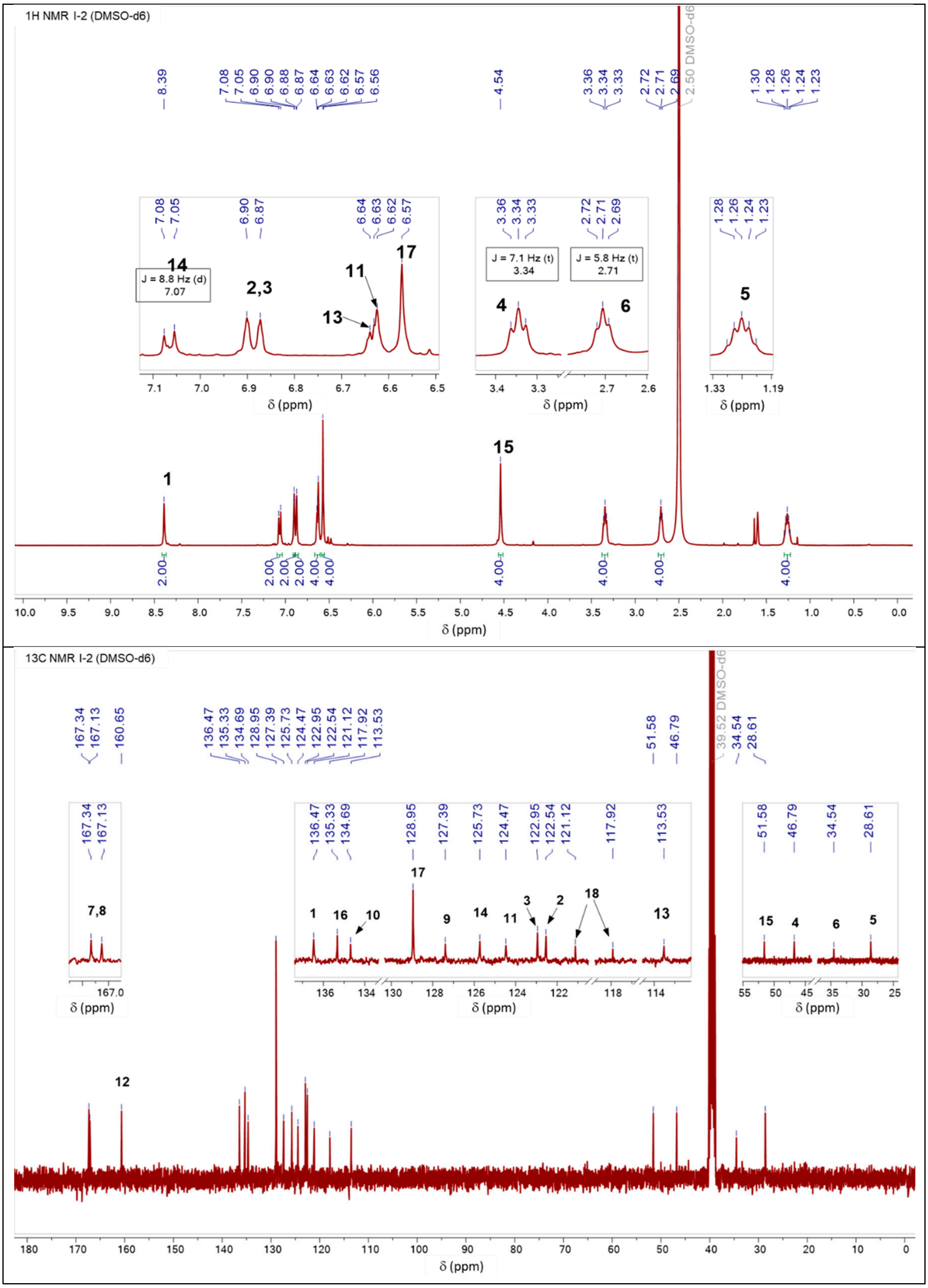
1H NMR I-2 (DMSO-d6)
J = 8.8 Hz (d)
7.07
J = 7.1 Hz (t)
3.34
J = 5.8 Hz (t)
2.71
δ (ppm)
13C NMR I-2 (DMSO-d6)
δ (ppm)

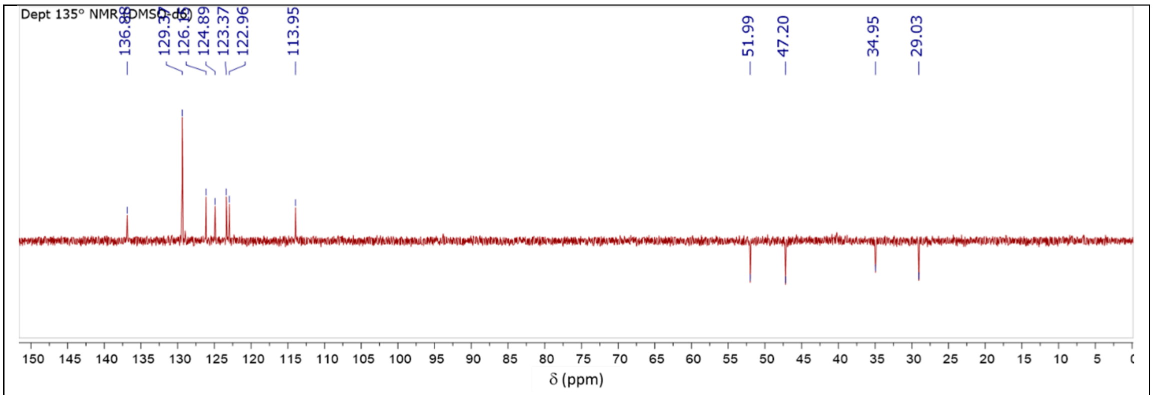

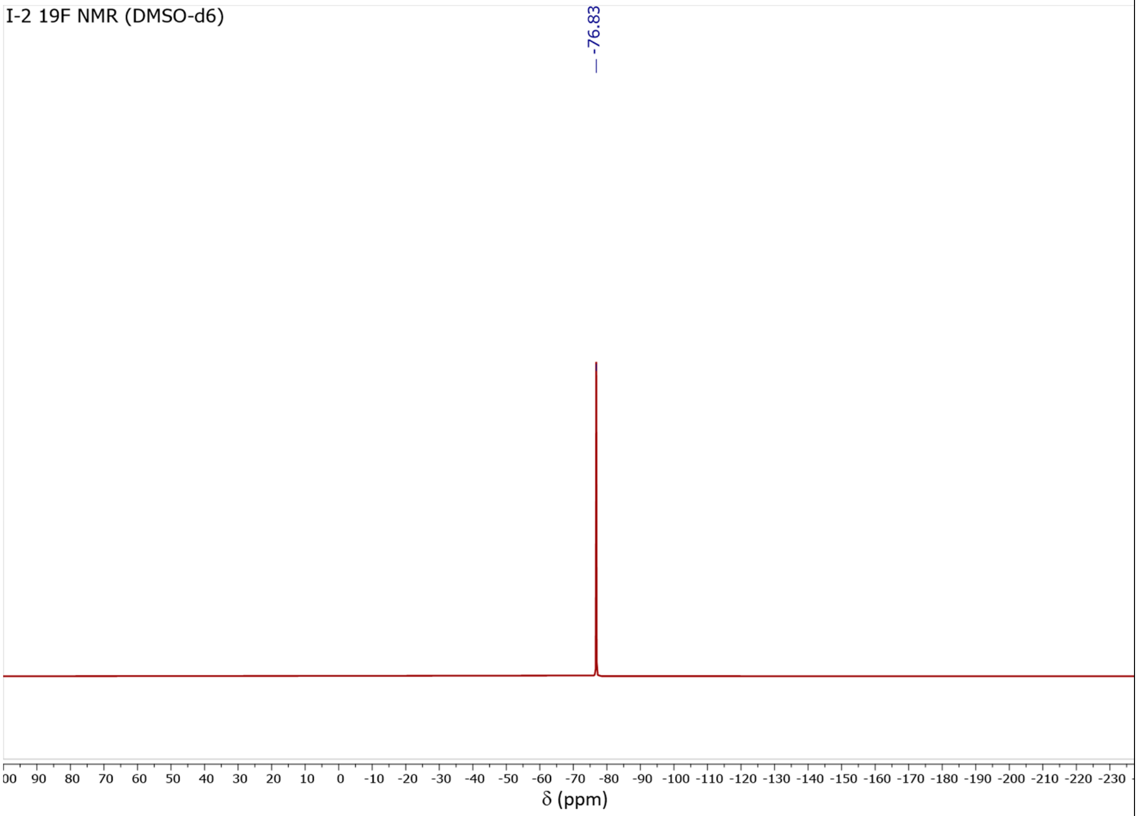

Figure S6. IR and ^{1}H, ^{13}C, Dept 135° and ^{19}F NMR spectra of ionene [ODPA-API-pXy][NTf$_2$].

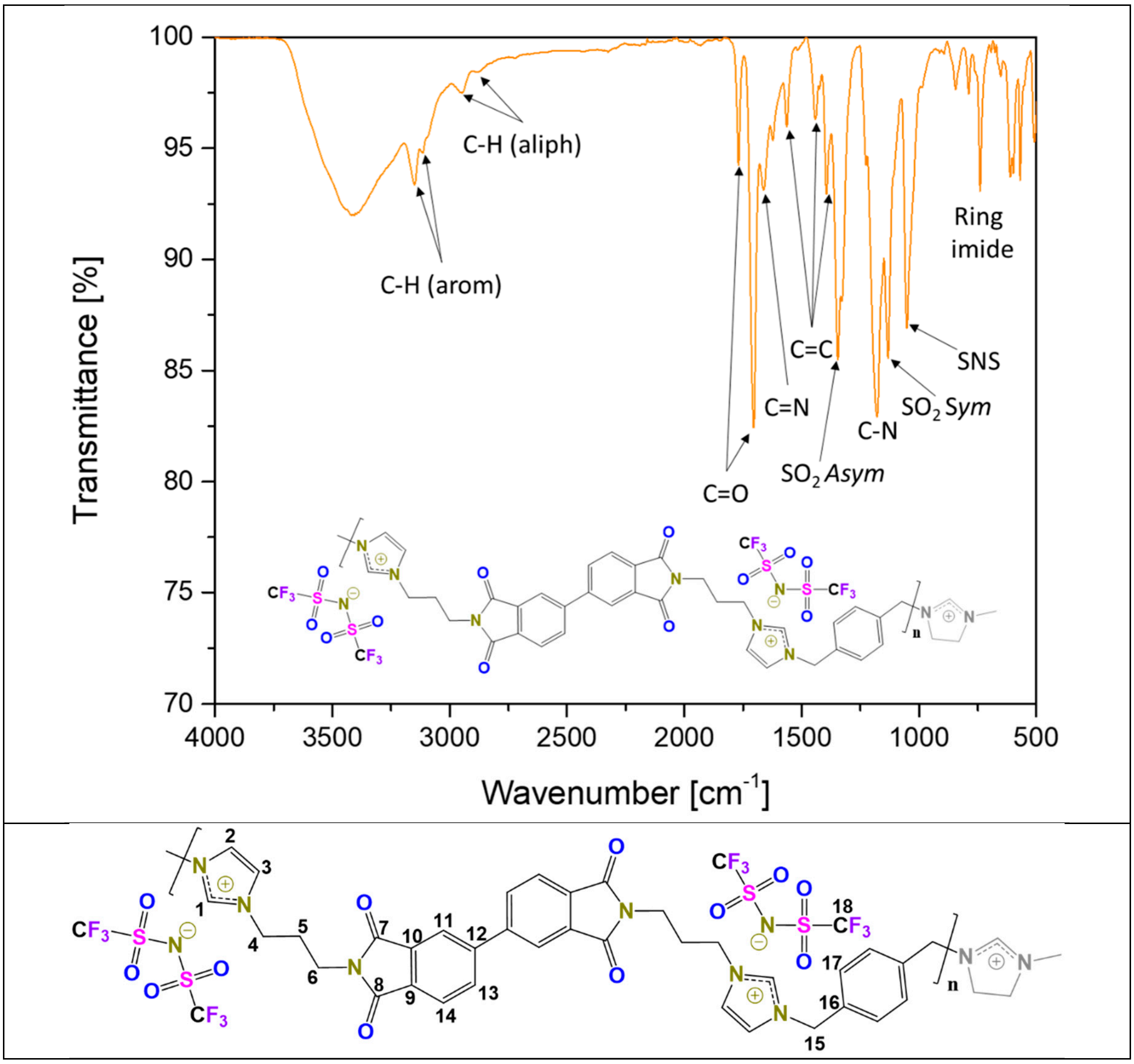
Transmittance [%]
100
95
90
85
80
75
70
4000
3500
3000
2500
2000
1500
1000
500
Wavenumber [cm-1]
C-H (arom)
C-H (aliph)
C=O
C=N
C=C
SO2 Asym
C-N
SO2 Sym
SNS
Ring imide

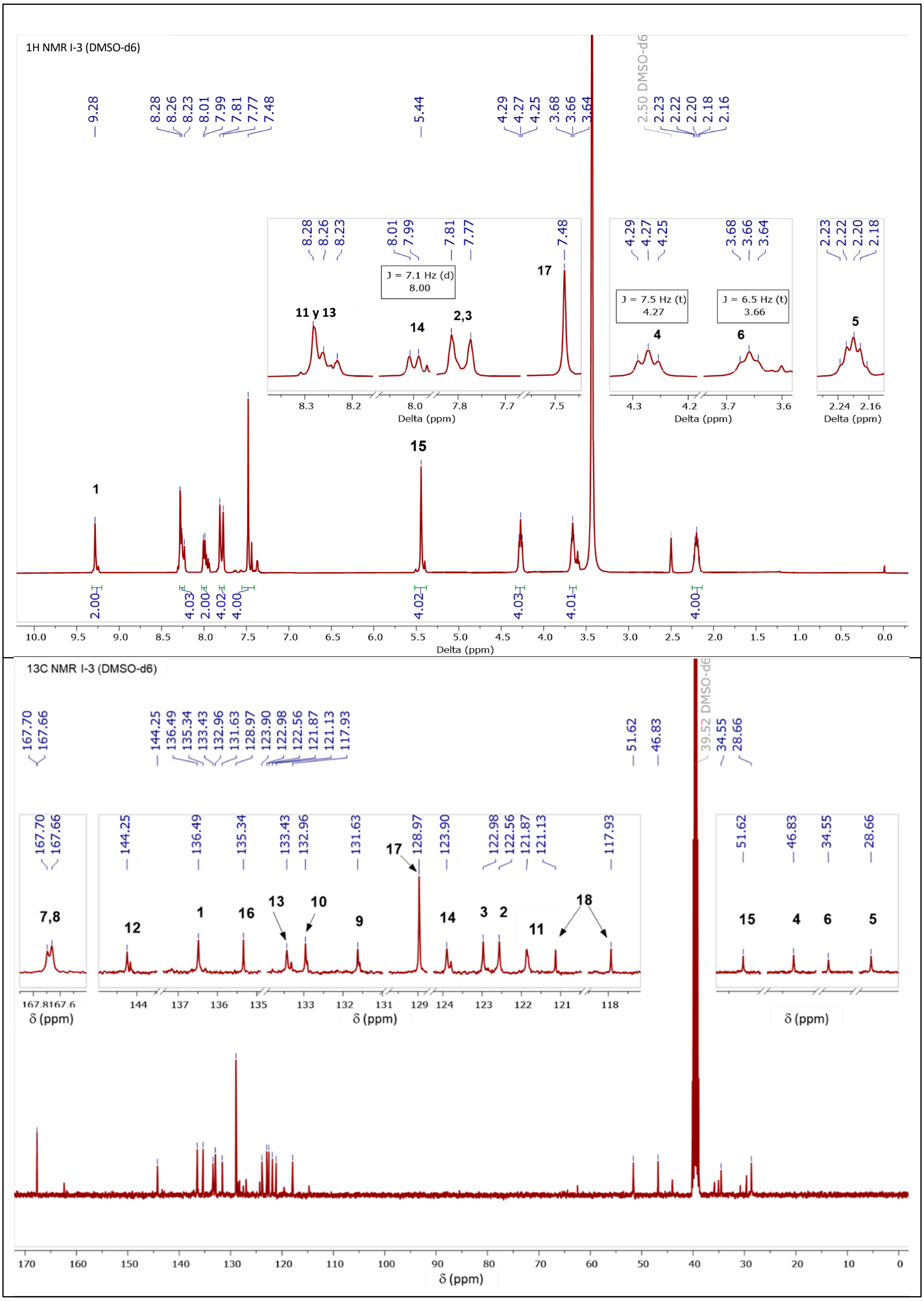
1H NMR I-3 (DMSO-d6)
9.28
8.28 8.26 8.23 8.01 7.99 7.81 7.77 7.48
5.44
4.29 4.27 4.25 3.68 3.66 3.64
2.50 DMSO-d6
2.23 2.22 2.20 2.18 2.16
J = 7.1 Hz (d) 8.00
J = 7.5 Hz (t) 4.27
J = 6.5 Hz (t) 3.66
11 y 13
14
2,3
17
4
6
5
15
1
2.00 4.03 2.00 4.02 4.00 4.02 4.03 4.01 4.00
Delta (ppm)
13C NMR I-3 (DMSO-d6)
167.70 167.66
144.25 136.49 135.34 133.43 132.96 131.63 128.97 123.90 122.98 122.56 121.87 121.13 117.93
51.62 46.83
39.52 DMSO-d6
34.55 28.66
7,8
12
1
16
13
10
9
17
14
3
2
11
18
15
4
6
5
δ (ppm)

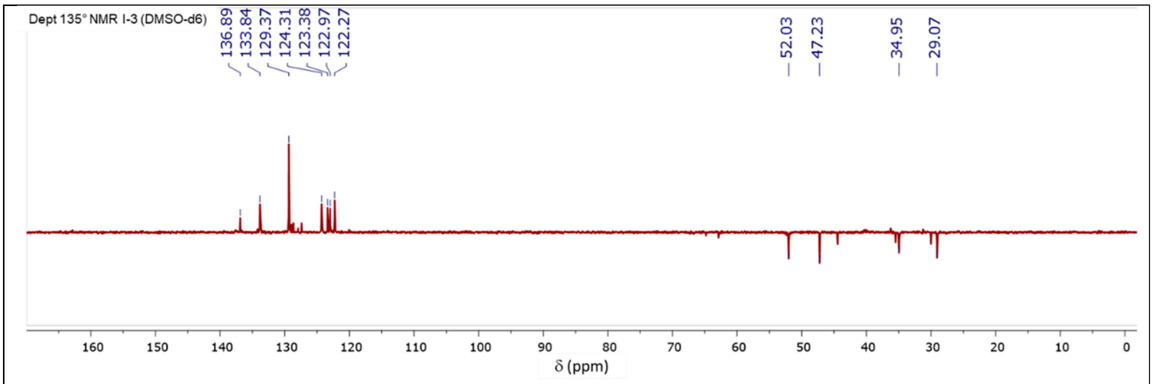

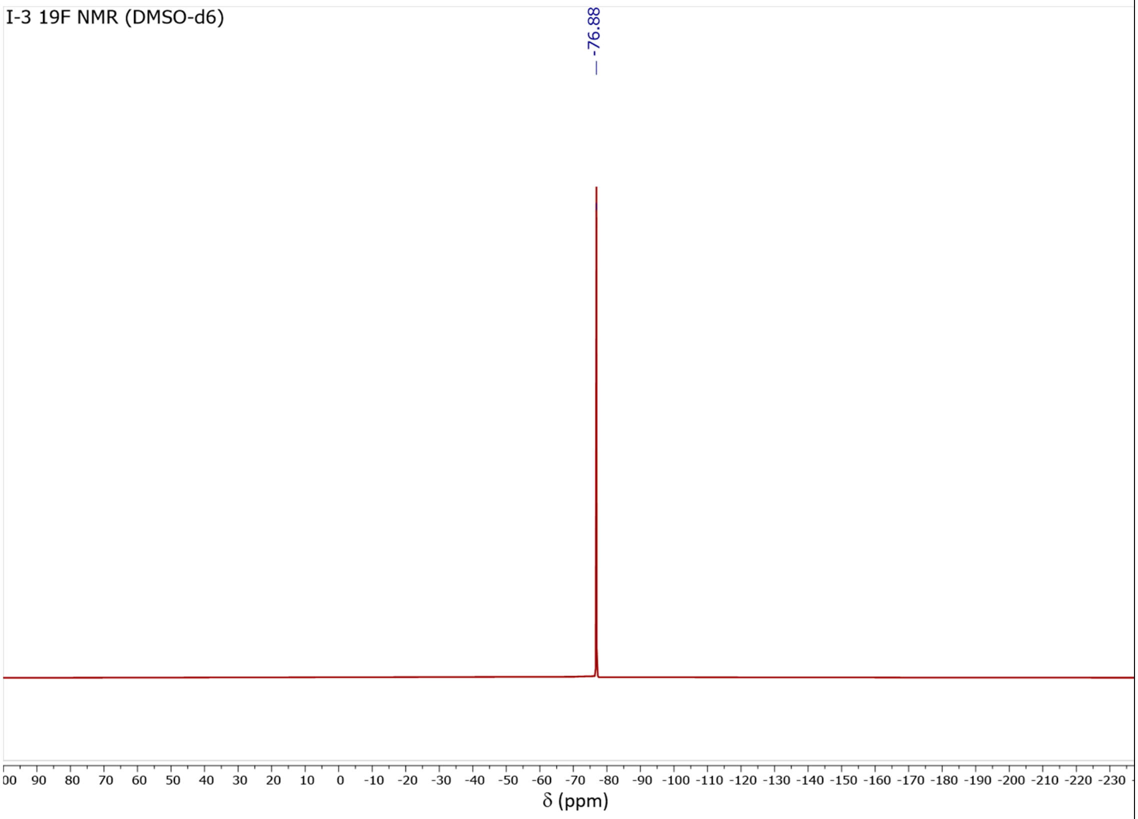

Figure S7. IR and ^{1}H, ^{13}C, Dept 135° and ^{19}F NMR spectra of ionene [BPDA-API-pXy] [NTf_2].

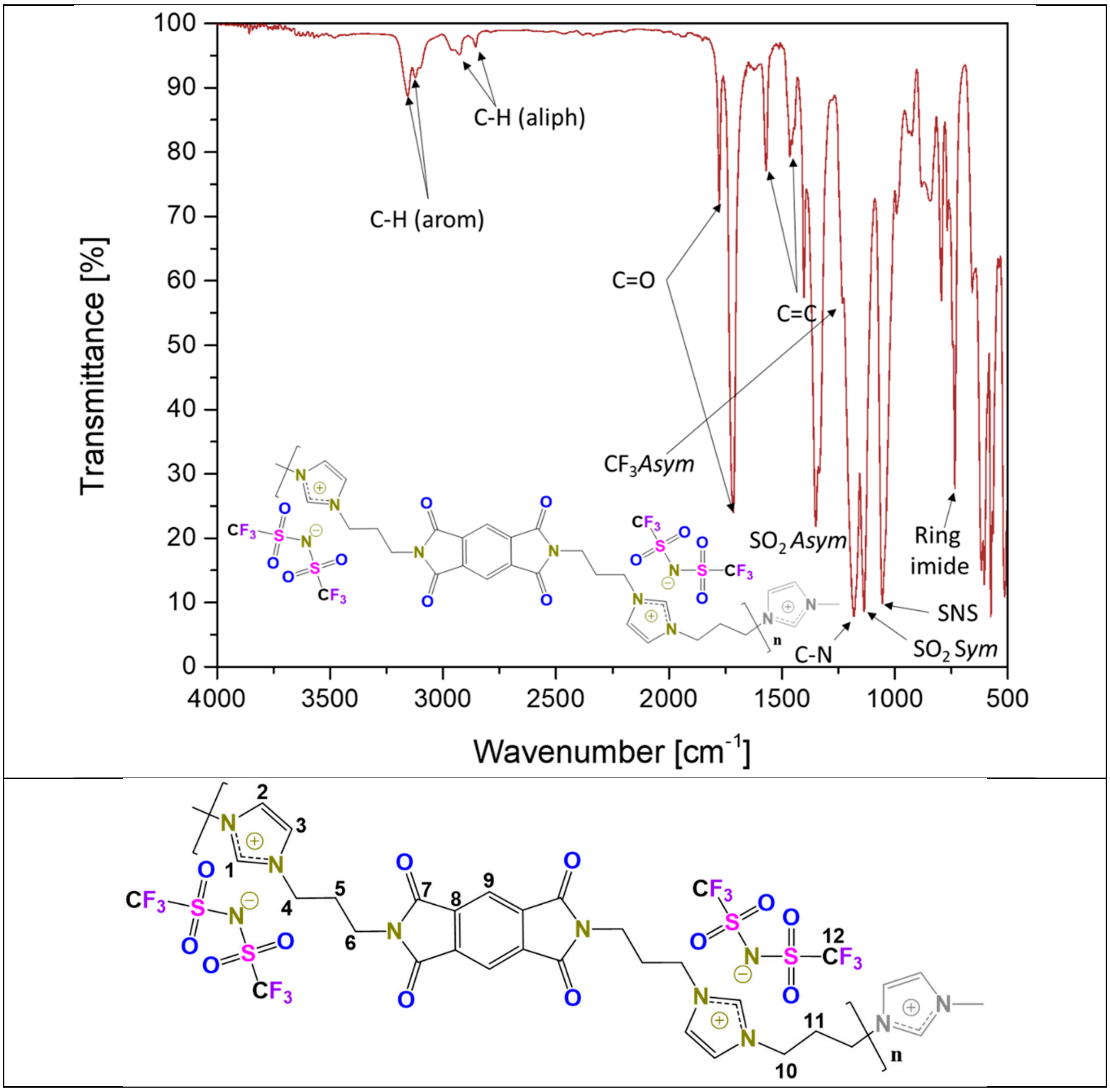

Transmittance [%]
Wavenumber [cm-1]
C-H (arom)
C-H (aliph)
C=O
C=C
CF3Asym
SO2 Asym
Ring imide
SNS
SO2 Sym
C-N

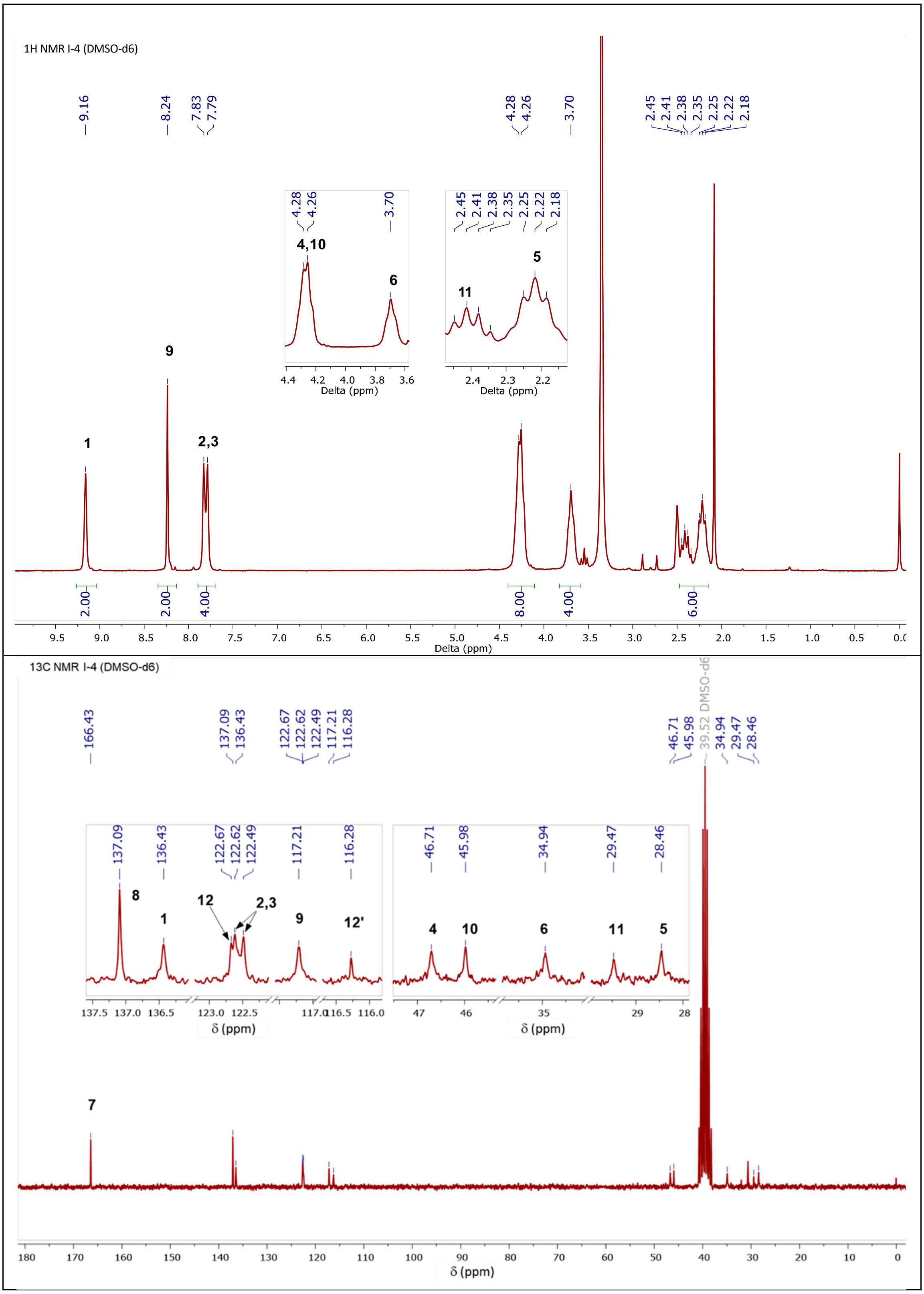
1H NMR I-4 (DMSO-d6)
9.16
8.24
7.83
7.79
4.28
4.26
3.70
2.45
2.41
2.38
2.35
2.25
2.22
2.18
4,10
6
11
5
9
1
2,3
2.00
2.00
4.00
8.00
4.00
6.00
Delta (ppm)
13C NMR I-4 (DMSO-d6)
166.43
137.09
136.43
122.67
122.62
122.49
117.21
116.28
46.71
45.98
39.52 DMSO-d6
34.94
29.47
28.46
8
1
12
2,3
9
12'
4
10
6
11
5
7
δ (ppm)

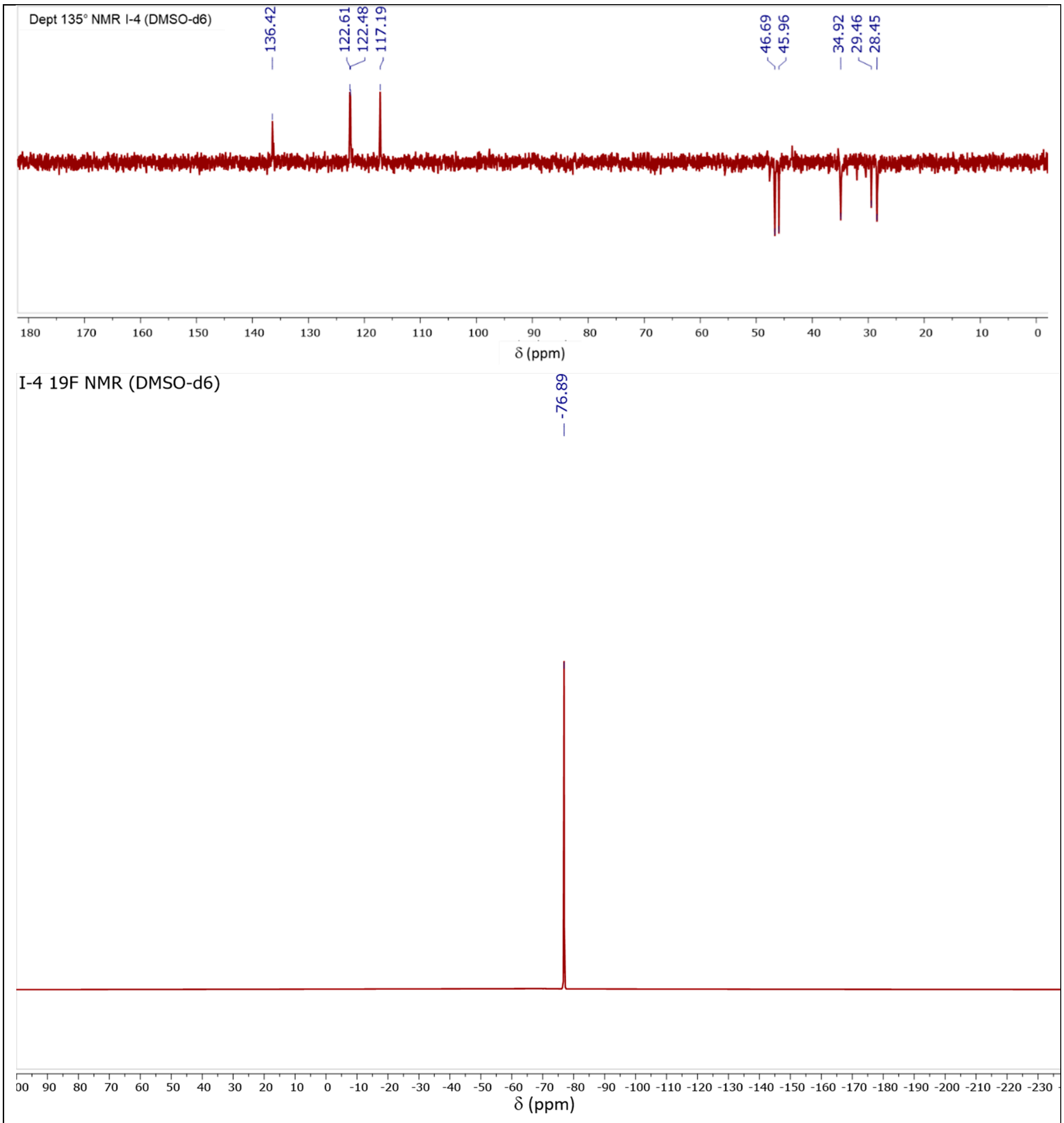

Figure S8. IR and ^{1}H, ^{13}C, Dept 135° and ^{19}F NMR spectra of ionene [PMDA-API-C_3] [NTf_2].

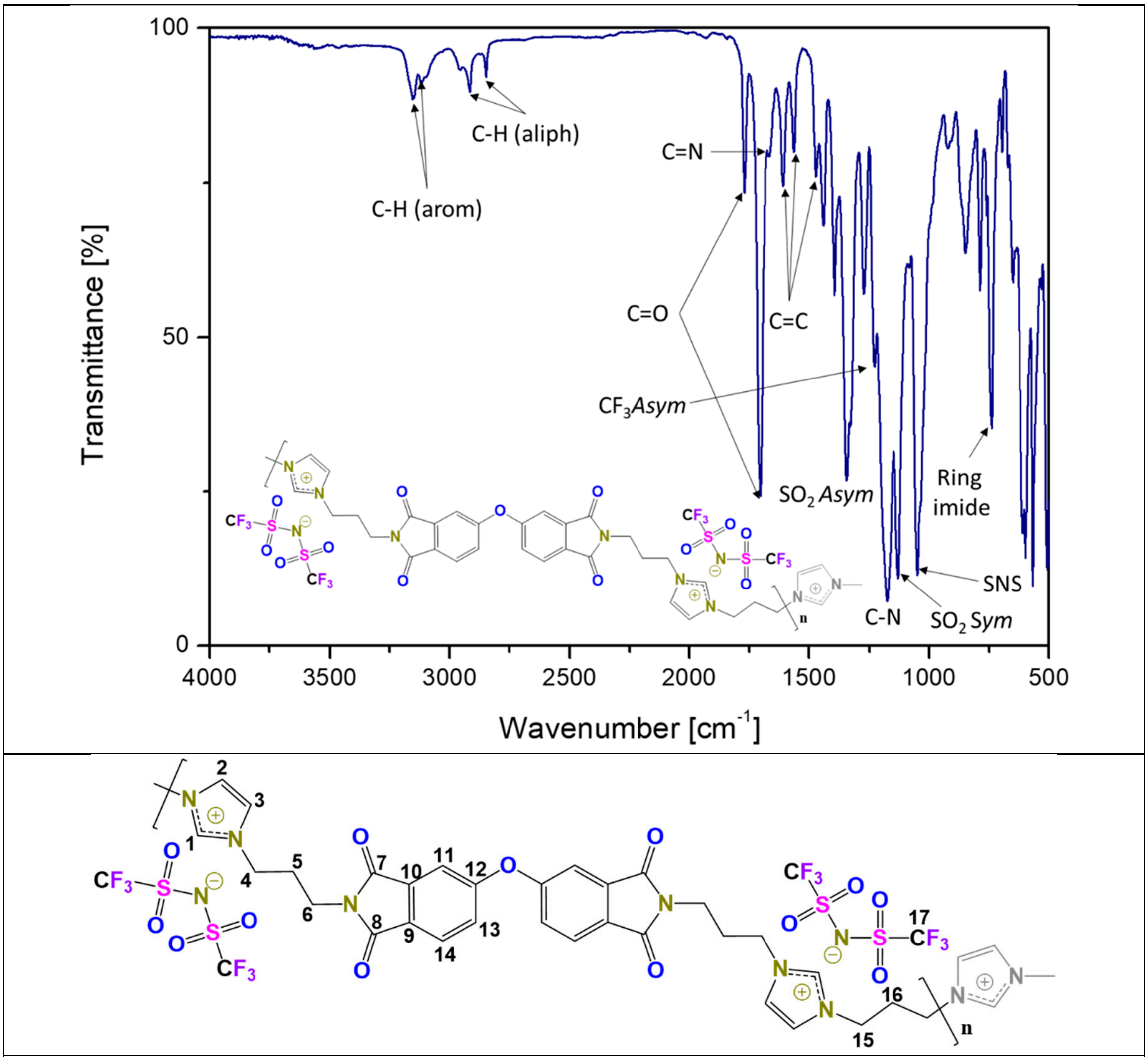

100
50
0
Transmittance [%]
4000
3500
3000
2500
2000
1500
1000
500
Wavenumber [cm-1]
C-H (arom)
C-H (aliph)
C=N
C=O
C=C
CF3Asym
SO2 Asym
Ring imide
C-N
SO2 Sym
SNS
n
1
2
3
4
5
6
7
8
9
10
11
12
13
14
15
16
17

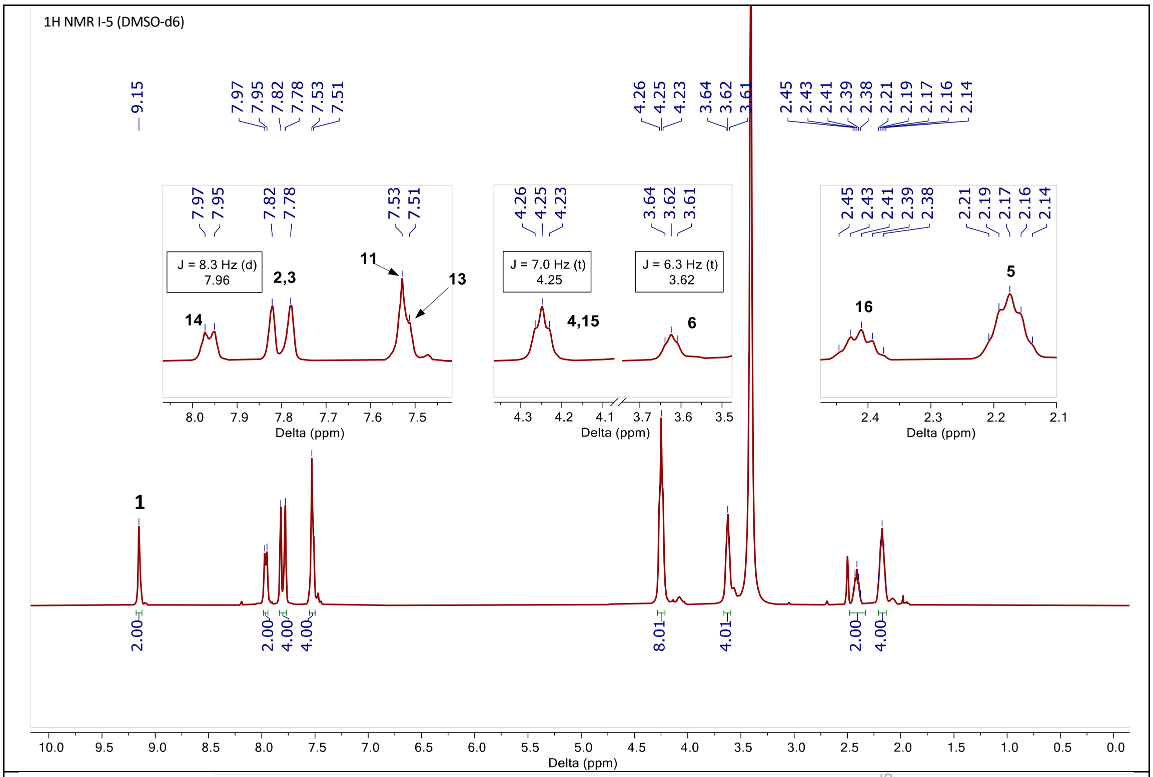
1H NMR I-5 (DMSO-d6)
J = 8.3 Hz (d)
7.96
J = 7.0 Hz (t)
4.25
J = 6.3 Hz (t)
3.62
Delta (ppm)

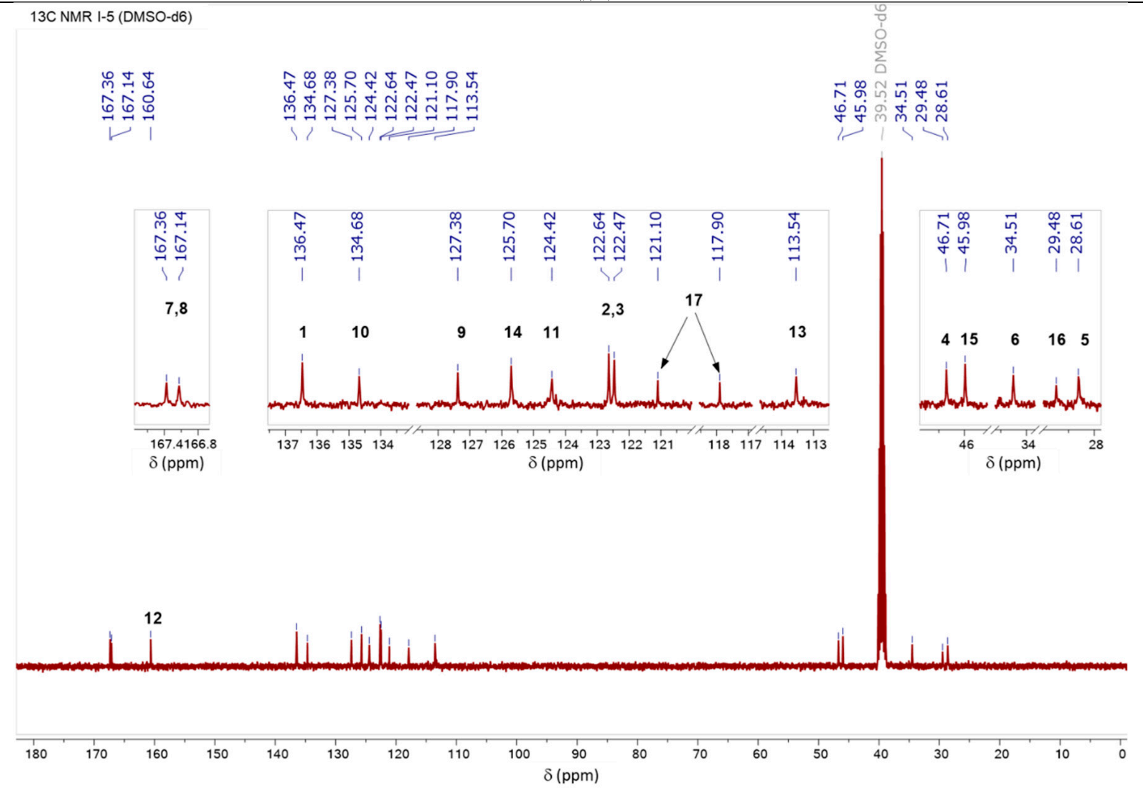
13C NMR I-5 (DMSO-d6)
39.52 DMSO-d6
δ (ppm)

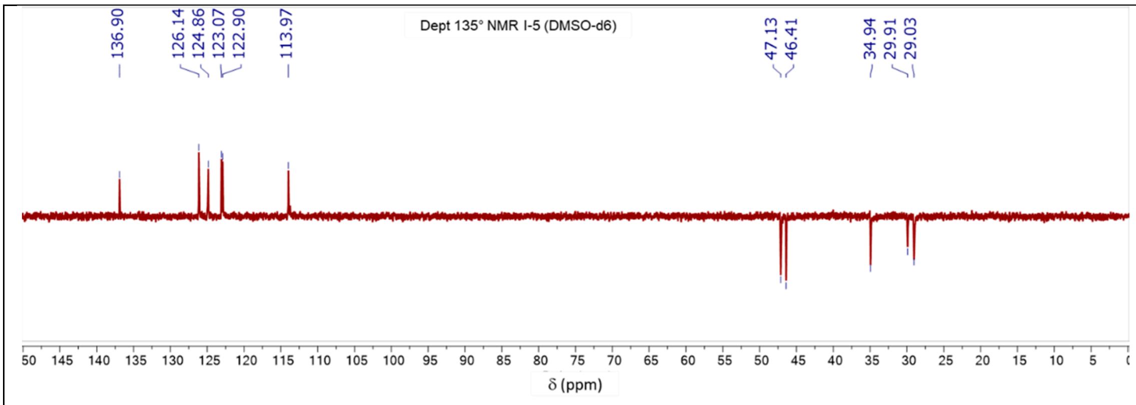

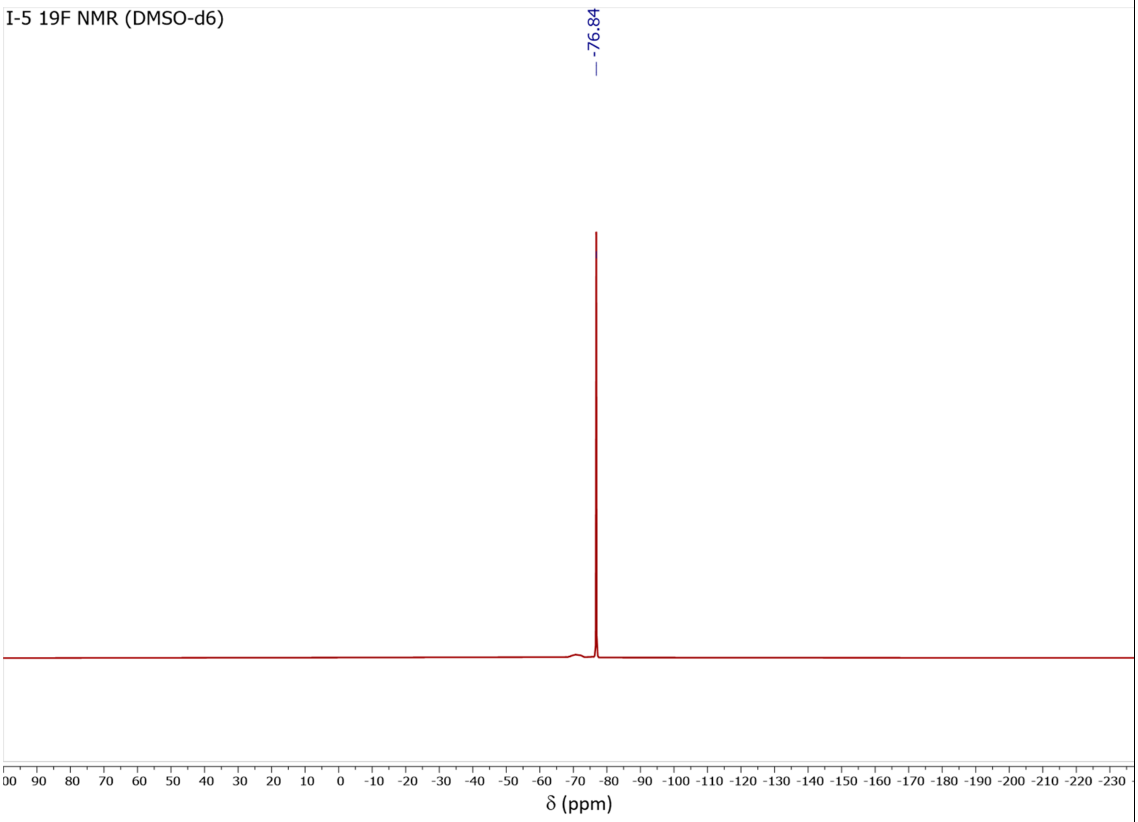

Figure S9. IR and ^{1}H, ^{13}C, Dept 135° and ^{19}F NMR spectra of ionene [ODPA-API-C_3] [NTf_2].

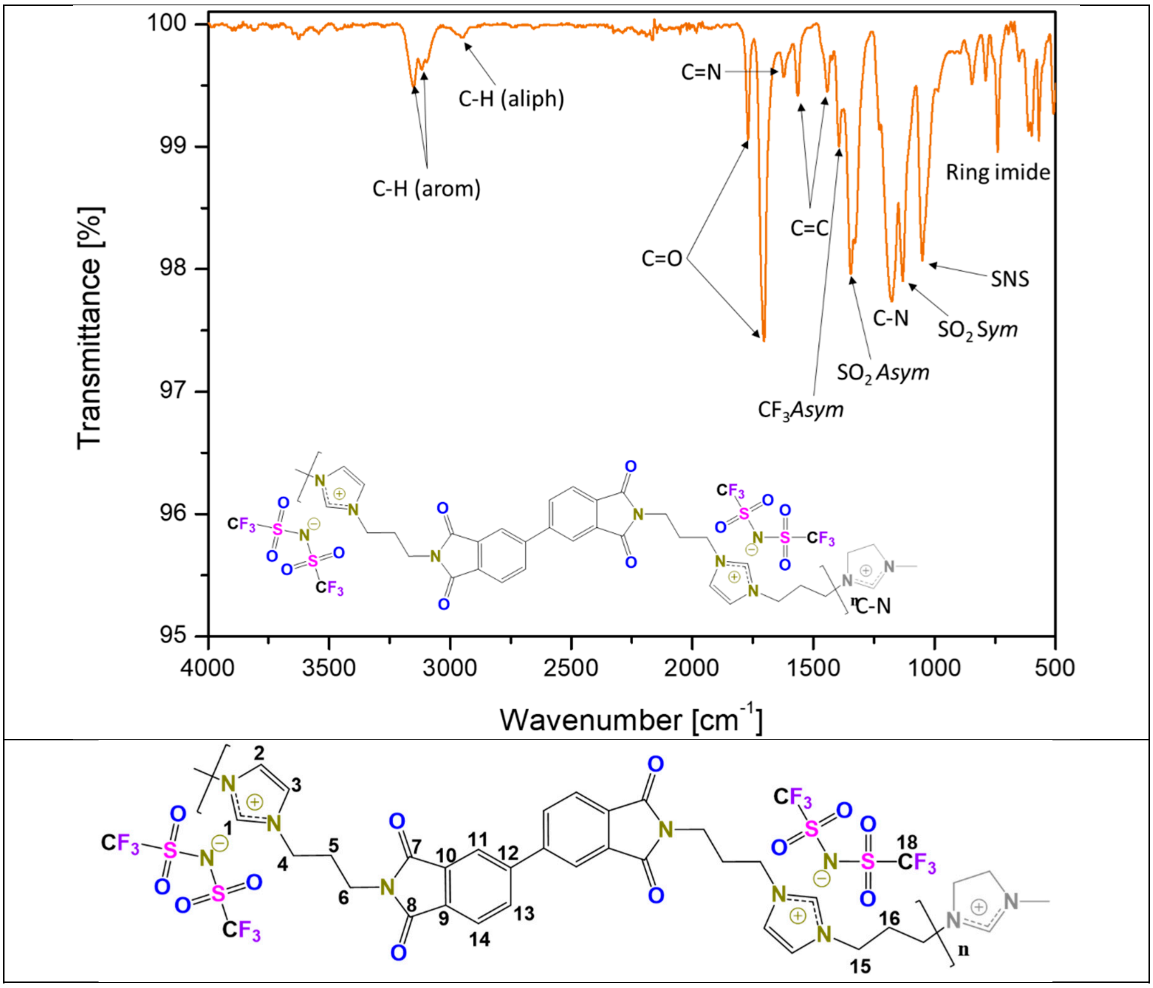
Transmittance [%]
Wavenumber [cm-1]
100
99
98
97
96
95
4000
3500
3000
2500
2000
1500
1000
500
C-H (arom)
C-H (aliph)
C=N
C=O
C=C
CF3Asym
SO2 Asym
C-N
SO2 Sym
SNS
Ring imide
C-N

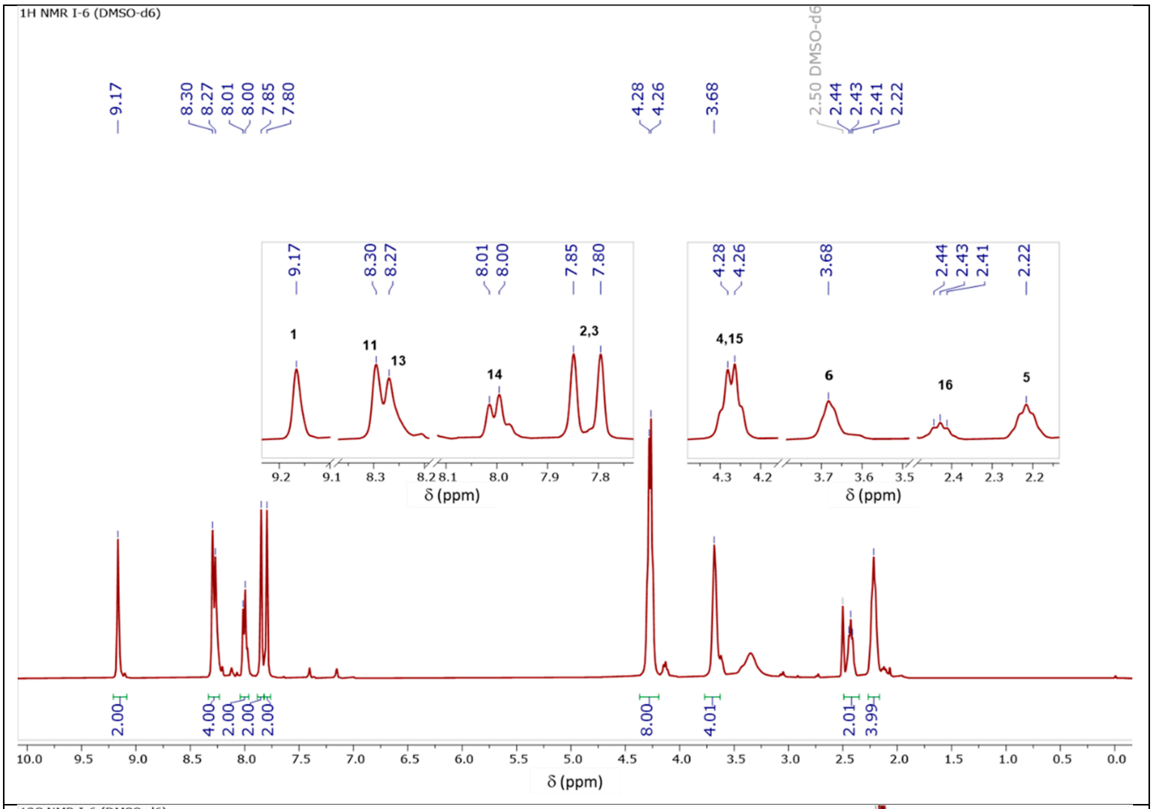
1H NMR I-6 (DMSO-d6)
9.17
8.30
8.27
8.01
8.00
7.85
7.80
4.28
4.26
3.68
2.50 DMSO-d6
2.44
2.43
2.41
2.22
1
11
13
14
2,3
4,15
6
16
5
2.00
4.00
2.00
2.00
2.00
8.00
4.01
2.01
3.99
δ (ppm)

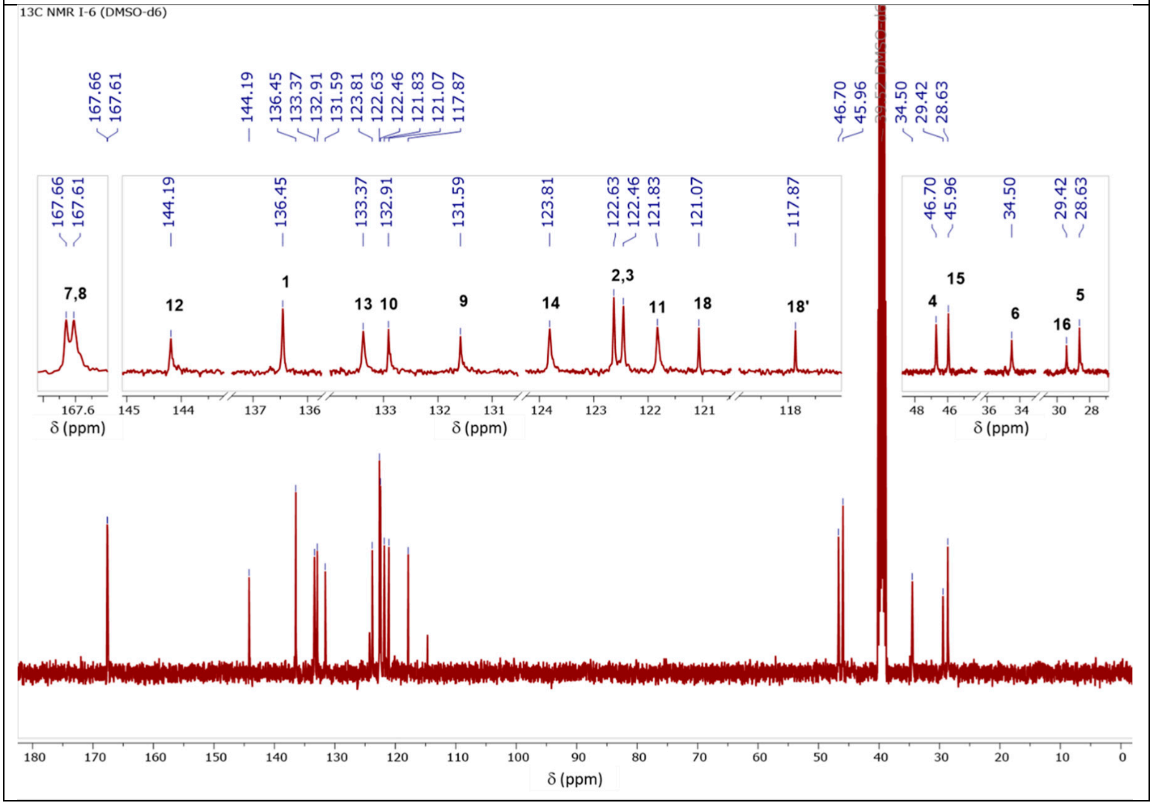
13C NMR I-6 (DMSO-d6)
167.66
167.61
144.19
136.45
133.37
132.91
131.59
123.81
122.63
122.46
121.83
121.07
117.87
46.70
45.96
34.50
29.42
28.63
7,8
12
1
13
10
9
14
2,3
11
18
18'
4
15
6
16
5
δ (ppm)

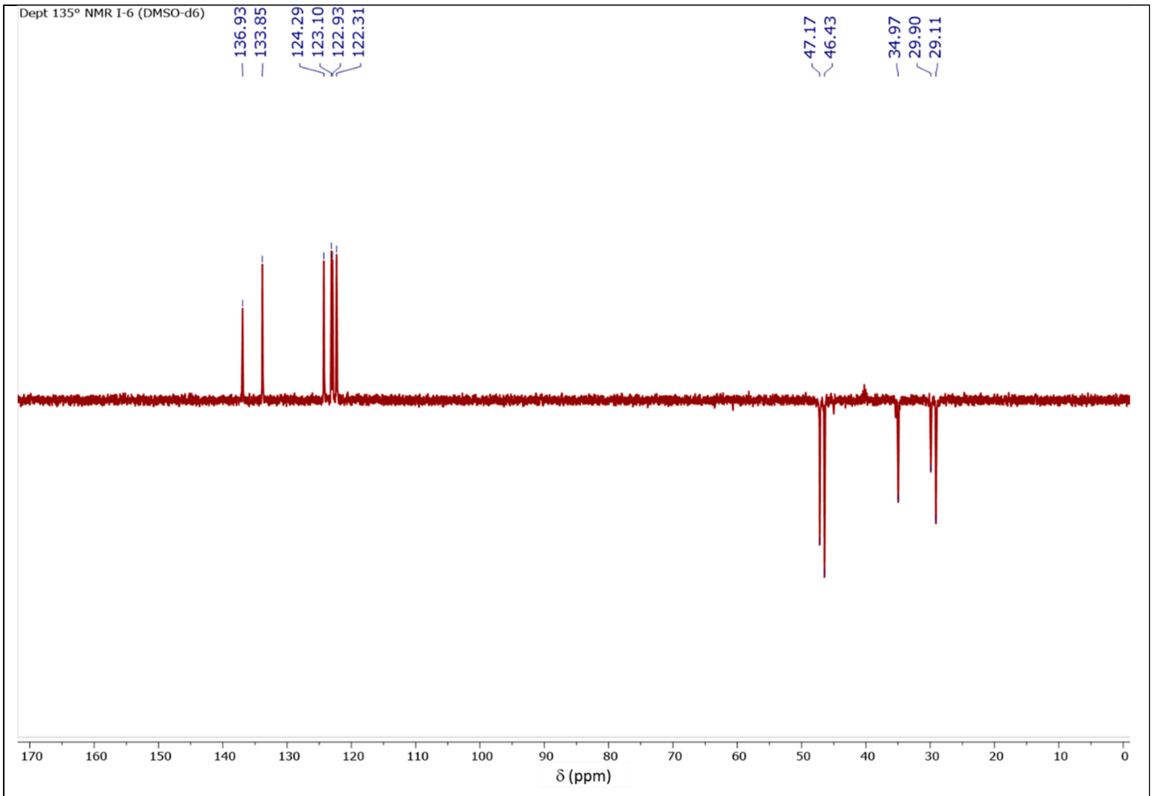

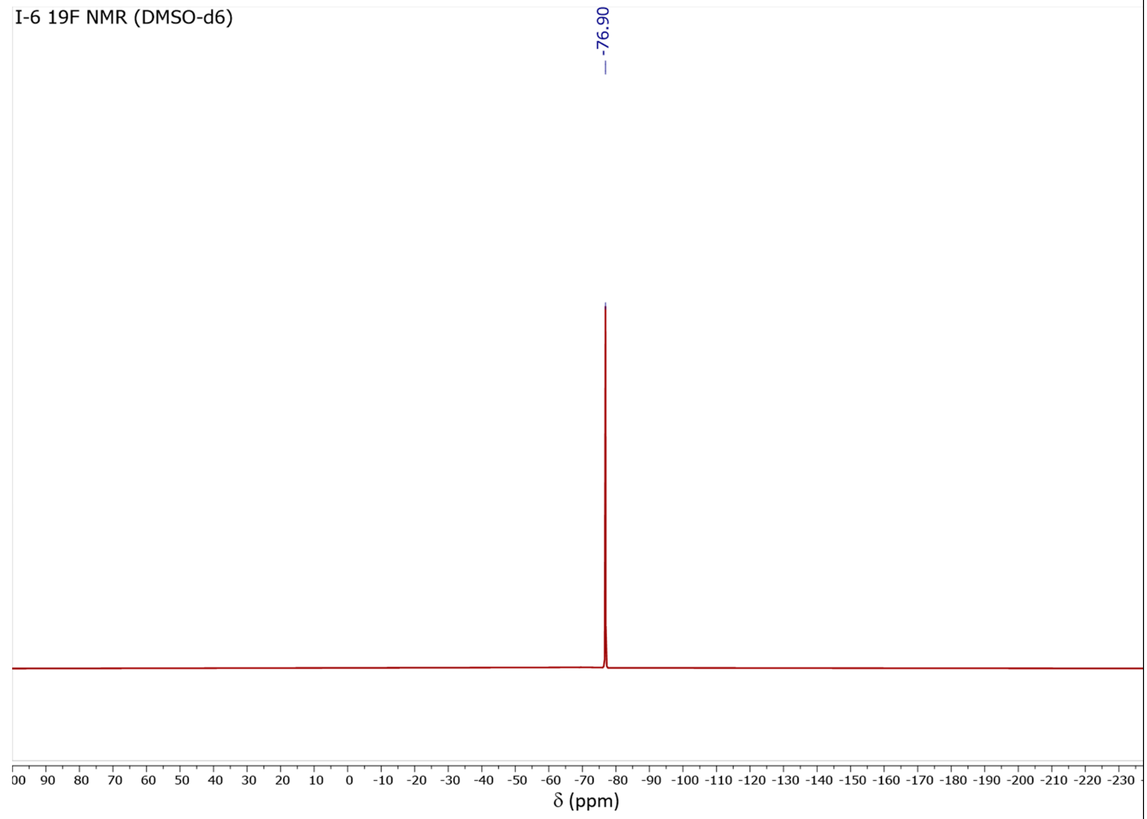

Figure S10. IR and ^{1}H, ^{13}C, Dept 135° and ^{19}F NMR spectra of ionene [BPDA-API-C_3] [NTf_2].

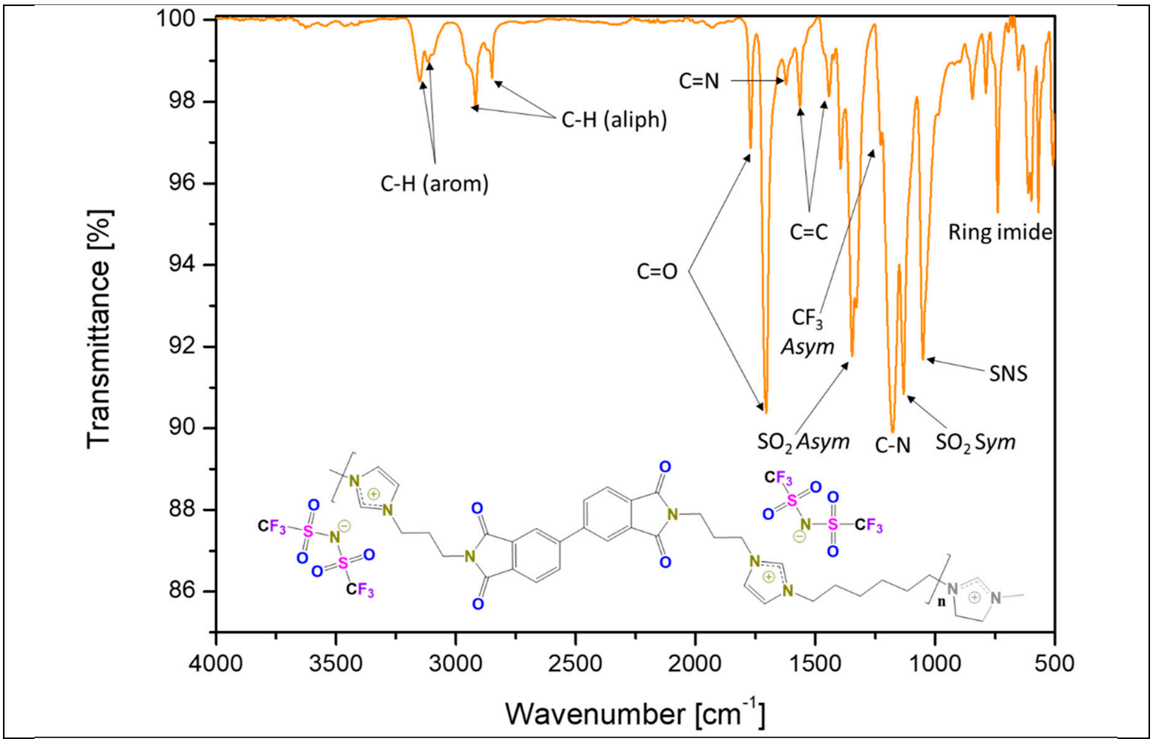

Transmittance [%]
Wavenumber [cm-1]
C-H (arom)
C-H (aliph)
C=N
C=O
C=C
CF3 Asym
SO2 Asym
C-N
SO2 Sym
SNS
Ring imide

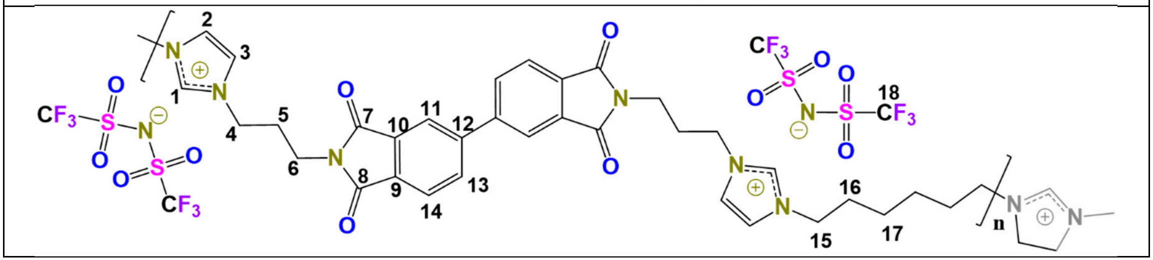

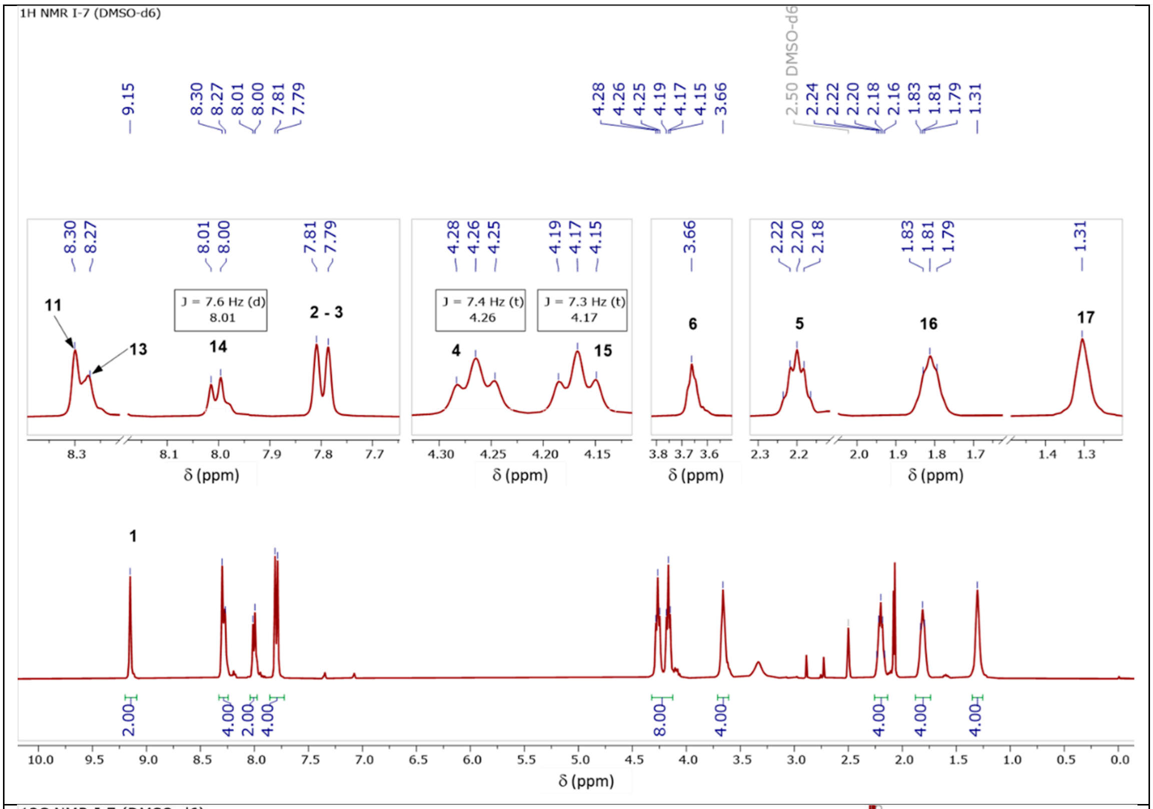
1H NMR I-7 (DMSO-d6)
9.15
8.30 8.27 8.01 8.00 7.81 7.79
4.28 4.26 4.25 4.19 4.17 4.15 3.66
2.50 DMSO-d6
2.24 2.22 2.20 2.18 2.16 1.83 1.81 1.79 1.31
11
13
14
2 - 3
J = 7.6 Hz (d)
8.01
4
15
J = 7.4 Hz (t)
4.26
J = 7.3 Hz (t)
4.17
6
5
16
17
1
2.00
4.00
2.00
4.00
8.00
4.00
4.00
4.00
4.00
δ (ppm)

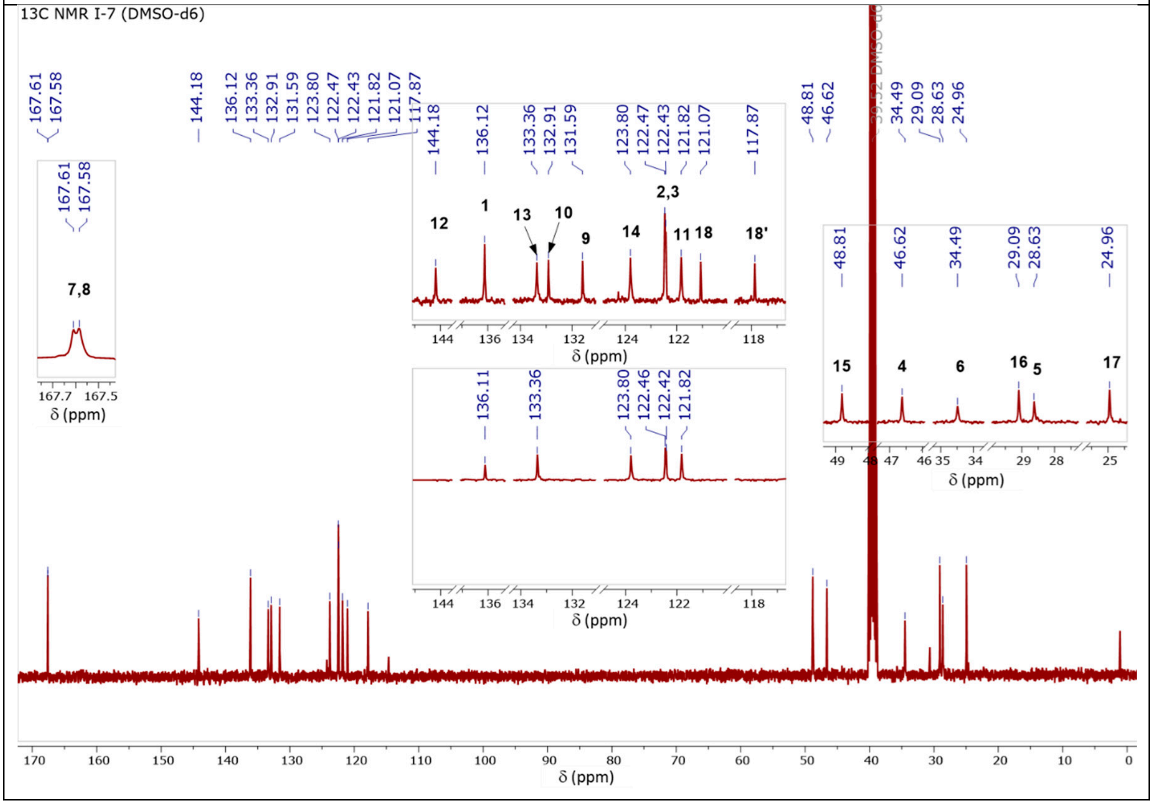
13C NMR I-7 (DMSO-d6)
167.61 167.58
144.18
136.12 133.36 132.91 131.59 123.80 122.47 122.43 121.82 121.07 117.87
48.81 46.62
39.52 DMSO-d6
34.49 29.09 28.63 24.96
7,8
12
1
13
10
9
14
2,3
11
18
18'
136.11 133.36 123.80 122.46 122.42 121.82
15
4
6
16
5
17
δ (ppm)

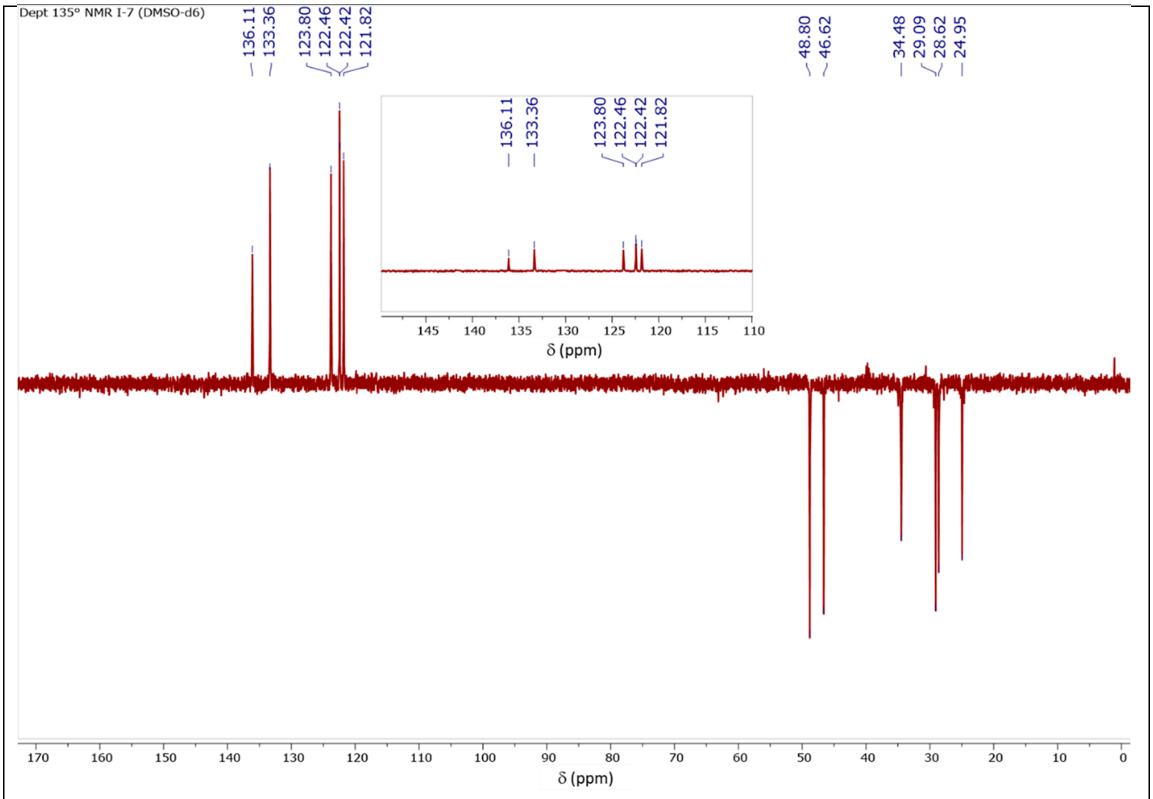
Dept 135° NMR I-7 (DMSO-d6)
136.11
133.36
123.80
122.46
122.42
121.82
48.80
46.62
34.48
29.09
28.62
24.95
δ (ppm)

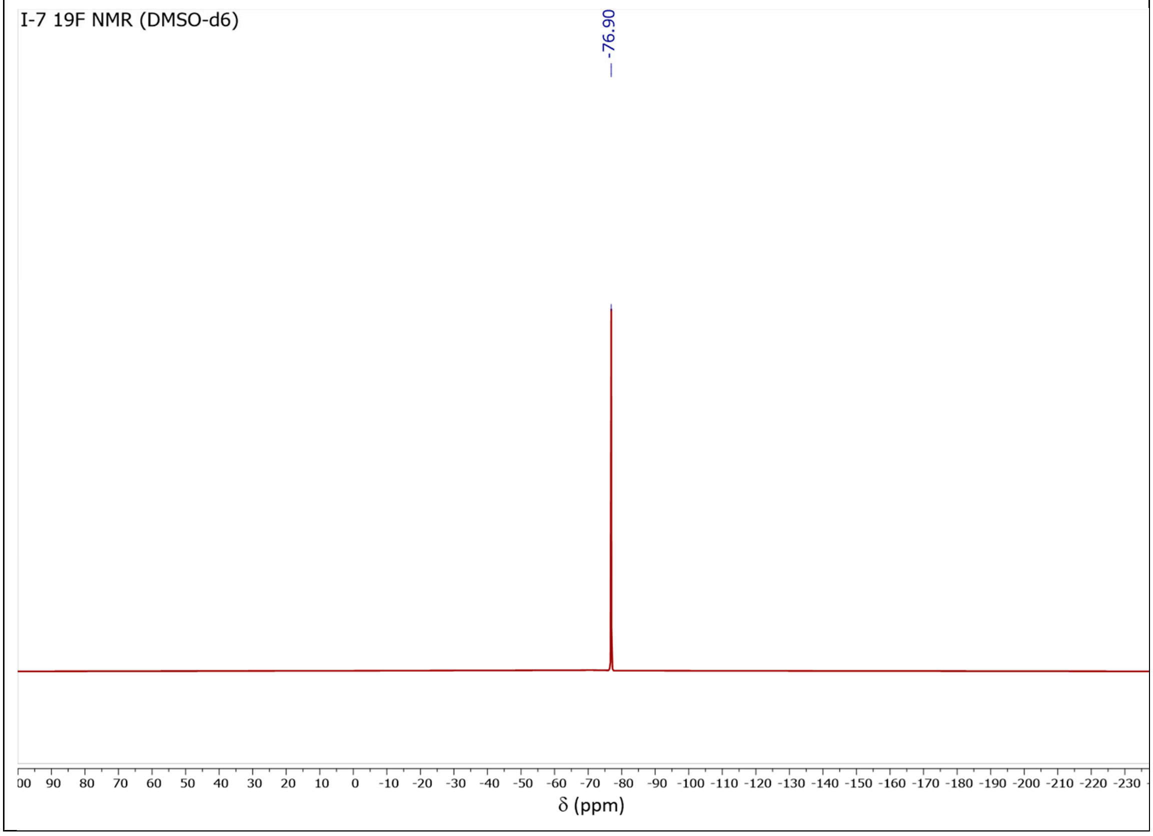
I-7 19F NMR (DMSO-d6)
-76.90
δ (ppm)

Figure S11. IR and ^{1}H, ^{13}C, Dept 135° and ^{19}F NMR spectra of ionene [BPDA-API-C_6] [NTf_2].

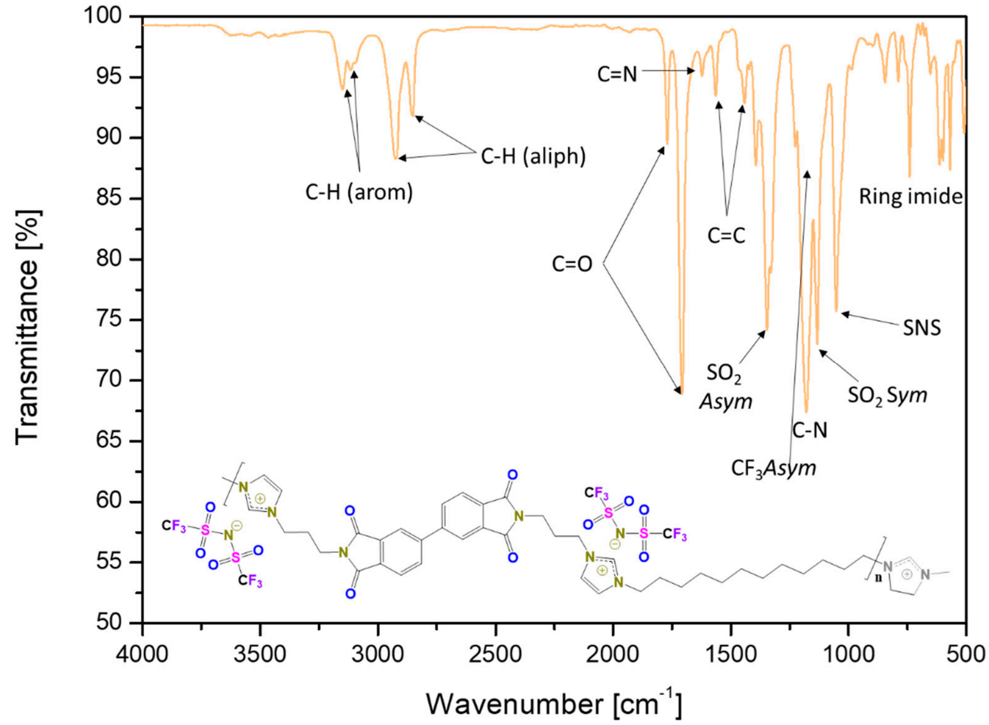

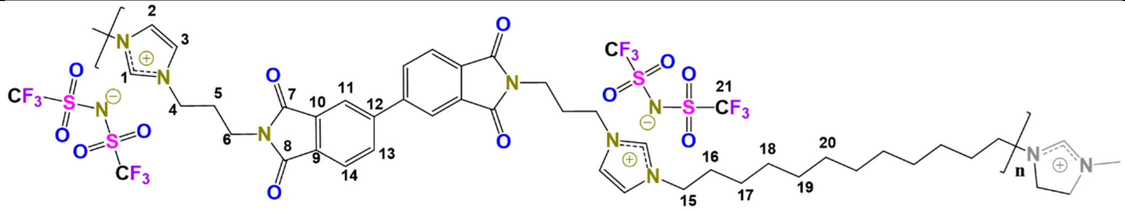

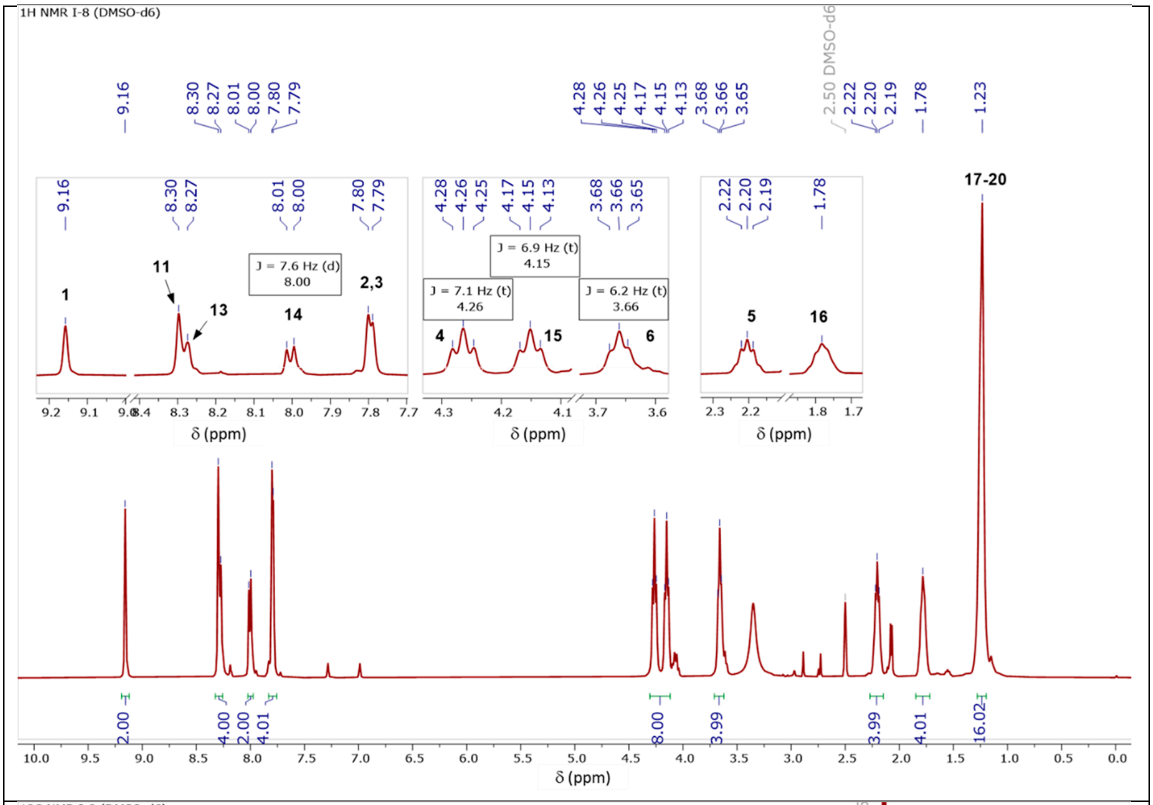
1H NMR I-8 (DMSO-d6)
2.50 DMSO-d6
J = 7.6 Hz (d) 8.00
J = 6.9 Hz (t) 4.15
J = 7.1 Hz (t) 4.26
J = 6.2 Hz (t) 3.66
17-20
δ (ppm)

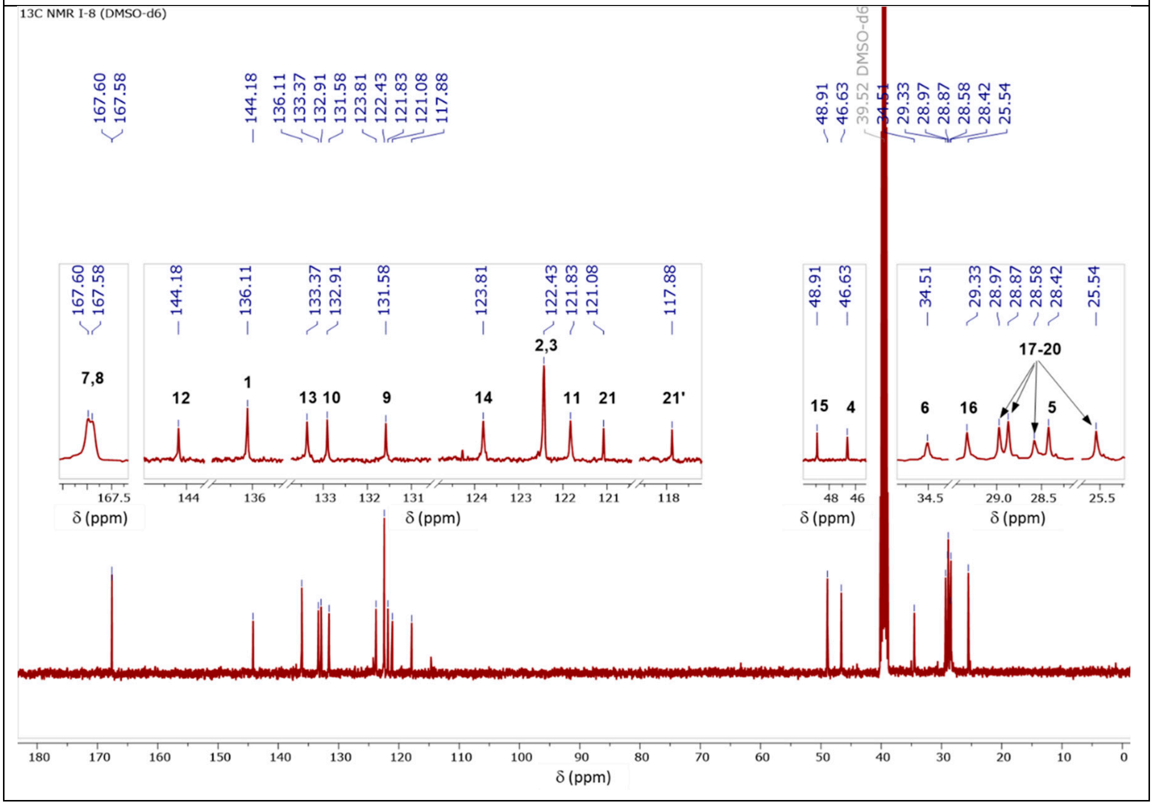
13C NMR I-8 (DMSO-d6)
39.52 DMSO-d6
17-20
δ (ppm)

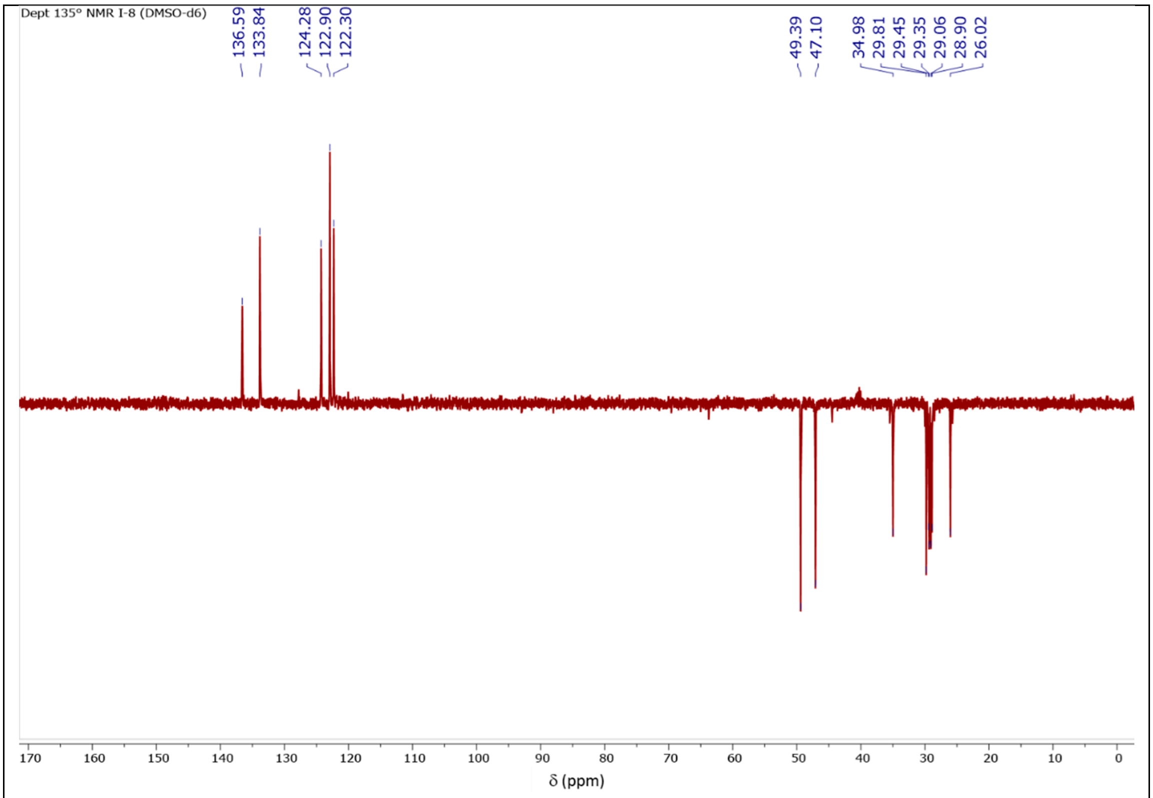

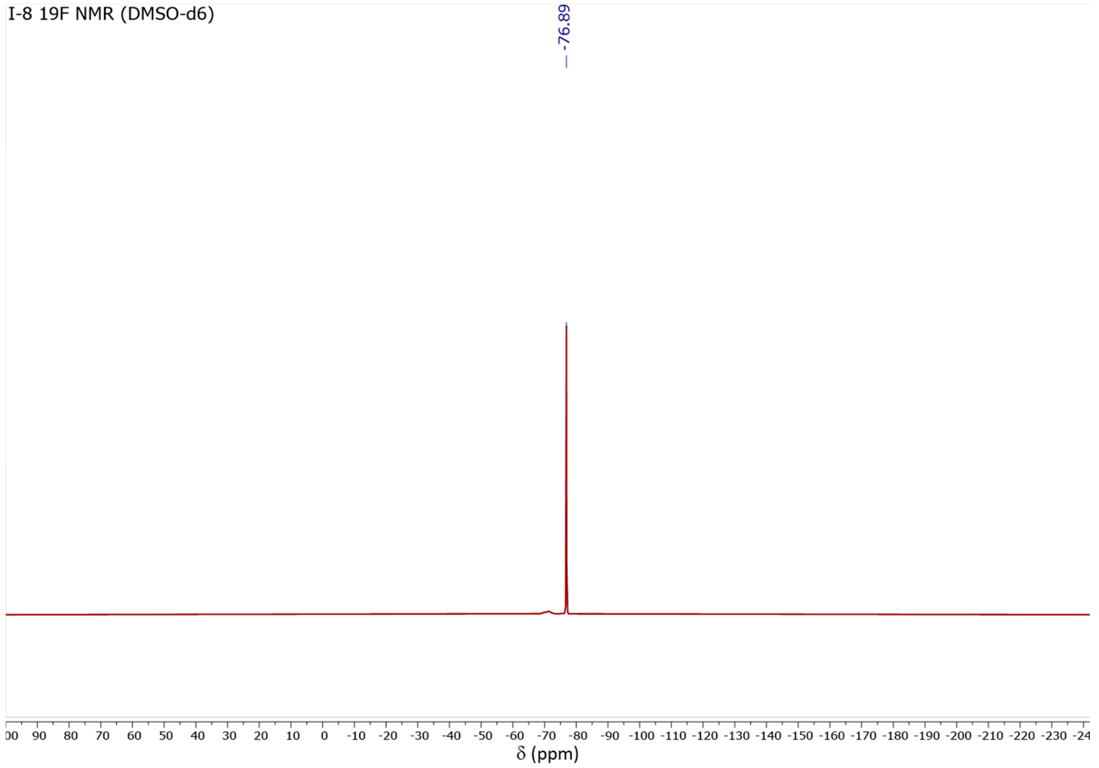

Figure S12. IR and ^{1}H, ^{13}C, Dept 135° and ^{19}F NMR spectra of ionene [BPDA-API-C_{12}] [NTf_2].

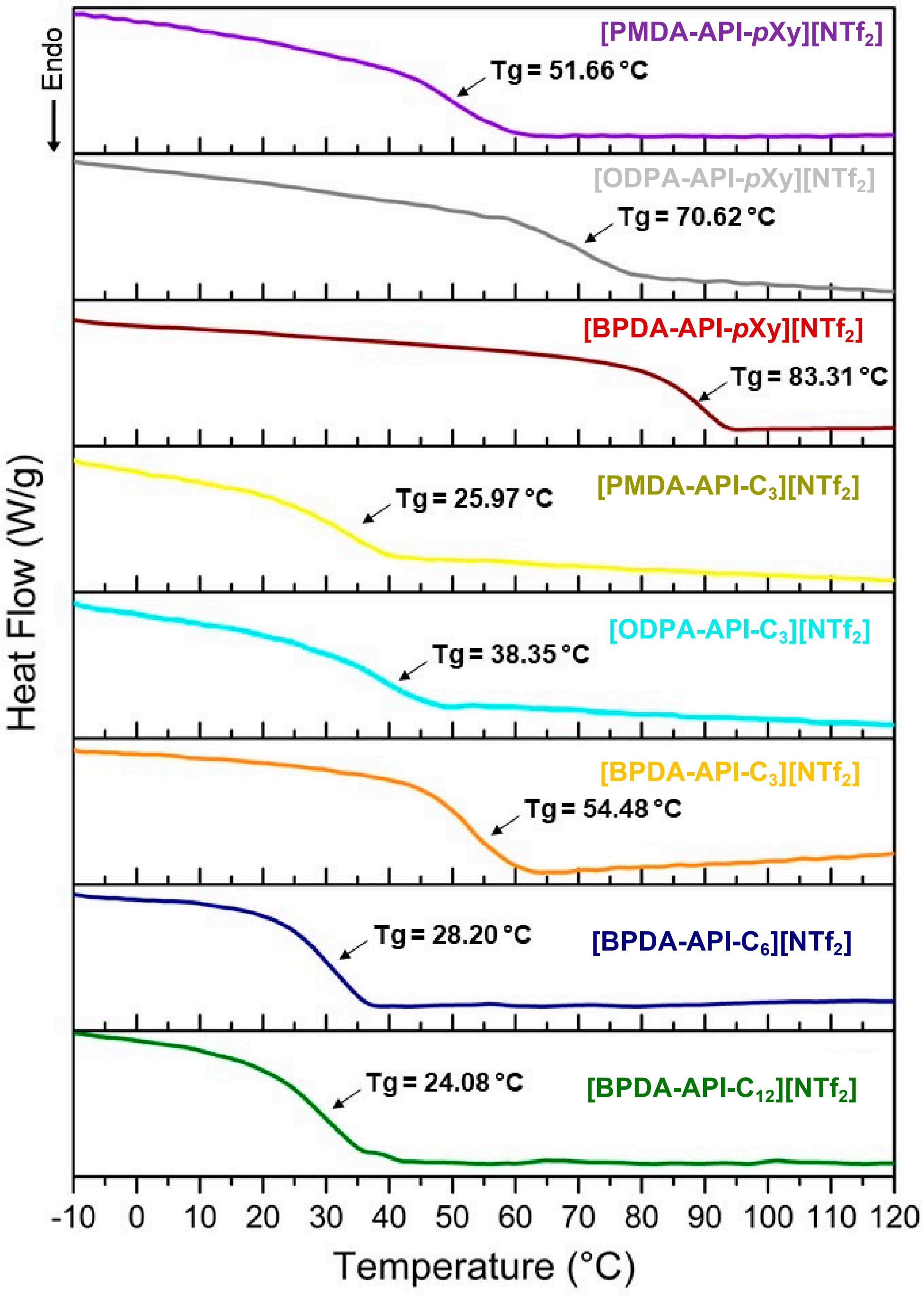

Figure S13. Heating curves of the DSC assays of the synthesized ionenes.

	**Water**	**CH_2I_2**
[PMDA-API-*p*Xy][NTf_2]	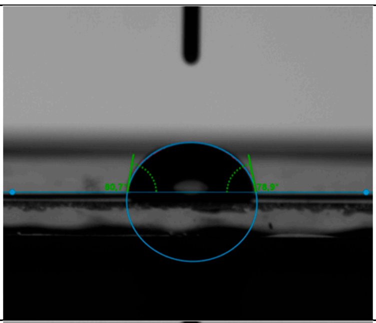	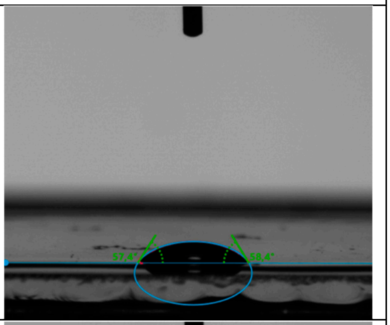
[ODPA-API-*p*Xy][NTf_2]	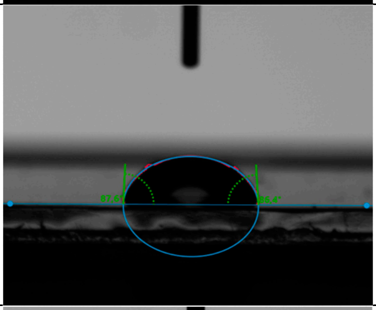	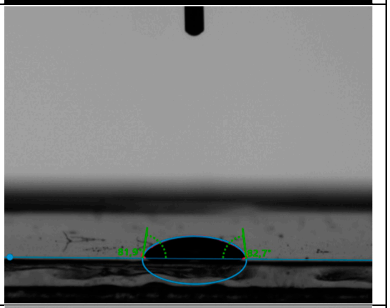
[BPDA-API-*p*Xy] [NTf_2]	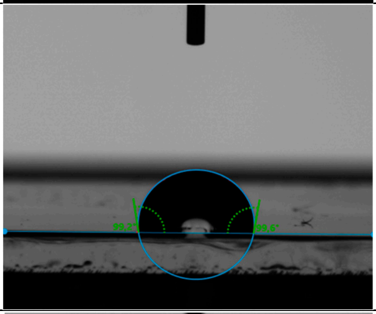	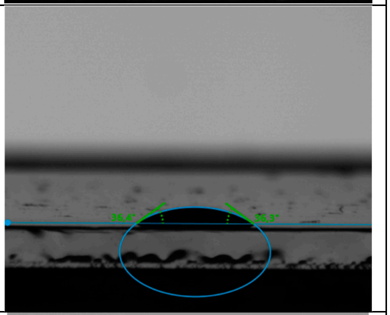
[PMDA-API-C_3] [NTf_2]	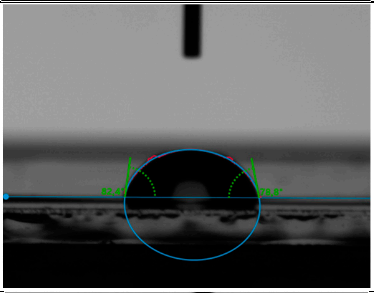	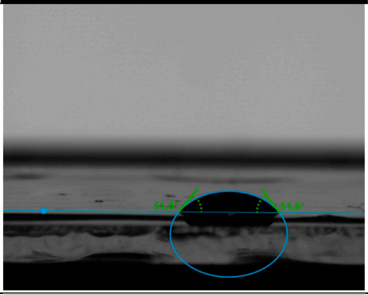
[ODPA-API-C_3] [NTf_2]	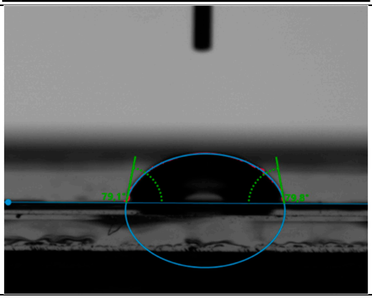	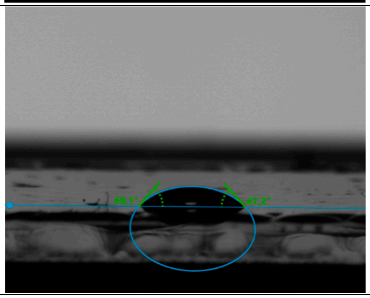

[BPDA-API-C_3] [NTf_2]	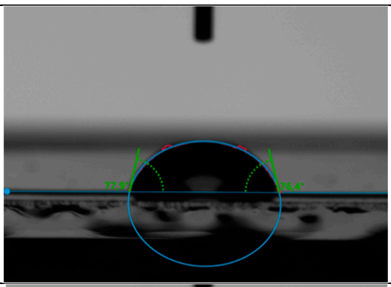	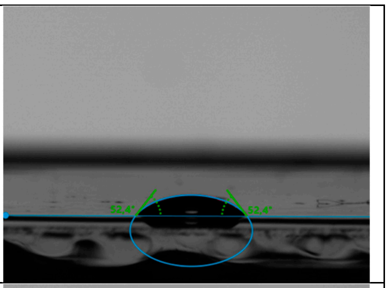
[BPDA-API-C_6] [NTf_2]	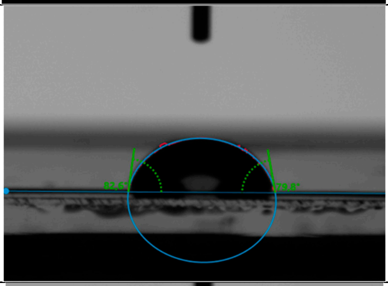	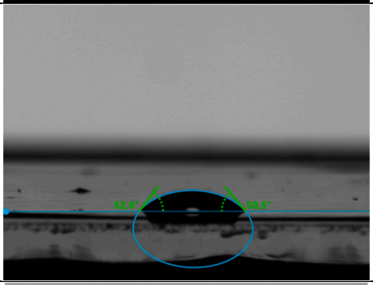
[BPDA-API-C_{12}] [NTf_2]	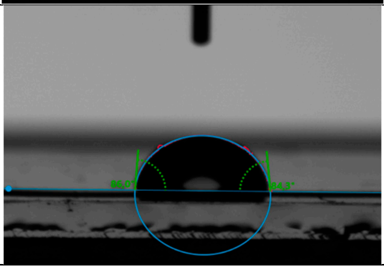	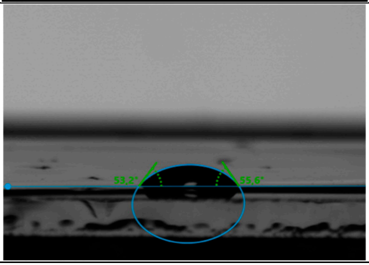

Figure S14. Some contact angle measurements for the synthesized ionenes.
